# Antimicrobial and Cytotoxic Cyathane-Xylosides from Cultures of the Basidiomycete *Dentipellis fragilis*

**DOI:** 10.3390/antibiotics11081072

**Published:** 2022-08-08

**Authors:** Winnie Chemutai Sum, Nico Mitschke, Hedda Schrey, Kathrin Wittstein, Harald Kellner, Marc Stadler, Josphat Clement Matasyoh

**Affiliations:** 1 Department Microbial Drugs, Helmholtz Centre for Infection Research GmbH (HZI), Inhoffenstraße 7, 38124 Braunschweig, Germany; 2 Research Group for Marine Geochemistry (ICBM-MPI Bridging Group), Institute for Chemistry and Biology of the Marine Environment (ICBM), Carl von Ossietzky Universität Oldenburg, Carl-von-Ossietzky-Str. 9-11, 26129 Oldenburg, Germany; 3 Department of Bio- and Environmental Sciences, Technische Universität Dresden—International Institute Zittau, Markt 23, 02763 Zittau, Germany; 4 Institute of Microbiology, Technische Universität Braunschweig, Spielmannstraße 7, 38106 Braunschweig, Germany; 5 Department of Chemistry, Egerton University, P.O. Box 536, Njoro 20115, Kenya

**Keywords:** cyathane-xylosides, *Dentipellis fragilis*, dentifragilins, Hericiaceae

## Abstract

In our continued search for biologically active metabolites from cultures of rare Basidiomycota species, we found eight previously undescribed cyathane-xylosides from submerged cultures of *Dentipellis fragilis*, which were named dentifragilins A–H. In addition, the known cyathane derivatives striatal D and laxitextine A were isolated. All compounds were characterized by high-resolution electrospray ionization mass spectrometry (HR-ESIMS) as well as by 1D and 2D nuclear magnetic resonance (NMR) spectroscopy. Several of the compounds exhibited significant activities in standardized cell-based assays for the determination of antimicrobial and cytotoxic effects. The discovery of cyathanes in the genus *Dentipellis* has chemotaxonomic implications, as this class of diterpenoids has already been shown to be characteristic for mycelial cultures of the related genera *Hericium* and *Laxitextum*, which are classified as *Dentipellis* in the family Hericiaceae.

## 1. Introduction

Over the past decades, fungi have been one of the most prolific sources for innovative chemistry. Many compounds that eventually became blockbuster drugs were discovered from their mycelial cultures, beginning with the groundbreaking discovery of penicillin [1]. Fungi can be very useful in other fields of biotechnology as well [2]. However, thus far no attempts have been made to cultivate the vast majority of species, and for many this has never even been explored, making them unavailable for study of their capability to produce potentially interesting metabolites and enzymes.

While most industrially utilized and studied fungal producers belong to the Ascomycota (including yeasts and common soil-derived molds), the division Basidiomycota remains underexplored [3]. Recent reviews by Sandargo et al. [3] and Gressler et al. [4] have summarized the state of the art on the exploitation of their secondary metabolites and highlighted the fact that these organisms have the ability to produce many unique compounds derived from specific biosynthetic pathways. In particular, the class Agaricomycetes, which comprises most of the fungi that are widely summarized under the non-scientific term “mushrooms”, has already been proved to be prolific with respect to the production of biologically active compounds. Examples are the pleuromutilins, which constitute one of the last classes of antibacterial antibiotics that have been approved for human therapy, and the illudins, which have given rise to the development of several clinical candidates for anticancer drugs [3].

In recent years, we have started a research program to systematically target the Basidiomycota for their potential to produce beneficial metabolites. Our strategy is mainly based on two rationales. On the one hand, we are working on species from hitherto unexplored geographic regions such as the African tropics and the rainforests of Northern Thailand, from which we have already reported several unique bioactive molecules [5,6,7]. On the other hand, we are studying rare species of the European mycobiota that are apparently unexplored because they are rarely found in nature, and hence have not been cultured. One striking example is *Rhodotus palmatus*, from which several unprecedented and rare terpenoids were recently reported [8]. The present study is aimed at the evaluation of *Dentipellis fragilis*, which is very uncommonly reported in Central Europe, even though it is more frequently recorded in other geographic areas such as Scandinavia (see https://www.gbif.org/species/9066731 (accessed on 3 August 2022)). The basidiomes of this rather inconspicuous resupinate mushroom normally occur on beechwood, and are characterized by a hydnoid hymenium [9,10,11]. Ha et al. have already reported that this species produces erinacines A–C, which belong to the class of cyathane diterpenes, and a new sesquiterpenoid named dentipellin [12]. However, the authors did not provide any details on the provenance of the strain, which they obtained from a Korean culture collection without even reporting a strain designation number. Hence, the identity of this strain remains to be verified. This is because the taxonomy of the genus *Dentipellis* and many other resupinate Basidiomycota is in a dire need of revision using modern concepts. However, this is beyond the scope of the present study, in which we report the isolation and biological activities of several unprecedented and other known diterpenoids from a culture of *D. fragilis* that was obtained from a natural reserve in Southern Germany.

## 2. Results and Discussion

### 2.1. Identity of the Producer Strain

As described in the Materials and Methods, the strain was obtained from a specimen that had been collected in a natural habitat and identified from morphological characteristics of the basidiomes. Here, we have studied the mycelial culture for secondary metabolite production for the first time. A concurrent BLAST (Basic Local Alignment Tool, https://blast.ncbi.nlm.nih.gov/ (accessed on 3 August 2022)) search using the ITS rDNA sequence data showed over 99% homology with the only other available sequence of *D. fragilis* (GenBank accession number: AF506387), which was generated from a basidiome collected in the natural environment by Larsson and Larsson [13].

### 2.2. Structure Elucidation of Cyathane-Xyloside Compounds (***1***–***10***)

**Figure 1:**
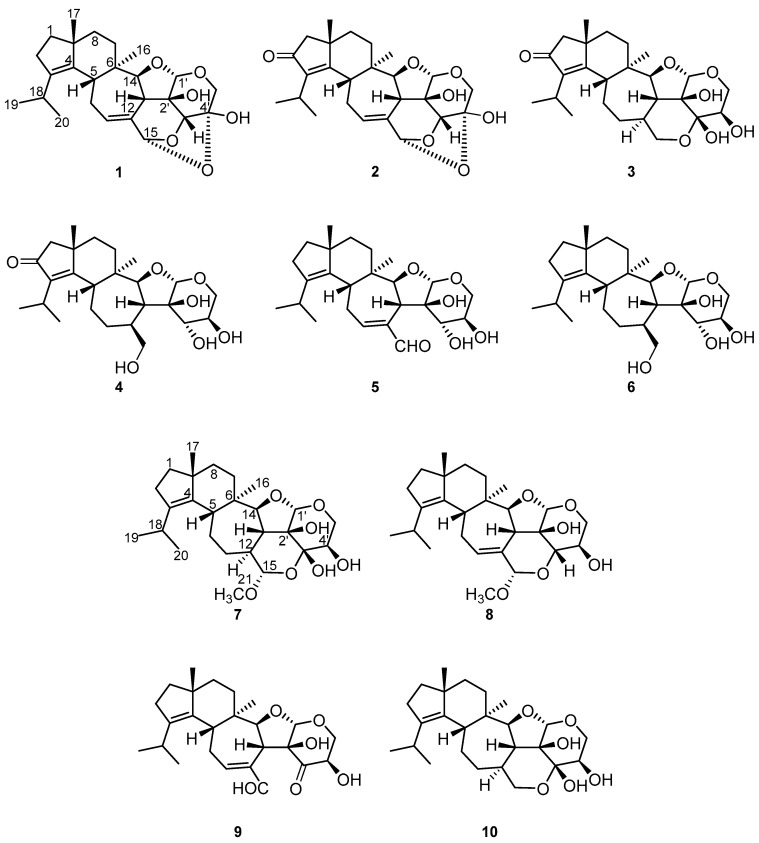
Structures of dentifragilins **A**–**H** (**1**–**8**) and of the known compounds striatal D (**9**) and laxitextine A (**10**), all isolated from submerged cultures of *D. fragilis*.

An exhaustive HPLC-DAD/MS analysis of ethyl acetate (EtOAc) crude extracts of *D. fragilis* indicated the presence of unprecedented secondary metabolites (Figure S3). Hence, we performed a large-scale fermentation and purification of the extracts, leading to the isolation of hitherto undescribed compounds **1**–**8**, named dentifragilins **A**–**H**, as well as the known metabolites striatal D (**9**) [14] and laxitextine A (**10**) [15] (Figure 1). The NMR data of the metabolites are given in Table 1, Table 2 and Table 3. Compounds **7** and **8** possess the basic carbon skeleton of striatins but with an α-orientation of the methoxy-group at C-15, as has been reported for striatoid A [16]. Because striatins have already been described as being formed spontaneously from the corresponding striatals upon treatment with methanol [14], they could be considered as artefacts. Thus, it remains questionable whether compounds **7** and **8** might also be transformation products from striatal-like compounds due to the isolation procedure.

**Figure 2:**
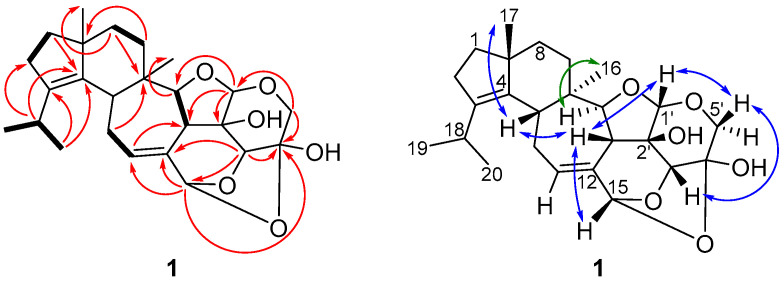
Key NMR correlations of dentifragilin A (**1**). Bold bonds: ^1^H,^1^H COSY correlations; red arrows: ^1^H,^13^C HMBC correlations; blue arrows: ^1^H,^1^H ROESY correlations, indicating β-orientation; and green arrows: ^1^H,^1^H ROESY, indicating α-orientation.

Compound **1** was obtained as a white solid in a yield of 3.2 mg. The HR-(+)ESIMS exhibited an [M + H]^+^ ion at *m*/*z* 431.2425, which is in agreement with the molecular formula C_25_H_34_O_6_ and nine degrees of unsaturation. The presence of 25 carbon atoms in the ^13^C NMR spectrum confirmed the molecular formula, whereas the ^1^H NMR spectrum exhibited 32 protons (Table 1). In the DEPT- (distortionless enhancement by polarization transfer) HSQC (heteronuclear single quantum coherence) spectrum, four methyl groups, six methylenes, and eight methines were present. According to the molecular formula and the DEPT-HSQC spectrum, seven carbons must be quaternary. Apart from four olefinic carbons (δc 139.8 ppm [C-3], δc 136.4 ppm [C-4], δc 127.4 ppm [C-11], δc 134.0 ppm [C-12]), no sp^2^ hybridized carbon was observed in the ^13^C NMR spectrum. Therefore, the remaining seven indices of hydrogen deficiency in the molecule implied that compound **1** consisted of a system containing seven rings. The basic carbon skeleton was deduced based on its COSY (correlation spectroscopy) and key HMBC correlations to be a cyathane-xyloside (Figure 2, left), closely related to laxitextine B [15]. A detailed comparison of the NMR spectroscopic data indicated that the only difference with laxitextine B was that compound **1** lacks the hydroxy group at C-3′. This was obvious due to the absent signal of the hemiacetal carbon that resonates in laxitextine B at δc 101.6 ppm and the presence of a methine group (δc 83.6 ppm and δ_H_ 4.48 ppm [C-3′]). The relative configuration of compound **1** was determined from the ROESY (rotating frame overhauser enhancement spectroscopy) spectrum. ROESY correlations between H-17/H-5, H-5/H-13, H-13/H-15, H-13/H-1′, H-1′/H-5_β_’, H-5_β_’/H-3′ and H-3′/H-13 indicated that these protons were located on the same plane. In accordance with the configuration of laxitextine B [15] they were assigned as β-orientated (Figure 2, right). Similarly, ROESY correlations between H-14/H-16 indicated α-orientation. The configurations of C-2′ and C-4′ were established based on biosynthetic thoughts and the general stereochemistry of D-xylose. From a biogenetic perspective, this stereochemical assignment is consistent with the recently published X-ray structure of cyathinin A [17], a compound with the same core structure as cyathane xyloside **1**. Thus, the absolute stereochemistry was finally assigned as 5*R,* 6*R*, 9*R*, 13*R,* 14*S*, 15*R*, 1′*S*, 2′*R*, 3′*R*, 4′*S* (Figure 1) and compound **1** was named dentifragilin A (**1**).

Compound **2** was isolated as a yellowish solid in a yield of 2.4 mg. The molecular formula was determined to be C_25_H_32_O_7_ based on the molecular ion at *m*/*z* 445.2222 [M + H]^+^ of the HR-(+)ESIMS spectrum, thus possessing ten degrees of unsaturation. Through detailed analysis of the NMR spectroscopic data (Table 1), compound **2** was assigned as a cyathane-xyloside similar to dentifragilin A (**1**). The main difference between both compounds was the presence of a carbonyl group at C-2 (δ_C_ 207.8 ppm) in cyathane-xyloside **2**. A ^3^*J* HMBC (heteronuclear multiple bond correlation) from H-18 (δ_H_ 2.80 ppm) along with a ^2^*J* HMBC correlation from H-1 (δ_H_ 2.20/2.24 ppm) to C-2, which verified the position of the carbonyl group and established the structure of compound **2** (Figure 1), named dentifragilin B (**2**). Based on biogenetic considerations [15,16,17,18] and on the analysis of the ROESY data (correlations between H-17/H-5, H-5/H-13, H-1′/H-3′, H-3′/H-15 and H-16/H-14), the relative configuration of dentifragilin B (**2**) was assumed to be the same as that of compound **1.**

Compound **3** was isolated as a yellowish solid in a yield of 2.6 mg. The HR-(+)ESIMS spectrum exhibited an [M + H]^+^ ion at *m*/*z* 449.2537, corresponding to the molecular formula C_25_H_36_O_7_ and eight degrees of unsaturation. The NMR spectroscopic data resembled those of dentifragilin B (**2**) (Table 2), although differences were determined in the chemical shift values of C-11 (δ_C_ 33.7 ppm), C-12 (δ_C_ 40.4 ppm), C-15 (δ_C_ 65.5 ppm), C-3′ (δ_C_ 95.5 ppm), and C-4′ (δ_C_ 70.4 ppm). According to the DEPT-HSQC spectrum, compound **3** carried two additional methines (CH-12; CH-4′) and two additional methylenes (CH_2_-11; CH_2_-15). According to the chemical shift values, the double bond between C-11 and C-12 was missing. Furthermore, COSY correlations between H-15 (δ_H_ 3.60/3.83 ppm) and H-12 (δ_H_ 1.88 ppm) as well as between H-5′ (δ_H_ 3.69/4.04 ppm) and H-4′ (δ_H_ 3.92 ppm) indicated that compound **3** lacks the oxygen bridge between C-15 and C-4′. Taking into account the low field shifted chemical shift value of C-3′ compared to that of compound **1** (δ_C_ 83.6 ppm) and that one oxygen is missing to resemble the molecular formula, a hydroxy group needed to be attached to C-3′. Thus, the structure of compound **3** was established (Figure 1) and named dentifragilin C (**3**). The configuration of cyathane **3** was assigned based on its ROESY data (correlations between H-17/H-5, H-5/H-13, H-16/H-14, H-14/H-12 and H-12/H-4′) together with biosynthetic considerations [15,16,17,18].

Compound **4** was obtained as a brownish solid in a yield of 11.5 mg. The molecular formula C_25_H_38_O_7_, deduced from the [M + H]^+^ ion at *m*/*z* 451.2687 of the HR-(+)ESIMS spectrum, indicated seven degrees of unsaturation and suggested similarities to dentifragilin C (**3**). Although the 2D NMR spectroscopic data were highly comparable to compound **3** (Table 2), cyathane-xyloside **4** lacks the bridging oxygen atom between C-15 (δ_C_ 66.1 ppm) and C-3′ (δ_C_ 69.2 ppm). Instead, compound **4** exhibited the presence of an exomethylene group (CH_2_-15) in the DEPT-HSQC spectrum, which was located adjacent to C-12 (δ_C_ 39.6 ppm), as indicated by COSY correlations between H-12 (δ_H_ 2.46 ppm) and H-15 (δ_H_ 3.56/4.17 ppm). Moreover, the DEPT-HSQC spectrum showed an additional methine proton at δ_H_ 4.28 ppm (H-3′). COSY correlations between H-3′ and H-4′ (δ_H_ 3.86 ppm) placed this group adjacent to C-4′, leading to the structure of compound **4** (Figure 1), which was named dentifragilin D (**4**). The relative configuration of cyathane **4** was assigned by the analysis of its ROESY data (correlations between H-17/H-5, H-5/H-13, H-13/H-1′, H-16/H-14, H-14/H-12). The configuration of the carbon atoms C-3′ and C-4′ could not be determined from the ROESY data with certainty. Thus, they were assigned based on the common stereochemistry of D-xylose and biogenetic considerations [19,20].

Compound **5** was isolated as a yellowish solid in a yield of 1.3 mg. The molecular formula was determined to be C_25_H_36_O_6_ based on the [M + Na]^+^ ion at *m*/*z* 455.2404 in the HR-(+)ESIMS spectrum, indicating eight degrees of unsaturation. The analysis of the NMR spectroscopic data suggested a cyathane skeleton similar to that of dentifragilin A (**1**). Differences were determined by the presence of an aldehyde functionality at C-15 (δc 197.1 ppm, δ_H_ 9.41 ppm) and the loss of the oxygen bridges connecting C-15 to C-3′ (δc 70.1 ppm) and C-4′ (δc 68.4 ppm). ^3^*J* HMBC correlations from H-11 (δ_H_ 7.08 ppm) and H-13 (δ_H_ 3.37ppm) to C-15 attached the aldehyde adjacent to C-12 (δc 143.4 ppm). COSY correlations between H-3′ (δ_H_ 3.90 ppm) and H-4′ (δ_H_ 3.56 ppm) corroborated the connection of these methine groups. In accordance with the molecular formula and their chemical shift values, C-3′ and C-4′ carried hydroxy groups. This finding completed the structure elucidation of compound **5**, which was named dentifragilin E. The relative configuration of compound **5** was ascertained to be the same as cyathane **4**, based on the analysis of its ROESY data (correlations between H-17/H-5, H-5/H-13, H-13/H-1′ and H-16/H-14) and the assumptions that were made for the elucidation of compound **4**.

Compound **6** was isolated as a white solid in a yield of 0.6 mg. The molecular formula was determined based on the [M + H]^+^ ion at *m*/*z* 437.2898 in the HR-(+)ESIMS spectrum to be C_25_H_40_O_6_, consistent with six degrees of unsaturation. The 1D and 2D NMR spectra were similar to those of dentifragilin D (**4**) (Table 2). The main difference between the two compounds was the absence of a carbonyl group at C-2 (δ_C_ 28.2 ppm) in cyathane **6**, which was in agreement with the degrees of unsaturation and a characteristic chemical shift value for this carbon. Furthermore, this structural assignment was corroborated due to the presence of an additional methylene signal (δ_H_ 2.28 ppm [H-2]) in the DEPT-HSQC spectrum. Key HMBC correlations from H-18 (δ_H_ 2.76 ppm) and H-1 (δ_H_ 1.55/1.63 ppm) to C-2 localized this group adjacent to the quaternary carbon C-3 (δ_C_ 138.5 ppm) and established the structure of compound **6** (Figure 1), named dentifragilin F (**6**). The relative configuration of compound **6** was ascertained to be the same as that of cyathane **4** based on the analysis of the ROESY data (key correlations between H-17/H-5, H-15/H-3′, H-3′/H-1′, H-1′/H-13, H-13/OH-2′, H-16/H-14, H-14/H-12, H-14/OH-3′, OH-3′/H-4′).

Compound **7** was isolated as a yellowish solid in a yield of 0.4 mg (Table 3). The molecular formula was assigned by HR-(+)ESIMS to be C_26_H_40_O_7_ based on the [M + Na]^+^ ion at *m*/*z* 487.2667, thus possessing seven degrees of unsaturation. Although compounds **7** and **3** exhibited similar chemical shift values together with comparable COSY and HMBC correlations, compound **7** differed by the presence of a methoxy group (OCH_3_-21) attached to C-15 (δ_C_ 104.6 ppm). This was confirmed by an additional low field shifted methyl singlet (δ_H_ 3.53 ppm; δ_C_ 56.5 ppm [CH_3_-21]) in the DEPT-HSQC spectrum, along with HMBC correlations from H-21 to C-15. Furthermore, compound **7** was lacking the carbonyl function at C-2 (δ_C_ 28.1 ppm) due to the presence of an additional methylene group (δ_H_ 2.28 ppm [H-2]) in the DEPT-HSQC spectrum. HMBC correlations from H-1 (δ_H_ 1.57/1.63 ppm [H-1]) and H-18 (δ_H_ 2.73 ppm [H-18]) to C-2 verified its position. Due to overlapping signals, the stereochemistry of C-15 could not be assigned with certainty from the analysis of the NMR data of compound **7**. However, a stereochemical assignment was possible from the analysis of the ROESY data of its congener, compound **8**, as a correlation between H-15 (δ_H_ 5.17 ppm) and H-13 (δ_H_ 2.89 ppm) indicated α-orientation of the methoxy group (OCH_3_-21) attached to C-15. Based on biogenetic considerations, it was assumed that the methoxy group in cyathane **7** should possess the same orientation. This finding is in good accordance with the stereochemistry of the structurally related striatoid A [16]. The further stereochemical assignment of compound **7** was ascertained to be similar to that of cyathane **3**, based on the analysis of the ROESY data (key correlations between H-17/H-5, H-5/H-13, H-1′/H-5_β_’, H-5_β_’/OH-3′, H-16/H-14, H-14/H-12, H-12/H-4′). The stereochemical assignment of OH-2′ was based on biogenetic considerations [14,15,16,17,21]. Compound **7** was designated as dentifragilin G (**7**) (Figure 1).

Compound **8** was isolated as a white solid in a yield of 0.7 mg. The HR-(+)ESIMS spectrum exhibited an [M + Na]^+^ ion at *m*/*z* 469.2560 which was consistent with the molecular formula C_26_H_38_O_6_ and eight degrees of unsaturation. The interpretation of the 2D NMR spectra revealed that its core structure was the same as that of dentifragilin G (**7**). Significant differences with cyathane **7** were the presence of a double bond between C-11 (δ_C_ 132.0 ppm) and C-12 (δ_C_ 134.9 ppm) and a missing hydroxy group at C-3′ (δ_C_ 84.5 ppm). The position of the double bond was confirmed by HMBC correlations from H-11 (δ_H_ 6.01 ppm) to C-15 (δ_C_ 99.8 ppm) and from H-10 (δ_H_ 2.43 ppm) to C-12 as well as by COSY correlations between H-10 (δ_H_ 2.43/2.61 ppm) and H-11. C-3′ was identified as a methine group in the DEPT-HSQC spectrum, and its position was secured based on COSY correlations between H-3′ (δ_H_ 3.79 ppm) and H-4′ (δ_H_ 3.94 ppm). Thus, the constitution of compound **8** was established (Figure 1) and it was named dentifragilin H (**8**). The ROESY correlations of dentifragilin H (**8**) were comparable to those of compound **7** (key correlations between H-17/H-5, H-5/H-13, H-13/H-15, H-15/H-3′, H-3′/H-5_β_’, H-5_β_’/H-1′ and H-16/H-14), which together with biogenetic considerations [14,15,16,17,21] indicated the same stereochemical assignment for both cyathane-xylosides.

The NMR spectroscopic data of the known cyathane-xylosides **9**–**10** were in accordance with the literature [15,17], allowing the structural elucidation of these compounds.

### 2.3. Biological Activity of Compounds ***1***–***9***

The cyathane derivatives **1**–**9** exhibited diverse antimicrobial activities against bacteria (Table 4) and fungi (Table 5) as well as cytotoxic effects against a panel of mammalian cell lines (Table 6). Cyathane-xylosides isolated from species of the genera *Cyathus* and *Hericium*, namely striatins, striatals, and erinacines, have exhibited significant bioactivities [22]. These include antimicrobial activities (striatins and striatals) and kappa-opioid receptor agonist activities (erinacine E) as well as the stimulation of nerve growth factor (NGF). In our current study, striatal D (**9**) showed significant antimicrobial properties with minimum inhibitory concentrations (MICs) of 1.0–66.6 µg/mL against most tested strains, with MIC values against *Bacillus subtilis* (1.0 µg/mL) and *Rhodotorula glutinis* (1.0 µg/mL) stronger than the positive control. Similarly, the novel dentifragilin A (**1**) exhibited strong activities against *B. subtilis* (1.0 µg/mL) and *S. aureus* (4.2 µg/mL). However, only moderate MIC values were observed for compound **1** against fungi (*R. glutinis* and *M. hiemalis*, both with MIC values of 16.7 µg/mL). Dentifragilin D (**4**) showed moderate activities against the Gram-positive bacteria *B. subtilis* (16.7 µg/mL) and *S. aureus* (33.3 µg/mL). Dentifragilin E (**5**) exhibited moderate antibacterial activities against the same Gram-positive bacteria, with an MIC value of 16.7 µg/mL. Only weak activities could be observed for dentifragilin B (**2**) against Gram-positive bacteria (*B. subtilis* and *S. aureus*), with MIC values of 66.6 µg/mL. Dentifragilin C (**3**) was devoid of any activity against all tested microorganisms, whereas cyathanes **6**–**8** were not tested due to insufficient isolated amounts.

Cytotoxicity assays against mammalian cell lines revealed that compounds **1**, **8**, and **9** exhibited significant activities (Table 6). Notably, striatal D (**9**) showed the strongest cytotoxic activities against the cell lines SKOV-3 (ovarian carcinoma), A549 (squamous cell carcinoma), and MCF-7 (human breast adenocarcinoma) with half-maximal inhibitory concentrations (IC_50_) of 0.1 µM. All aforementioned compounds were active against the cell lines L929 (mouse fibroblasts) and KB3.1 (human endocervical adenocarcinoma). Compound **9** revealed the strongest cytotoxicity, with IC_50_ values of 0.8 µM (L929) and 0.4 µM (KB3.1), whereas compound **1** showed IC_50_ values of 5.8 µM (L929) and 2.2 µM (KB3.1) and compound **8** IC_50_ values of 10 µM (L929) and 2 µM (KB3.1). Dentifragilins **4**, **5**, and **7** showed only weak activities against these two cell lines (Table 6), whereas compounds **3** and **6** were not cytotoxic. Compound **2** had moderate cytotoxic effects only on KB3.1 at 51.0 µM, while no activity was seen against L929. Compounds **1** and **9** inhibited the growth of other cell lines, with IC_50_ values in the range of 0.7–15.8 µM and 0.1–1 µM, respectively (Table 6). Compounds **2**–**8** were not tested against the other cell lines due to insufficient isolated amounts.

## 3. Materials and Methods

### 3.1. General Experimental Procedures

HPLC-DAD/MS analysis for evaluating the crude extracts and the purity of isolated compounds was performed using an amaZon speed ETD ion trap mass spectrometer (Bruker Daltonics, Bremen, Germany) in positive and negative ionization modes. The HPLC system consisted of a Dionex UltiMate 3000 UHPLC (Thermo Fisher Scientific Inc., Waltham, MA, USA) equipped with a C18 Acquity UPLC BEH column (Waters, Milford, MA, USA) as stationary phase. Solvent A consisted of deionized H_2_O + 0.1% formic acid (FA) (*v*/*v*) and solvent B was acetonitrile (ACN) + 0.1% FA (*v*/*v*). The applied gradient was 5% B for 0.5 min, increasing to 100% B within 20 min and holding for 10 min at 100% B. The flow rate was 0.6 mL/min, and UV/Vis detection was set to 190–600 and 210 nm, respectively.

HR-(+)ESIMS data were recorded on a maXis ESI-TOF (Time of Flight) mass spectrometer (Bruker Daltonics) connected to an Agilent 1260 series HPLC-UV system (Agilent Technologies, Santa Clara, CA, USA) equipped with a C18 Acquity UPLC BEH column (Waters). Solvent A consisted of deionized H_2_O + 0.1% FA (*v*/*v*) and solvent B was ACN + 0.1% FA (*v*/*v*). The applied gradient was 5% B for 0.5 min, increasing within 19.5 min to 100% B and holding for 5 min at 100% B. The flow rate was set to 0.6 mL/min at 40 °C and UV/VIS detection to 200–600 nm. Molecular formulas were calculated using the Smart Formula algorithm of the Compass Data Analysis software (Bruker Daltonics, version 4.4).

The 1D and 2D NMR spectra were recorded on Avance III 500 (Bruker Biospin, Ettlingen, Germany, ^1^H: 500 MHz, ^13^C: 125 MHz) or Avance III 700 (Bruker Biospin, ^1^H: 700 MHz, ^13^C: 175 MHz) spectrometers. Chemical shifts were reported in parts per million (ppm) and coupling constants were calculated in Hertz (Hz). Deuterated solvents were used for the NMR measurements. The signal of the residual protons of the deuterated solvents was used as a reference for the calibration of ^1^H NMR chemical shifts, with a value of 7.27 ppm for CDCl_3_ and 3.31 ppm for CD_3_OD, respectively. ^13^C NMR chemical shifts were calibrated using the ^13^C signal of the deuterated solvents, with reference values of 77.00 ppm for CDCl_3_ and 49.15 ppm for CD_3_OD, respectively. HSQC spectra were recorded, multiplicity edited (DEPT-HSQC), and multiplicities of carbon signals were determined from these experiments. In the case that ^1^H NMR chemical shifts could not be determined directly from the ^1^H NMR spectra due to overlapping signals, the respective data were obtained from the HSQC spectrum.

UV/Vis spectra were recorded in methanol (MeOH) at a concentration of 0.02 mg/mL with a Shimadzu UV/VIS 2450 spectrophotometer (Kyoto, Japan).

Optical rotations were determined with an Anton Paar MCP-150 Polarimeter (Graz, Austria) with sodium D line at 589 nm and 100 mm path length at a concentration of 1.0 mg/mL using MeOH as the solvent.

All chemicals and solvents (analytical and HPLC grade) were obtained from AppliChem GmbH (Darmstadt, Germany), Carl Roth GmbH & Co. KG (Karlsruhe, Germany), Avantor Performance Materials (Deventer, Netherlands) and Merck (Darmstadt, Germany). Deionized water was prepared with a Purelab^®^ flex water purification system (Veolia Water Technologies, Celle, Germany).

### 3.2. Fungal Material

The specimen was collected on 1st August 2015 in the Bavarian Forest National Park (49.098387 N, 13.246003 E) on a dead beech (*Fagus sylvatica*) trunk. The mycelial culture was deposited at Deutsche Sammlung von Mikroorganismen und Zellkulturen (DSMZ) in Braunschweig under the designation number DSM 105465. The fungus was identified by morphological studies and sequencing of the ITS rDNA (5.8S gene region, the internal transcribed spacers ITS1 and ITS2) and LSU (large subunit) ribosome RNA genes, according to the well established procedure of Noumeur et al. [23]. The genomic DNA sequence was deposited to GenBank under the accession number MK463979.

### 3.3. Fermentation Scale-Up and Extraction of Metabolites

YMG (yeast and malt extract with glucose) medium for agar plates was prepared by dissolving glucose (4 g), yeast extract (4 g), malt extract (10 g) and agar (20 g) in 1 L of deionized water.

Fully-grown mycelial YMG agar plates of *D. fragilis* were used to inoculate ten plugs (5 mm diameter each) into eighteen 1 L Erlenmeyer culture flasks containing 400 mL liquid YMG medium (composition as reported above, except without agar and with pH adjusted to 6.3 before sterilization). These were incubated at 24 °C in the dark with a rotation of 160 rpm for 45 days, after which glucose was depleted. The depletion of glucose indicates the beginning of the stationary phase, which is accompanied by the production of secondary metabolites. Thus, the cultures were kept in the stationary phase for three additional days before they were combined, and secondary metabolites were extracted. The mycelia and supernatant were separated by filtration and extracted according to the protocol of Rupcic et al. [20]. Essentially, 1% (*m*/*v*) of Amberlite XAD^TM^ 16N adsorber resin (Sigma-Aldrich, Deisenhofen, Germany) was added to the supernatant and the resulting suspension was stirred over a period of 3 h. The resin was extracted with acetone and the solvent was evaporated under reduced pressure. The remaining aqueous layer was extracted three times with equal amounts of EtOAc. The combined organic layers were dried over anhydrous Na_2_SO_4_, filtered, and concentrated by evaporation to yield the supernatant crude extract (881 mg). The mycelial portion was extracted similarly except without using resin, yielding 367 mg of crude extract.

### 3.4. Isolation and Physico-Chemical Properties of Compounds

The mycelial and supernatant crude extracts obtained from the extraction of fungal cultures as described in 3.3 exhibited similar secondary metabolites according to HPLC-DAD/MS analyses, and were therefore combined. The resulting crude extract (1.25 g) was pre-fractionated using a Reveleris X2 (W.R. Grace and Co., Columbia, MD, USA) flash chromatography system with a 40 g silica gel-packed column (Reveleris^®^) as the stationary phase. The mobile phases were dichloromethane (CH_2_Cl_2_) as solvent A and CH_2_Cl_2_:MeOH (ratio 8:2) as solvent B. The flow rate was set to 60 mL/min and UV detection was performed at 190, 210, and 280 nm. The elution steps were as follows: 0% to 30% B within 30 min, isocratic conditions at 30% B for 2 min, 30% to 60% B within 15 min, isocratic conditions at 60% B for 2 min, and 60% to 100% B within 10 min. Fractions of 20 mL were collected. The intermediate products were divided into portions for further purification by preparative HPLC, as described in detail below; several runs were necessary to process the samples. Mass spectrometric screening (i.e., LC-MS analysis of the fractions that were collected from the preparative HPLC) was therefore carried out and the fractions containing the same compounds were finally combined.

Pre-fractionated extracts containing compounds **1**, **2**, **5**, **8**, **9**, or **10** were further purified by preparative HPLC (PLC 2020, Gilson, Middleton, WI, USA). Deionized H_2_O + 0.1% FA (*v*/*v*) (solvent A) and ACN + 0.1% FA (*v*/*v*) (solvent B) were used as the eluents. The stationary phase was a Synergi^TM^ 10 µm Polar-RP 80 Å (250 × 50 mm) AXIA™ packed column (Phenomenex Inc., Aschaffenburg, Germany). The flow rate was set to 40 mL/min and UV detection was performed at 190, 210, and 280 nm. The gradient was operated at 5% B for 15 min, from 5% to 40% B within 5 min, from 40% to 60% B within 30 min, from 60% to 100% B within 5 min, and at isocratic conditions at 100% B for 5 min. Fractions containing the same compounds based on mass spectrometric screening were combined to yield compounds **1** (*t*_R_ = 55 min), **2** (*t*_R_ = 37 min), **5** (*t*_R_ = 48 min), **8** (*t*_R_ = 44 min)**, 9** (*t*_R_ = 58 min), and **10** (*t*_R_ = 52 min). Compounds **6** (*t*_R_ = 43 min) and **7** (*t*_R_ = 45 min) were isolated as highly enriched mixtures in yields of 3.7 and 3.2 mg, respectively, but were not entirely pure. Pre-fractionated extracts containing compounds **3** and **4** were processed with the following gradient: 5% B for 10 min, 10% B for 5 min, from 10% to 65% B within 35 min, holding for 3 min at 65% B, from 65% to 75% B within 2 min, from 75% to 100% B within 5 min, and holding for 5 min at 100% B. The flow rate was set to 20 mL/min. Fractions containing the same compounds based on mass spectrometric screening were combined to yield compounds **3** (*t*_R_ = 36 min) and **4** (*t*_R_ = 33 min).

Compounds **6** and **7** were further purified on a semipreparative RP-HPLC using a Vanquish Core HPLC system (Thermo Fisher Scientific, Germering, Germany) equipped with a Synergi^TM^ 4 µm Hydro-RP (250 × 10 mm) column (Phenomenex Inc., Torrance, CA, USA). Solvent A consisted of deionized H_2_O + 0.1% FA (*v*/*v*) and solvent B was ACN + 0.1% FA (*v*/*v*). The flow rate was set to 4 mL/min and UV detection was performed at 220 nm. The elution gradient was operated at 5% B for 5 min, from 5% to 50% B within 5 min, from 50% to 65% B within 30 min, and from 65% to 100% B within 5 min to yield pure compounds **6** (*t*_R_ = 28 min) and **7** (*t*_R_ = 32 min). Compound **8** was isolated from a second fermentation that was carried out as described in the supplementary material. Compounds **1**, **3**, **4**, **5**, **7**, and **9** could be obtained from this fermentation as well.

Compound **1** (dentifragilin A): white solid; 3.2 mg; ${\left[\alpha \right]}_{D}^{20}$ = −96° (MeOH, 1.0 mg/mL); UV/VIS (MeOH): λ_max_ (log *ε*) = 203 (1.0) nm; NMR data (^1^H NMR: 500 MHz, ^13^C NMR: 125 MHz, CDCl_3_) see Table 1; HR-(+)ESIMS: *m*/*z* 413.2321 [M–H_2_O + H]^+^ (calcd. 413.2323 for C_25_H_33_O_5_^+^), 431.2425 [M + H]^+^ (calcd. 431.2428 for C_25_H_35_O_6_^+^), 453.2246 [M + Na]^+^ (calcd. 453.2248 for C_25_H_34_NaO_6_^+^), 883.4598 [2M + Na]^+^ (calcd. 883.4603 for C_50_H_68_NaO_12_^+^); *t*_R_ = 12.46 min (HR-LC-ESIMS). C_25_H_34_O_6_ (430.53 g/mol).

Compound **2** (dentifragilin B): yellowish solid; 2.4 mg; ${\left[\alpha \right]}_{D}^{20}$ = −82° (MeOH, 1.0 mg/mL); UV/VIS (MeOH): λ_max_ (log *ε*) = 240 (1.1) nm; NMR data (^1^H NMR: 500 MHz, ^13^C NMR: 125 MHz, CDCl_3_) see Table 1; HR-(+)ESIMS: *m*/*z* 445.2222 [M + H]^+^ (calcd. 445.2221 for C_25_H_33_O_7_^+^), 889.4371 [2M + H]^+^ (calcd. 889.4369 for C_50_H_65_O_14_^+^); *t*_R_ = 7.71 min (HR-LC-ESIMS). C_25_H_32_O_7_ (444.52 g/mol).

Compound **3** (dentifragilin C): yellowish solid; 2.6 mg; ${\left[\alpha \right]}_{D}^{20}$ = −70° (MeOH, 1.0 mg/mL); UV/VIS (MeOH): λ_max_ (log *ε*) = 242 (1.4) nm; NMR data (^1^H NMR: 700 MHz, ^13^C NMR: 175 MHz, CDCl_3_) see Table 1; HR-(+)ESIMS: *m*/*z* 449.2537 [M + H]^+^ (calcd. 449.2534 for C_25_H_37_O_7_^+^), 471.2354 [M + Na]^+^ (calcd. 471.2353 for C_25_H_36_NaO_7_^+^), 897.5004 [2M + H]^+^ (calcd. 897.4995 for C_50_H_73_O_14_^+^), 919.4819 [2M + Na]^+^ (calcd. 919.4814 for C_50_H_72_NaO_14_^+^); *t*_R_ = 7.98 min (HR-LC-ESIMS). C_25_H_36_O_7_ (448.55 g/mol).

Compound **4** (dentifragilin D): brownish solid; 11.5 mg; ${\left[\alpha \right]}_{D}^{20}$ = −17° (MeOH, 1.0 mg/mL); UV/VIS (MeOH): λ_max_ (log *ε*) = 211 (0.4), 237 (0.3) nm; NMR data (^1^H NMR: 500 MHz, ^13^C NMR: 125 MHz, CDCl_3_) see Table 2; HR-(+)ESIMS: *m*/*z* 451.2687 [M + H]^+^ (calcd. 451.2690 for C_25_H_39_O_7_^+^), 473.2509 [M + Na]^+^ (calcd. 473.2510 for C_25_H_38_NaO_7_^+^), 901.5317 [2M + H]^+^ (calcd. 901.5308 for C_50_H_77_O_14_^+^), 923.5134 [2M + Na]^+^ (calcd. 923.5127 for C_50_H_76_NaO_14_^+^); *t*_R_ = 7.22 min (HR-LC-ESIMS). C_25_H_38_O_7_ (450.56 g/mol).

Compound **5** (dentifragilin E): yellowish solid; 1.3 mg; ${\left[\alpha \right]}_{D}^{20}$ = −111° (MeOH, 1.0 mg/mL); UV/VIS (MeOH): λ_max_ (log *ε*) = 205 (0.7) nm; NMR data (^1^H: 500 MHz, ^13^C NMR: 125 MHz, CDCl_3_) see Table 2; HR-(+)ESIMS: *m*/*z* 415.2487 [M–H_2_O + H]^+^ (calcd. 415.2479 for C_25_H_35_O_5_^+^), 455.2404 [M + Na]^+^ (calcd. 455.2404 for C_25_H_36_NaO_6_^+^), 887.4920 [2M + Na]^+^ (calcd. 887.4916 for C_50_H_72_NaO_12_^+^); *t*_R_ = 11.45 min (HR-LC-ESIMS). C_25_H_36_O_6_ (432.55 g/mol).

Compound **6** (dentifragilin F): white solid; 0.6 mg; ${\left[\alpha \right]}_{D}^{20}$ = −10° (MeOH, 1.0 mg/mL); UV/VIS (MeOH): λ_max_ (log *ε*) = 213 (0.2) nm; NMR data (^1^H NMR: 500 MHz, ^13^C NMR: 125 MHz, CDCl_3_) see Table 2; HR-(+)ESIMS: *m*/*z* 419.2790 [M–H_2_O + H]^+^ (calcd. 419.2792 for C_25_H_39_O_5_^+^), 437.2898 [M + H]^+^ (calcd. 437.2898 for C_25_H_41_O_6_^+^), 459.2713 [M + Na]^+^ (calcd. 459.2717 for C_25_H_40_NaO_6_^+^), 873.5724 [2M + H]^+^ (calcd. 873.5723 for C_50_H_81_O_12_^+^), 895.5541 [2M + Na]^+^ (calcd. 895.5542 for C_50_H_80_NaO_12_^+^); *t*_R_ = 11.83 min (HR-LC-ESIMS). C_25_H_40_O_6_ (436.58 g/mol).

Compound **7** (dentifragilin G): yellowish solid; 0.4 mg; ${\left[\alpha \right]}_{D}^{20}$ = −20° (MeOH, 1.0 mg/mL); UV/VIS (MeOH): λ_max_ (log *ε*) = 205 (0.9) nm; NMR data (^1^H NMR: 700 MHz, ^13^C NMR: 175 MHz, CDCl_3_) see Table 3; HR-(+)ESIMS: *m*/*z* 447.2741 [M–H_2_O + H]^+^ (calcd. 447.2741 for C_26_H_39_O_6_^+^), 487.2667 [M + Na]^+^ (calcd. 487.2666 for C_26_H_40_NaO_7_^+^), 951.5448 [2M + Na]^+^ (calcd. 951.5440 for C_52_H_80_NaO_14_^+^); *t*_R_ = 12.95 min (HR-LC-ESIMS). C_26_H_40_O_7_ (464.59 g/mol).

Compound **8** (dentifragilin H): white solid; 0.3 mg; ${\left[\alpha \right]}_{D}^{20}$ = −56° (MeOH, 1.0 mg/mL); UV/VIS (MeOH): λ_max_ (log *ε*) = 206 (0.8) nm; NMR data (^1^H NMR: 700 MHz, ^13^C NMR: 175 MHz, CD_3_OD) see Table 3; HR-(+)ESIMS: *m*/*z* 415.2477 [M-OCH_3_ + H]^+^ (calcd. 415.2479 for C_25_H_35_O_5_^+^), 469.2560 [M + Na]^+^ (calcd. 469.2561 for C_26_H_38_NaO_6_^+^), 915.5228 [2M + Na]^+^ (calcd. 915.5229 for C_52_H_76_NaO_12_^+^); *t*_R_ = 11.94 min (HR-LC-ESIMS). C_26_H_38_O_6_ (446.57 g/mol).

Compound **9** (striatal D): white solid; 1.9 mg; ${\left[\alpha \right]}_{D}^{20}$ = −152° (MeOH, 1.0 mg/mL); UV/VIS (MeOH): λ_max_ (log*ε*) = 204 (0.9), 234 (0.9) nm; ^1^H NMR (500 MHz, CDCl_3_): δ = 1.00 (d, *J* = 6.9 Hz, 3H, H-20), 1.02 (d, *J* = 6.9 Hz, 3H, H-19), 1.03 (s, 3H, H-16), 1.04 (s, 3H, H-17), 1.57 (m, 1H, H-8), 1.59 (m, 1H, H-1), 1.59 (m, 1H, H-7), 1.63 (m, 1H, H-8), 1.66 (m, 1H, H-1), 1.66 (m, 1H, H-7), 2.32 (m, 2H, H-2), 2.43 (br d, *J* = 11.4 Hz, 1H, H-5), 2.76 (spt, *J* = 6.9 Hz, 1H, H-18), 2.78 (m, 1H, H-10), 2.88 (dddd, *J* = 18.9, 11.4, 4.4, 2.4 Hz, 1H, H-10), 3.37 (ddt, *J* = 10.6, 4.4, 2.1 Hz, 1H, H-13), 3.70 (dd, *J* = 11.8, 5.4 Hz, 1H, H-5′), 3.94 (d, *J* = 8.8 Hz, 1H, OH-4′), 4.01 (m, 1H, H-4′), 4.11 (d, *J* = 10.6 Hz, 1H, H-14), 4.25 (dd, *J* = 11.8, 4.0 Hz, 1H, H-5′), 5.24 (s, 1H, H-1′), 6.13 (s, 1H, OH-2′), 7.01 (dt, *J* = 8.2, 2.4 Hz, 1H, H-11), 9.29 (s, 1H, H-15) ppm. ^13^C{^1^H} NMR (125 MHz, CDCl_3_): δ = 17.3 (CH_3_-16), 21.5 (CH_3_-19), 21.9 (CH_3_-20), 24.6 (CH_3_-17), 26.9 (CH_2_-7), 27.2 (CH-18), 28.4 (CH_2_-2), 29.5 (CH_2_-10), 36.3 (CH_2_-8), 38.3 (CH_2_-1), 41.7 (C-6), 42.7 (CH-5), 45.8 (CH-13), 49.6 (C-9), 68.6 (CH_2_-5′), 74.9 (CH-4′), 83.6 (C-2′), 87.3 (CH-14), 108.3 (CH-1′), 136.0 (C-4), 140.1 (C-3), 142.2 (C-12), 159.3 (C-11), 196.2 (C-15). 204.2 (C-3′) ppm; HR-(+)ESIMS: *m*/*z* 413.2315 [M–H_2_O + H]^+^ (calcd. 413.2323 for C_25_H_33_O_5_^+^), 431.2420 [M + H]^+^ (calcd. 431.2428 for C_25_H_35_O_6_^+^), 453.2240 [M + Na]^+^ (calcd. 453.2248 for C_25_H_34_NaO_6_^+^), 883.4597 [2M + Na]^+^ (calcd. 883.4603 for C_50_H_68_NaO_12_^+^); *t*_R_ = 13.23 min (HR-LC-ESIMS). The spectroscopic data were in accordance with the literature [17]. C_25_H_34_O_6_ (430.53 g/mol).

Compound **10** (laxitextine A): brown solid; 1.7 mg; ${\left[\alpha \right]}_{D}^{20}$ = −10° (MeOH, 1.0 mg/mL); UV/VIS (MeOH): λ_max_ (log*ε*) = 206 (0.9); ^1^H NMR (500 MHz, CD_3_OD): δ = 0.93 (m, 1H, H-11), 0.96 (d, *J* = 6.8 Hz, 3H, H-20), 0.98 (s, 3H, H-16), 0.99 (d, *J* = 6.8 Hz, 3H, H-19), 1.10 (s, 3H, H-17), 1.44 (m, 1H, H-7), 1.47 (m, 1H, H-8), 1.57 (m, 1H, H-8), 1.59 (m, 1H, H-1), 1.62 (m, 1H, H-7), 1.64 (m, 1H, H-1), 1.76 (m, 1H, H-10), 1.82 (m, 1H, H-12), 1.84 (m, 1H, H-11), 1.93 (m, 1H, H-10), 2.08 (dd, *J* = 11.4, 8.0 Hz, 1H, H-13), 2.31 (m, 2H, H-2), 2.40 (br d, *J* = 9.5 Hz, 1H, H-5), 2.81 (spt, *J* = 6.8 Hz, 1H, H-18), 3.50 (t, *J* = 10.9 Hz, 1H, H-15), 3.58 (dd, *J* = 11.1, 5.7 Hz, 1H, H-5′), 3.78 (dd, *J* = 10.9, 5.1 Hz, 1H, H-15), 3.81 (dd, *J* = 5.7, 4.9, 1H, H-4′), 3.89 (dd, *J* = 11.1, 4.9 Hz, 1H, H-5′), 4.00 (d, *J* = 8.0 Hz, 1H, H-14), 5.04 (s, 1H, H-1′) ppm. ^13^C{^1^H} NMR (125 MHz, CD_3_OD): δ = 19.8 (CH_3_-16), 21.8 (CH_3_-19), 22.3 (CH_3_-20), 25.6 (CH_3_-17), 27.2 (CH_2_-10), 28.5 (CH-18), 29.1 (CH_2_-2), 29.5 (CH_2_-7), 35.7 (CH_2_-11), 38.0 (CH_2_-8), 39.6 (CH_2_-1), 41.9 (C-6), 42.1 (CH-12), 48.8 (CH-5), 49.2 (CH-13), 50.9 (C-9), 66.7 (CH_2_-15), 66.8 (CH_2_-5′), 71.1 (CH-4′), 79.2 (C-2′), 95.8 (CH-14), 97.3 (C-3′), 107.4 (CH-1′), 139.5 (CH-3), 140.7 (C-4) ppm. The chemical shift values of some overlapping carbon signals were determined from the DEPT-HSQC spectrum; HR-(+)ESIMS: *m*/*z* 457.2561 [M + Na]^+^ (calcd. 457.2561 for C_25_H_38_NaO_6_^+^), 891.5233 [2M + Na]^+^ (calcd. 891.5229 for C_50_H_76_NaO_12_^+^); *t*_R_ = 12.51 min (HR-LC-ESIMS). The spectroscopic data were in accordance with the literature [15]. C_25_H_38_O_6_ (434.56 g/mol).

### 3.5. Antimicrobial Assays

Serial dilution assays in 96-well microtiter plates were performed according to our standard protocol [24] in order to determine the minimum inhibitory concentrations (MICs) of pure isolated compounds against different microorganisms. Essentially, aliquots of compounds **1**–**5** and **9** (20 µL), each with an initial concentration of 1 mg/mL, were diluted in MeOH together with the respective microorganisms to concentrations ranging between 67 µg/mL and 0.5 µg/mL in the 96-well plates, followed by overnight incubation. The MIC was then determined as the lowest concentration at which no growth of the test organism was observed. A broad spectrum of clinically approved test pathogens and sensitivity indicator strains were used in the assays (Table 4 and Table 5). Ciprofloxacin, oxytetracycline, kanamycin, and gentamycin were used as positive controls against bacterial pathogens, while nystatin was used as the positive control against fungi.

### 3.6. Cytotoxicity Tests

A panel of mammalian cell lines was used to determine the cytotoxicity of the compounds following a previously published protocol [24]. The assay was initially performed using the sensitive cell lines KB3.1 (human endocervical adenocarcinoma) and L929 (mouse fibroblasts). Active compounds were further tested using the cell lines MCF-7 (human breast adenocarcinoma), PC-3 (human prostate adenocarcinoma), SKOV-3 (ovarian carcinoma), A549 (human lung carcinoma), and A431 (human squamous cell carcinomas) (Table 6). The cell lines A549, KB 3.1, and L929 were cultured in DMEM (Gibco, provided by Thermo Fisher Scientific), PC-3 in F12-K (Gibco) and MC-7, and SKOV-3 and A431 in RPMI (Gibco). With the exception of A549, all cell lines were supplemented with 10% bovine serum (Gibco). All cell lines were cultured at 37 °C. The L929 and KB 3.1 cell lines were cultivated under an atmosphere of 10% (*v*/*v*) carbon dioxide. Cytotoxicity tests were then carried out using MTT (3-(4,5-dimethylthiazol-2-yl)-2,5-diphenyltetrazolium bromide) in 96-well microtiter plates. Serial dilutions of the compounds (60 µL aliquots, each with an initial concentration of 1 mg/mL) were added into 120 µL aliquots of cell suspension (50000 cells/mL) in the 96-well plates. These were incubated for five days, after which an MTT assay was performed. The absorbance was taken at 590 nm using an Elisa plate reader (Victor, Überlingen, Germany). The half-maximal inhibitory concentration (IC_50_), the concentration at which cell growth inhibition was 50% of the control, was calculated as described elsewhere [24].

## 4. Conclusions

Our current study reveals that the Basidiomycota phylum remains a valuable source of novel anti-infectives. Eight previously undescribed cyathane-xylosides, compounds **1**–**8**, were isolated from submerged cultures of *D. fragilis* and structurally elucidated. Seven of these xylosides (**1**, **2**, **4**, **5**, **7**, and **8**) proved to be potent against various microbial pathogens and/or are interesting targets concerning cancer treatment. Therefore, these compounds should be provided in larger titers and screened in detail for other untested bioactivities. In particular, it will be interesting to study the effects of the novel compounds on NGF production in mammalian cells using state of the art methodology. Hitherto, many cyathane derivatives have proven to be NGF growth stimulators [19,25,26,27].

From the chemical structures and previous results on the biological activities of other cyathanes reported in the literature [22], it was further confirmed that those derivatives of the class that bear α,β-unsaturated carbonyl moieties, such as striatal D (**9**), possess broad spectrum activities in biological systems, where they presumably act as Michael acceptors. The same applies in principle for the second most active compound **1**, the second most active compound, as the acetale at C-15 might act as a kind of protecting group to form the corresponding α,β-unsaturated aldehyde in situ. This explains fairly well why these compounds showed the highest activity of all isolated cyathanes in our bioassays. The striatals were first reported from *Cyathus* species [21,28], which belong to the family Nidulariaceae (which may actually deserve to be recognized as an own order of the Agaricomycetes [29]). The genus *Dentipellis*, however, belongs to the Hericiaceae (order Russulales). The fact that similar compounds reported herein have been isolated from the related genera *Hericium* and *Laxitextum* further confirms the chemotaxonomic relationships among the latter family, as discussed previously [3,15]. Cyathanes have otherwise only been found in basidiomes of the genera *Sarcodon* and *Phellodon*, which belongs to yet another order and family of Basidiomycota, the Thelephoraceae [3,30], which are ectomycorrhizal symbionts that cannot easily be grown in culture.

## Figures and Tables

**Table 1:** 1D and 2D NMR spectroscopic data of compounds **1**–**3**.

No.	1 ^a^		2 ^a^		3 ^b^	
	δ_C_	δ_H_ (mult., *J* in Hz)	δ_C_	δ_H_ (mult., *J* in Hz)	δ_C_	δ_H_ (mult., *J* in Hz)
1	38.5, CH_2_	1.56 (m) 1.65 (m)	52.2, CH_2_	2.20 (d, 18.9) 2.24 (d, 18.9)	52.4, CH_2_	2.20 (d, 18.9) 2.23 (d, 18.9)
2	28.4, CH_2_	2.29 (m)	207.8, C		208.1, C	
3	139.8, C		143.6, C		142.7, C	
4	136.4, C		174.7, C		177.6, C	
5	42.5, CH	2.61 (m)	43.8, CH	3.00 (d, 11.9)	48.6, CH	2.70 (d, 9.3)
6	40.3, C		42.3, C		42.3, C	
7	27.1, CH_2_	1.52 (m)	26.3, CH_2_	1.58 (m) 1.64 (m)	27.4, CH_2_	1.62 (m)
8	36.4, CH_2_	1.55 (m)	37.3, CH_2_	α: 1.57 (m) β: 1.89 (dd, 11.5, 4.4)	38.0, CH_2_	1.56 (td, 13.4, 5.1) 1.87 (m)
9	49.7, C		42.5, C		42.4, C	
10	29.5, CH_2_	2.56 (m) 2.61 (m)	28.8, CH_2_	2.55 (dd, 17.9, 6.6) 2.77 (m)	25.3, CH_2_	1.86 (m) 1.97 (m)
11	127.4, CH	5.60 (m)	126.2, CH	5.62 (m)	33.7, CH_2_	0.98 (dd, 12.2, 1.6) 1.90 (m)
12	134.0, C		134.7, C		40.4, CH	1.88 (m)
13	43.5, CH	2.81 (m)	43.7, CH	2.83 (m)	48.0, CH	2.07 (dd, 11.2, 8.2)
14	92.3, CH	4.20 (d, 8.7)	91.5, CH	4.27 (d, 8.7)	93.5, CH	4.09 (d, 8.2)
15	103.2, CH	5.88 (s)	102.9, CH	5.90 (s)	65.5, CH_2_	3.60 (t, 10.9) 3.83 (dd, 10.9, 4.6)
16	17.0, CH_3_	1.02 (s)	16.8, CH_3_	1.05 (s)	18.9, CH_3_	1.03 (s)
17	24.6, CH_3_	1.05 (s)	24.4, CH_3_	1.24 (s)	24.5, CH_3_	1.27 (s)
18	27.0, CH	2.80 (spt, 6.7)	25.8, CH	2.80 (spt, 6.9)	25.9, CH	2.74 (spt, 6.9)
19	21.6, CH_3_	0.99 (d, 6.7) *	19.8, CH_3_	1.22 (d, 6.9)	19.6, CH_3_	1.22 (d, 6.9)
20	21.8, CH_3_	0.97 (d, 6.7) *	20.8, CH_3_	1.21 (d, 6.9)	20.8, CH_3_	1.19(d, 6.9)
1′	105.0, CH	5.03 (s)	105.0, CH	5.06 (s)	106.3, CH	5.12 (s)
2′	76.3, C		76.3, C		77.9, C	
3′	83.6, CH	4.48 (s)	83.7, CH	4.50 (s)	95.5, C	
4′	96,8, C		96.8, C		70.4, CH	3.92 (t, 5.3)
5′	63.3, CH_2_	β: 3.71 (d, 13.0) α: 3.98 (d, 13.0)	63.4, CH_2_	β: 3.72 (d, 13.1) α: 4.00 (d, 13.1)	65.0, CH_2_	β: 3.69 (dd, 11.7, 5.5) α: 4.04 (dd, 11.7, 5.1)
2′-OH						^+^
3′-OH						4.78 (br s)
4′-OH						^+^

^a^ recorded at 500 MHz (^1^H) or 125 MHz (^13^C), CDCl_3_, 298 K, δ_H_ and δ_C_ in ppm; ^b^ recorded at 700 MHz (^1^H) or 175 MHz (^13^C), CDCl_3_, 298 K, δ_H_ and δ_C_ in ppm; * assignments may be interchanged; ^+^ one hydroxy group was identified at 2.94 ppm, but could not be assigned with certainty.

**Table 2:** 1D and 2D NMR spectroscopic data of compounds **4**–**6**.

No.	4 ^b^		5 ^a^		6 ^a^	
	δ_C_	δ_H_ (mult., *J* in Hz)	δ_C_	δ_H_ (mult., *J* in Hz)	δ_C_	δ_H_ (mult., *J* in Hz)
1	52.3, CH_2_	2.18 (d, 18.7) 2.23 (d, 18.7)	38.3, CH_2_	1.58 (m) 1.65 (m)	38.3, CH_2_	1.55 (m) 1.63 (m)
2	208.1, C		28.4, CH_2_	2.30 (m)	28.2, CH_2_	2.28 (m)
3	142.8, C		139.7, C		138.5, C	
4	177.8, C		136.3, C		138.3, C	
5	47.2, CH	2.56 (d, 9.0)	42.5, CH	2.42 (d, 11.6)	45.4, CH	2.18 (m)
6	45.1, C		42.5, C		43.3, C	
7	27.3, CH_2_	1.69 (m)	26.6, CH_2_	1.64 (m)	28.0, CH_2_	1.59 (m)
8	37.9, CH_2_	1.54 (m) 1.88 (m)	36.4, CH_2_	1.56 (m)	36.7, CH_2_	1.53 (m)
9	42.1, C		49.6, C		49.3, C	
10	24.4, CH_2_	1.80 (m) 1.86 (m)	29.6, CH_2_	2.76 (m) 2.87 (m)	24.8, CH_2_	1.84 (m) 1.67 (m)
11	35.6, CH_2_	1.45 (m) 1.92 (m)	161.3, CH	7.08 (m)	35.3, CH_2_	1.41 (m) 1.86 (m)
12	39.6, CH	2.46 (m)	143.4, C		39.4, CH	2.42 (m)
13	46.7, CH	2.47 (m)	46.6, CH	3.37 (m)	46.9, CH	2.45 (dd, 9.2, 10.7)
14	86.0, CH	3.98 (d, 8.8)	85.8, CH	4.19 (d, 10.5)	86.7, CH	3.92 (d, 9.2)
15	66.1, CH_2_	3.56 (dd, 11.7, 3.1) 4.17 (dd, 11.7, 3.1)	197.1, C	9.41 (s)	66.6, CH_2_	4.11 (dd, 11.0, 1.8) 3.55 (br d, 11.0)
16	18.7, CH_3_	0.97 (s)	17.3, CH_3_	1.00 (s)	18.8, CH_3_	0.93 (s)
17	24.5, CH_3_	1.28 (s)	24.6, CH_3_	1.05 (s)	24.9, CH_3_	1.08 (s)
18	25.8, CH	2.75 (spt, 6.9)	27.1, CH	2.75 (m)	27.0, CH	2.76 (spt, 6.8)
19	19.5, CH_3_	1.21 (d, 6.9)	21.5, CH_3_	1.01 (6.9)	21.4, CH_3_	0.96 (d, 6.8)
20	20.8, CH_3_	1.18 (d, 6.9)	21.9, CH_3_	0.99 (6.9)	21.8, CH_3_	0.95 (d, 6.8)
1′	102.2, CH	5.05 (s)	102.0, CH	5.16 (s)	102.2, CH	5.04 (br d, 1.1)
2′	73.8, C		74.0, C		73.7, C	
3′	69.2, CH	4.28 (m)	70.1, CH	3.90 (br s)	69.3, CH	4.24 (m)
4′	68.7, CH	3.86 (m)	68.4, CH	3.56 (m)	68.8, CH	3.83 (m)
5′	59.7, CH_2_	β: 3.73 (br d, 12.9) α: 4.28 (br d, 12.9)	59.8, CH_2_	3.66 (d, 11.8) * 4.22 (d, 11.8) *	59.8, CH_2_	3.72 (d, 12.9) * 4.26 (d, 12.9) *
15-OH						2.90 (br s)
2′-OH				5.65 (br s)		5.00 (s)
3′-OH				1.79 (br s)		2.54 (d, 10.7)
4′-OH				3.76 (m)		3.43 (br d, 6.4)

^a^ recorded at 500 MHz (^1^H) or 125 MHz (^13^C), CDCl_3_, 298 K, δ_H_ and δ_C_ in ppm; ^b^ recorded at 700 MHz (^1^H) or 175 MHz (^13^C), CDCl_3_, 298 K, δ_H_ and δ_C_ in ppm. * an assignment of the α- and β-position was not possible.

**Table 3:** 1D and 2D NMR spectroscopic data of compounds **7**–**8**.

No.	7 ^b^		8 ^c^	
	δ_C_	δ_H_ (mult., *J* in Hz)	δ_C_	δ_H_ (mult., *J* in Hz)
1	38.3, CH_2_	1.57 (m) 1.63 (m)	39.7, CH_2_	1.58 (m) 1.65 (m)
2	28.1, CH_2_	2.28 (m)	29.2, CH_2_	2.32 (m)
3	138.5, C		140.4, C	
4	138.4, C		139.0, C	
5	47.1, CH	2.27 (m)	45.1, CH	2.55 (d, 11.8 Hz))
6	40.3, C		41.5, C	
7	28.3, CH_2_	1.54 (m)	28.4, CH_2_	1.46 (ddd, 13.5, 4.6, 1.8) 1.74 (td, 13.5, 4.6)
8	36.6, CH_2_	1.50 (m) 1.54 (m)	37.8, CH_2_	1.51 (ddd, 13.5, 4.6, 1.8) 1.58 (m)
9	49.5, C		50.9, C	
10	25.1, CH_2_	1.92 (dddd, 14.6, 4.5, 3.2) ^+^ 1.68 (m)	29.7, CH_2_	2.43 (m) 2.61 (m)
11	35.9, CH_2_	1.08 (m) 2.19 (m)	132.0, CH	6.01 (m)
12	43.5, CH	1.76 (m)	134.9, C	
13	46.0, CH	2.17 (m)	46.8, CH	2.89 (m)
14	93.3, CH	4.01 (d, 8.6)	92.4, CH	4.13 (d, 9.9)
15	104.6, CH	4.50 (d, 8.4)	99.8, CH	5.17 (m)
16	18.9, CH_3_	0.99 (s)	18.1, CH_3_	1.00 (s)
17	25.0, CH_3_	1.08 (s)	25.2, CH_3_	1.07 (s)
18	27.1, CH	2.73 (spt, 6.7)	28.4, CH	2.86 (spt, 6.8)
19	21.3, CH_3_	0.97 (t, 6.7)	22.0, CH_3_	1.01 (d, 6.8)
20	21.8, CH_3_	0.95 (t, 6.7)	22.4, CH_3_	0.99 (d, 6.8)
21	56.5, CH_3_	3.53 (s)	56.4, CH_3_	3.55 (s)
1′	106.5, CH	4.90 (s)	108.1, CH	4.97 (s)
2′	80.5, C		81.2, C	
3′	96.1, C		84.5, CH	3.79 (d, 9.8)
4′	69.8, CH	4.26 (dd, 10.7, 5.4)	68.4, CH	3.94 (td, 9.8, 5.0)
5′	63.0, CH_2_	β: 3.36 (dd, 11.6, 10.7) α: 3.83 (dd, 11.6, 5.4)	66.2, CH_2_	β: 3.13 (dd, 11.4, 9.8) α: 3.69 (dd, 11.4, 5.0)
2′-OH		3.16 (br s)		
3′-OH		4.72 (s)		
4′-OH		*		

^b^ recorded at 700 MHz (^1^H) or 175 MHz (^13^C), CDCl_3_, 298 K, δ_H_ and δ_C_ in ppm; ^c^ recorded at 700 MHz (^1^H) or 175 MHz (^13^C), CD_3_OD, 298 K, δ_H_ and δ_C_ in ppm; * could not be identified with certainty; ^+^ the fourth coupling constant could not be determined with certainty.

**Table 4:** Antibacterial activities of compounds **1**–**9**.

Microorganism	MIC (µg/mL)									
	1	2	3	4	5	6	7	8	9	Ref.
*Bacillus subtilis* (DSM 10)	1.0	66.6	n.i.	16.7	16.7	ND	ND	ND	1.0	4.2 ^a^
*Mycolicibacterium smegmatis* (ATCC 700084)	66.6	n.i.	n.i.	n.i.	n.i.	ND	ND	ND	33.3	1.7 ^b^
*Staphylococcus aureus* (DSM 346)	4.2	66.6	n.i.	33.3	16.7	ND	ND	ND	2.1	0.2 ^a^
*Actinobacter baumannii* (DSM 30008)	n.i.	n.i.	n.i.	n.i.	n.i.	ND	ND	ND	66.6	0.3 ^c^
*Chromobacterium violaceum* (DSM 30191)	n.i.	n.i.	n.i.	n.i.	66.6	ND	ND	ND	16.6	0.8 ^a^
*Escherichia coli* (DSM 1116)	n.i.	n.i.	n.i.	n.i.	n.i.	ND	ND	ND	66.6	3.3 ^a^
*Pseudomonas aeruginosa* (DSM PA14)	n.i.	n.i.	n.i.	n.i.	n.i.	ND	ND	ND	n.i.	0.4 ^d^

n.i.: no inhibition, ND: not tested, Ref.: reference (^a^: oxytetracycline, 2 mg/mL; ^b^: kanamycin 2 mg/mL; ^c^: ciprofloxacin, 2.5 mg/mL; ^d^: gentamycin, 2 mg/mL).

**Table 5:** Antifungal activities of compounds **1**–**9**.

Microorganism	MIC (µg/mL)									
	1	2	3	4	5	6	7	8	9	Ref.
*Candida albicans* (DSM 1665)	n.i.	n.i.	n.i.	n.i.	n.i.	ND	ND	ND	33.3	8.3
*Pichia anomala* (DSM 6766)	66.6	n.i.	n.i.	n.i.	n.i.	ND	ND	ND	33.3	8.3
*Rhodotorula glutinis* (DSM 10134)	16.7	n.i.	n.i.	66.6	66.6	ND	ND	ND	1.0	2.1
*Schizosaccharomyces pombe* (DSM 70572)	33.3	n.i.	n.i.	n.i.	n.i.	ND	ND	ND	4.2	4.2
*Mucor hiemalis* (DSM 2656)	16.7	n.i.	n.i.	66.6	33.3	ND	ND	ND	2.1	4.2

n.i.: no inhibition, ND: not tested, Ref.: reference (nystatin, 20 mg/mL).

**Table 6:** Cytotoxic activities of compounds **1**–**9**.

Cell line	IC_50_ (µM)									
	1	2	3	4	5	6	7	8	9	Ref.
L929 (ACC2)	5.8	n.c.	n.c.	48.9	34.7	n.c.	14.9	10.0	0.8	4.7 × 10^−5^
KB3.1 (ACC158)	2.2	51.0	n.c.	42.2	15.7	n.c.	10.3	2.0	0.4	3.3 × 10^−5^
MCF-7 (A115)	0.7	ND	ND	ND	ND	ND	ND	ND	0.1	3.0 × 10^−5^
A549 (ACC107)	2.8	ND	ND	ND	ND	ND	ND	ND	0.1	9.5 × 10^−5^
PC-3 (ACC465)	2.3	ND	ND	ND	ND	ND	ND	ND	0.4	1.5 × 10^−4^
SKOV-3	1.9	ND	ND	ND	ND	ND	ND	ND	0.1	5.1 × 10^−5^
A431 (ACC91)	15.8	ND	ND	ND	ND	ND	ND	ND	1.0	6.7 × 10^−5^

ND: not tested, n.c.: no cytotoxicity, Ref.: reference (epothilon B).

## Data Availability

All generated data are either available in the Supplementary Information or in the manuscript.

## Supplementary Materials

The following supporting information can be downloaded at: https://www.mdpi.com/article/10.3390/antibiotics11081072/s1, Figure S1: *D. fragilis* DSM 105465 growing on YMG agar plate. Table S1: Obtained ITS rDNA consensus sequence of *D. fragilis*. Table S2: List of reference taxa and their corresponding ITS rDNA sequences used for molecular phylogeny assessment. Figure S2: Phylogenetic tree, displaying the relationships of *D. fragilis* (highlighted in red) to selected species of the genera *Dentipellis*, *Hericium* and *Laxitextum* based on ITS ribosomal DNA (rDNA) sequences. Sequence alignment of the ITS rDNA was performed with ClustalW [31] and the tree was constructed using the neighbor-joining method [32] with 1000 Bootstrap replicates [33]. Evolutionary distances were computed with the Tamura-Nei model [34] and the rate variation among sites gamma distributed (gamma parameter = 1.00). GenBank accession numbers are given in parentheses. The scale bar represents 0.02 change per position. Analyses were done in MEGA 11 [35] (version 11.0.11) using default settings. Figure S3: HPLC-DAD chromatograms (detection at 190–600 nm) of the supernatant (YMS) and mycelial (YMM) ethyl acetate crude extracts of *D. fragilis* DSM 105465, cultured in YMG liquid media. Figure S4: HPLC-DAD chromatogram of compound **1**. Figure S5: HR-(+)ESIMS spectrum of compound **1**. Figure S6: ^1^H NMR spectrum (500 MHz, CDCl_3_, 298 K) of the purified compound **1**. W: H_2_O, EA: ethyl acetate. Figure S7: ^13^C NMR spectrum (125 MHz, CDCl_3_, 298 K) of the purified compound **1**. EA: ethyl acetate. Figure S8: ^1^H,^13^C HSQC-DEPT spectrum (500 MHz, CDCl_3_, 298 K) of the purified compound **1**. Figure S9: ^1^H,^1^H COSY spectrum (500 MHz, CDCl_3_, 298 K) of the purified compound **1**. Figure S10: ^1^H,^13^C HMBC spectrum (500 MHz, CDCl_3_, 298 K) of the purified compound **1**. Figure S11: ^1^H,^1^H ROESY spectrum (500 MHz, CDCl_3_, 298 K) of the purified compound **1**. Figure S12: UV/Vis spectrum of compound **1** in MeOH. Figure S13: HPLC-DAD chromatogram of compound **2**. Figure S14: HR-(+)ESIMS spectrum of compound **2**. Figure S15: ^1^H NMR spectrum (500 MHz, CDCl_3_, 298 K) of the purified compound **2**. W: H_2_O. Figure S16: ^13^C NMR spectrum (125 MHz, CDCl_3_, 298 K) of the purified compound **2**. Figure S17: ^1^H,^13^C HSQC-DEPT spectrum (500 MHz, CDCl_3_, 298 K) of the purified compound **2**. Figure S18: ^1^H,^13^C HMBC spectrum (500 MHz, CDCl_3_, 298 K) of the purified compound **2**. Figure S19: ^1^H,^1^H COSY spectrum (500 MHz, CDCl_3_, 298 K) of the purified compound **2**. Figure S20: ^1^H,^1^H ROESY spectrum (500 MHz, CDCl_3_, 298 K) of the purified compound **2**. Figure S21: UV/Vis spectrum of compound **2** in MeOH. Figure S22: HPLC-DAD chromatogram of compound **3**. Figure S23: HR-(+)ESIMS spectrum of compound **3**. Figure S24: ^1^H NMR spectrum (700 MHz, CDCl_3_, 298 K) of the purified compound **3**. EA: ethyl acetate, A: acetone. Figure S25: ^13^C NMR spectrum (175 MHz, CDCl_3_, 298 K) of the purified compound **3**. EA: ethyl acetate, A: acetone. Figure S26: ^1^H,^13^C HSQC-DEPT spectrum (700 MHz, CDCl_3_, 298 K) of the purified compound **3**. Figure S27: ^1^H,^13^C HMBC spectrum (700 MHz, CDCl_3_, 298 K) of the purified compound **3**. Figure S28: ^1^H,^1^H COSY spectrum (700 MHz, CDCl_3_, 298 K) of the purified compound **3**. Figure S29: ^1^H,^1^H ROESY spectrum (700 MHz, CDCl_3_, 298 K) of the purified compound **3**. Figure S30: UV/Vis spectrum of compound **3** in MeOH. Figure S31: HPLC-DAD chromatogram of compound **4**. Figure S32: HR-(+)ESIMS spectrum of compound **4**. Figure S33: ^1^H NMR spectrum (700 MHz, CDCl_3_, 298 K) of the purified compound **4**. EA: ethyl acetate. Figure S34: ^13^C NMR spectrum (175 MHz, CDCl_3_, 298 K) of the purified compound **4**. EA: ethyl acetate. Figure S35: ^1^H,^13^C HSQC-DEPT spectrum (700 MHz, CDCl_3_, 298 K) of the purified compound **4**. Figure S36: ^1^H,^13^C HMBC spectrum (700 MHz, CDCl_3_, 298 K) of the purified compound **4**. Figure S37: ^1^H,^1^H COSY spectrum (700 MHz, CDCl_3_, 298 K) of the purified compound **4**. Figure S38: ^1^H,^1^H ROESY spectrum (700 MHz, CDCl_3_, 298 K) of the purified compound **4**. Figure S39: UV/Vis spectrum of compound **4** in MeOH. Figure S40: HPLC-DAD chromatogram of compound **5**. Figure S41: HR-(+)ESIMS spectrum of compound **5**. Figure S42: ^1^H NMR spectrum (500 MHz, CDCl_3_, 298 K) of the purified compound **5**. EA: ethyl acetate. Figure S43: ^13^C NMR spectrum (125 MHz, CDCl_3_, 298 K) of the purified compound **5**. Figure S44: ^1^H,^13^C HSQC-DEPT spectrum (500 MHz, CDCl_3_, 298 K) of the purified compound **5**. Figure S45: ^1^H,^13^C HMBC spectrum (500 MHz, CDCl_3_, 298 K) of the purified compound **5**. Figure S46: ^1^H,^1^H COSY spectrum (500 MHz, CDCl_3_, 298 K) of the purified compound **5**. Figure S47: ^1^H,^1^H ROESY spectrum (500 MHz, CDCl_3_, 298 K) of the purified compound **5**. Figure S48: UV/Vis spectrum of compound **5** in MeOH. Figure S49: HPLC-DAD chromatogram of compound **6**. Figure S50: HR-(+)ESIMS spectrum of compound **6**. Figure S51: ^1^H NMR spectrum (500 MHz, CDCl_3_, 298 K) of the purified compound **6**. W: H_2_O, A: acetone, M: methanol. Figure S52: ^13^C NMR spectrum (125 MHz, CDCl_3_, 298 K) of the purified compound **6**. A: acetone, M: methanol. Figure S53: ^1^H,^13^C HSQC-DEPT spectrum (500 MHz, CDCl_3_, 298 K) of the purified compound **6**. Figure S54: ^1^H,^13^C HMBC spectrum (500 MHz, CDCl_3_, 298 K) of the purified compound **6**. Figure S55: ^1^H,^1^H COSY spectrum (500 MHz, CDCl_3_, 298 K) of the purified compound **6**. Figure S56: ^1^H,^1^H ROESY spectrum (500 MHz, CDCl_3_, 298 K) of the purified compound **6**. Figure S57: UV/Vis spectrum of compound **6** in MeOH. Figure S58: HPLC-DAD chromatogram of compound **7**. Figure S59: HR-(+)ESIMS spectrum of compound **7**. Figure S60: ^1^H NMR spectrum (700 MHz, CDCl_3_, 298 K) of the purified compound **7**. W: H_2_O, EA: ethyl acetate, A: acetone. Figure S61: ^13^C NMR spectrum (175 MHz, CDCl_3_, 298 K) of the purified compound **7**. EA: ethyl acetate, A: acetone. Figure S62: ^1^H,^13^C HSQC-DEPT spectrum (700 MHz, CDCl_3_, 298 K) of the purified compound **7**. Figure S63: ^1^H,^13^C HMBC spectrum (700 MHz, CDCl_3_, 298 K) of the purified compound **7**. Figure S64: ^1^H,^1^H COSY spectrum (700 MHz, CDCl_3_, 298 K) of the purified compound **7**. Figure S65: ^1^H,^1^H ROESY spectrum (700 MHz, CDCl_3_, 298 K) of the purified compound **7**. Figure S66: UV/Vis spectrum of compound **7** in MeOH. Figure S67: HPLC-DAD chromatogram of compound **8**. Figure S68: HR-(+)ESIMS spectrum of compound **8**. Figure S69: ^1^H NMR spectrum (700 MHz, CD_3_OD, 298 K) of the purified compound **8**. W: H_2_O, EA: ethyl acetate, A: acetone, M: methanol. Figure S70: ^13^C NMR spectrum (175 MHz, CD_3_OD, 298 K) of the purified compound **8**. EA: ethyl acetate, A: acetone. Figure S71: ^1^H,^13^C HSQC-DEPT spectrum (700 MHz, CD_3_OD, 298 K) of the purified compound **8**. Figure S72: ^1^H,^13^C HMBC spectrum (700 MHz, CD_3_OD, 298 K) of the purified compound **8**. Figure S73: ^1^H,^1^H COSY spectrum (700 MHz, CD_3_OD, 298 K) of the purified compound **8**. Figure S74: ^1^H,^1^H ROESY spectrum (700 MHz, CD_3_OD, 298 K) of the purified compound **8**. Figure S75: UV/Vis spectrum of compound **8** in MeOH. Figure S76: HPLC-DAD chromatogram of compound **9**. Figure S77: HR-(+)ESIMS spectrum of compound **9**. Figure S78: ^1^H NMR spectrum (500 MHz, CDCl_3_, 298 K) of the purified striatal D (**9**). W: H_2_O, EA: ethyl acetate. Figure S79: ^13^C NMR spectrum (125 MHz, CDCl_3_, 298 K) of the purified striatal D (**9**). EA: ethyl acetate. Figure S80: ^1^H,^13^C HSQC-DEPT spectrum (500 MHz, CDCl_3_, 298 K) of the purified striatal D (**9**). Figure S81: ^1^H,^13^C HMBC spectrum (500 MHz, CDCl_3_, 298 K) of the purified striatal D (**9**). Figure S82: ^1^H,^1^H COSY spectrum (500 MHz, CDCl_3_, 298 K) of the purified striatal D (**9**). Figure S83: ^1^H,^1^H ROESY spectrum (500 MHz, CDCl_3_, 298 K) of the purified striatal D (**9**). Figure S84: UV/Vis spectrum of compound **9** in MeOH. Figure S85: HPLC-DAD chromatogram of compound **10**. Figure S86: HR-(+)ESIMS spectrum of compound **10**. Figure S87: ^1^H NMR spectrum (500 MHz, CD_3_OD, 298 K) of the purified laxitextine A (**10**). W: H_2_O, M: methanol. Figure S88: ^13^C NMR spectrum (125 MHz, CD_3_OD, 298 K) of the purified laxitextine A (**10**). Figure S89: ^1^H,^13^C HSQC-DEPT spectrum (500 MHz, CD_3_OD, 298 K) of the purified laxitextine A (**10**). Figure S90: ^1^H,^13^C HMBC spectrum (500 MHz, CD_3_OD, 298 K) of the purified laxitextine A (**10**). Figure S91: ^1^H,^1^H COSY spectrum (500 MHz, CD_3_OD, 298 K) of the purified laxitextine A (**10**). Figure S92: UV/Vis spectrum of compound **10** in MeOH.
