# Supplementary material for: Antimicrobial and Cytotoxic Cyathane-Xylosides from Cultures of the Basidiomycete Dentipellis fragilis
Source: Antibiotics (Basel). 2022 Aug 8;11(8):1072. doi: 10.3390/antibiotics11081072 (PMC9405216; doi:10.3390/antibiotics11081072)
Supplement: Supplementary file 1 [file antibiotics-11-01072-s001.zip › antibiotics-1848837-supplementary.pdf]

Antimicrobial and Cytotoxic Cyathane-xylosides from Cultures of the Basidiomycete *Dentipellis fragilis*

Winnie Chemutai Sum[1, †], Nico Mitschke [1,2, †], Hedda Schrey[1], Kathrin Wittstein[1], Harald Kellner[3], Marc Stadler[1,4]* Josphat Clement Matasyoh[5,*]

1 Department Microbial Drugs, Helmholtz Centre for Infection Research GmbH (HZI), Inhoffenstraße 7, 38124 Braunschweig, Germany
2 Research Group for Marine Geochemistry (ICBM-MPI Bridging Group), Institute for Chemistry and Biology of the Marine Environment (ICBM), Carl von Ossietzky Universität Oldenburg, Carl-von-Ossietzky-Str. 9-11, 26129 Oldenburg, Germany
3 Department of Bio- and Environmental Sciences, Technische Universität Dresden - International Institute Zittau, Markt 23, 02763 Zittau, Germany
4 Institute of Microbiology, Technische Universität Braunschweig, Spielmannstraße 7, 38106 Braunschweig, Germany
5 Department of Chemistry, Egerton University, P.O. Box 536, 20115, Njoro, Kenya;

* Correspondence; Marc.Stadler@helmholtz-hzi.de; Tel.: +49-531-6181-4240
[†]W.C.S. and N.M. contributed equally to this paper.

Table of Contents

(1) *Dentipellis fragilis* (*D. fragilis*) growing on agar plate ... 3

(2) Phylogenetic relationships of *D. fragilis* ... 3

(3) Second fermentation and isolation of compound 8 ... 5

(4) HPLC-DAD chromatograms of crude extracts ... 6

(5) Analytical data of compound 1 ... 7

(6) Analytical data of compound 2 ... 12

(7) Analytical data of compound 3 ... 17

(8) Analytical data of compound 4 ... 22

(9) Analytical data of compound 5 ... 27

(10) Analytical data of compound 6 ... 32

(11) Analytical data of compound 7 ... 37

(12) Analytical data of compound 8 ... 42

(13) Analytical data of compound 9 ... 47

(14) Analytical data of compound 10 ... 52

References ... 57

(1) *Dentipellis fragilis* (*D. fragilis*) growing on agar plate

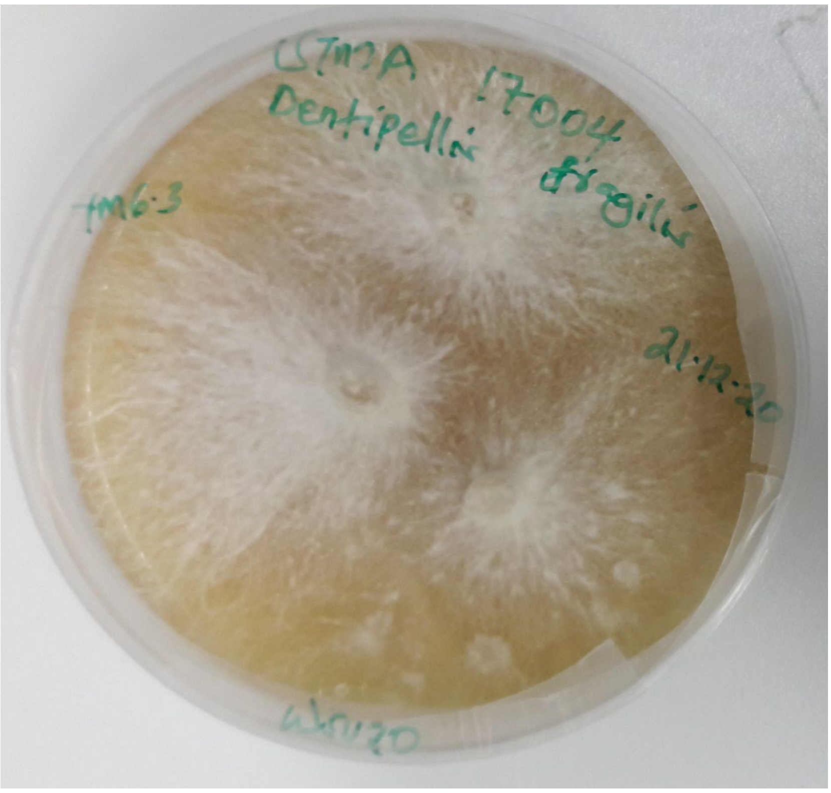

Figure S1: *D. fragilis* DSM 105465 growing on YMG agar plate.

(2) Phylogenetic relationships of *D. fragilis*

Table S1: Obtained ITS rDNA consensus sequence of *D. fragilis*.

D. fragilis (DSM 105465)
GAGGAAGTAAAAGTCGTAACAAGGTTTCCGTAGGTGAACCTGCGGAAGGATCATTACTG<br>AATTTGAAAGAGGTTGTTGCTGGCCTTCACCGGCATGTGCACGCCTTGATCTCATCCAT<br>CTTACACCTGTGCACCTTTGCGTGGGTCTGTTGGCTTTGCGGCCTTCAGACTTGCGTCT<br>TTTCATAAACTCTTATGTATGTAACAGAATGTCTAATGCTATAAAACGCATCTTATACAAC<br>TTTCAACAACGGATCTCTTGGCTCTCGCATCGATGAAGAACGCAGCGAAATGCGATAAG<br>TAATGTGAATTGCAGAATTCAGTGAATCATCGAATCTTTGAACGCACCTTGCGCCCCTTG<br>GTATTCCGAGGGGCACGCCTGTTTGAGTGTCGTGAAATTCTCAACTCGATCTTTTTGTTT<br>ACAAAAAGGTTGGGCTTGGACTTGGAGGCTTGCCGGCTCTGCAAAGACTCGGCTCCTC<br>TTGAATGCATGAGTGGGAACCTTTGTAGGGTGACCTTTGGTGTGATAATTGTCTACGCC<br>GTTGGTCTGCCTTGCGAAATTATGGGTACCTGCTTCTAACCGTCTTTTCGGAGACAATTT<br>CTATGAAACTTGACCTCAAATCAGGCGGGACTACCCGCTGAACTTAAGCAT

Table S2: List of reference taxa and their corresponding ITS rDNA sequences used for molecular phylogeny assessment.

Species	GenBank accession number
D. fragilis strain DSM 105465	MK463979.1
D. longiuscula	MH085945.1
Hericium alpestre strain CBS 539.90	AY534580.1
D. leptodon	MN044064.1
Laxitexticum incrustatum	MK894038.1
Laxitexticum bicolor	AM269787.1
Hericium erinaceus	MZ342890.1
Hericium coralloides	MK643472.1
Hericium americanum	AY534581.1
D. rhizomorpha	MG020135.1

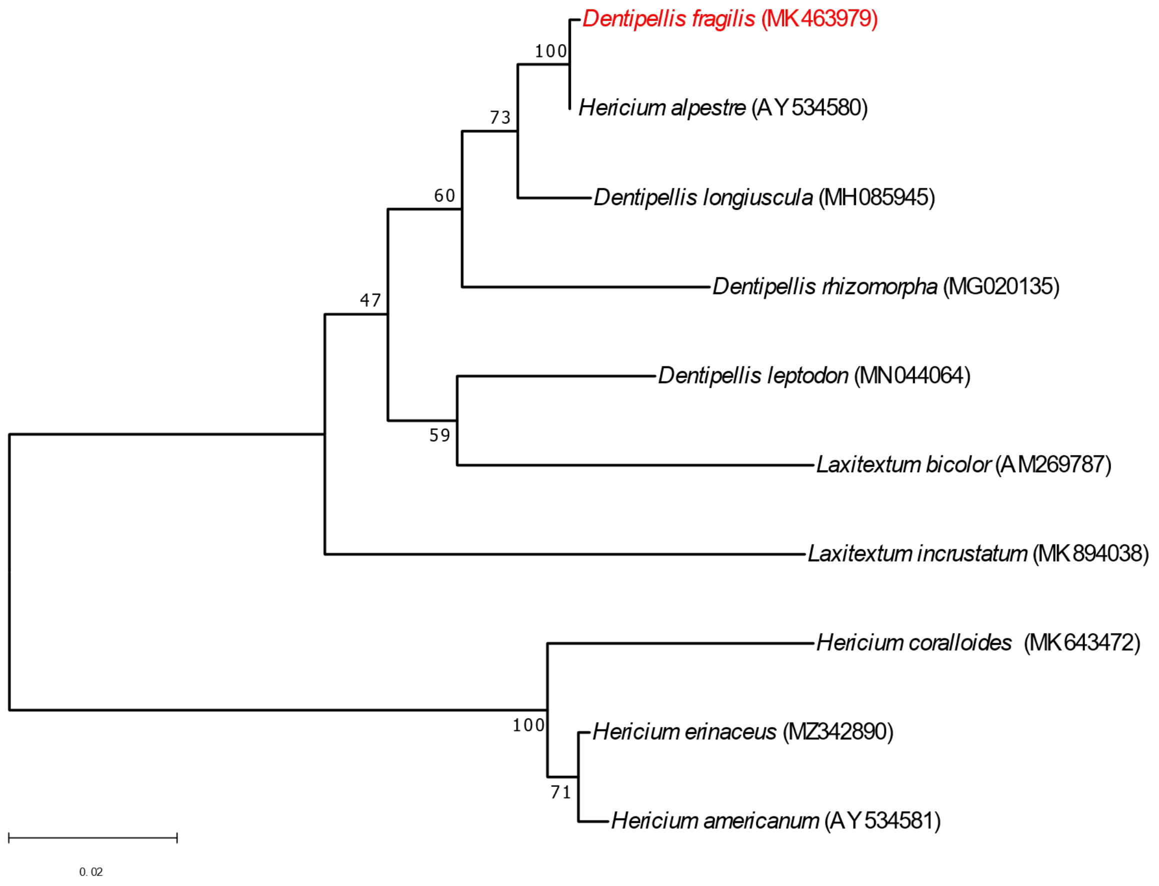

Figure S2: Phylogenetic tree, displaying the relationships of *D. fragilis* (highlighted in red) to selected species of the genera *Dentipellis*, *Hericium* and *Laxitextum* based on ITS ribosomal DNA (rDNA) sequences. Sequence alignment of the ITS rDNA was performed with ClustalW [1] and the tree was constructed using the neighbor-joining method [2] with 1000 Bootstrap replicates [3]. Evolutionary distances were computed with the Tamura-Nei model [4] and the rate variation among sites gamma distributed (gamma parameter = 1.00). GenBank accession numbers are given in parentheses. The scale bar represents 0.02 change per position. Analyses were done in MEGA 11 [5] (version 11.0.11) using default settings.

(3) Second fermentation and isolation of compound 8

For the second fermentation batch, *D. fragilis* was cultivated essentially as described in the main text. Fourteen 1 L Erlenmeyer, each containing 400 mL media, were incubated at 23 °C and 140 rpm for 34 days. The mycelia and the supernatant were separated by filtration through gaze. The culture broth was extracted by adding 5% (m/v) of Amberlite XAD™ 16N adsorber resin and stirring the resulting suspension for 5 h, followed by extracting the adsorber resin twice by adding two equivalent of ethyl acetate and stirring for 1 h. The separated organic layers were combined, dried over sodium sulfate and the solvent was evaporated to furnish 1463 mg of the supernatant crude extract. The mycelium was extracted by adding an equal volume of acetone, stirring the resulting suspension for 10 min, followed by resting in an ultrasonic bath (40 °C) for 30 min. The further extraction was essentially performed as described in the main text to yield 193 mg of the mycelial crude extract.

The supernatant crude extract was pre-fractionated using a Reveleris X2 (W.R. Grace and Co., Columbia, MD, USA, cartridge and mobile phases as described in the main text) applying the following gradient: 0% B for 10 min, 0% to 50% B within 30 min, isocratic conditions at 50% B for 5 min, 50% to 100% B within 10 min, isocratic conditions at 100% B for 10 min. The fraction eluted at 22.6 min (143 mg) was further purified by preparative HPLC (PLC 2250, Gilson Inc., Middleton, WI, USA). The stationary phase was a Synergi™ Polar-RP (same features as described in the main text). Deionized H_2O + 0.1% FA (v/v) (solvent A) and ACN + 0.1% FA (v/v) (solvent B) were used as the eluents and the flow rate was set to 50 mL/min. The gradient was operated from isocratic conditions at 30% B for 5 min, from 30% to 100% B within 50 min, followed by holding at 100% B for 5 min. The fraction eluted at 37.5 min (2.10 mg) contained compounds **7** and **8**. The mycelial extract was initially pre-fractionated by preparative column chromatography on SiO_2 (column size 200 x 18 mm) by eluting subsequently with DCM:acetone (v/v) 10:0, 9:1, 5:5 and 0:10 as well as with MeOH (200 mL solvent for each elution step). The fraction eluted with DCM:acetone 9:1 (v/v) was further purified by preparative HPLC (PLC 2250, column, solvents and flow rate as described before) applying the following gradient: isocratic conditions at 45% B for 3 min, from 45% to 90 % B within 45 min, from 90% to 100% B within 1 min, holding at 100% B for 5 min. The fraction eluted at 26.5 min (3.50 mg) also contained compounds **7** and **8**. Thus, the fractions obtained from the supernatant and the mycelial extract were combined and purified by preparative HPLC (pump: K-1800, Knauer, Berlin, Germany; mixing chamber: 54384, Knauer; detector: SPD-6A V, Shimadzu Europa GmbH, Duisburg, Germany; Injector: 7125, Rheodyne LLC, Rohnert Park, CA, USA; flatbed recorder: BD12E, van Renesse Supplies B. V., Monster, Netherlands). The stationary phase was a Nucleodur® 100–10 SiOH (250x21 mm) (Macherey-Nagel GmbH & Co. KG, Düren, Germany). Solvent A consisted of MTBE:*n*-heptane [2:8 (v/v)] and solvent B of MTBE:MeOH [9:1 (v/v)]. Initially, the flow rate was set to 10 mL/min and isocratic

conditions at 10% B were applied for 6 min. Subsequently, the flow rate was increased to 20 mL/min and the following gradient was applied: isocratic conditions at 10% B for 18 min, from 10% to 30% B within 5 min, isocratic conditions at 30% B for 15 min, from 30% to 50% B within 5 min, isocratic conditions at 50% B for 4 min, holding at 100% B for 10 min. Compound **8** was collected at a retention time of 35.0 min (0.31 mg) along with compound **7** eluting at 31.0 min (1.35 mg).

(4) HPLC-DAD chromatograms of crude extracts

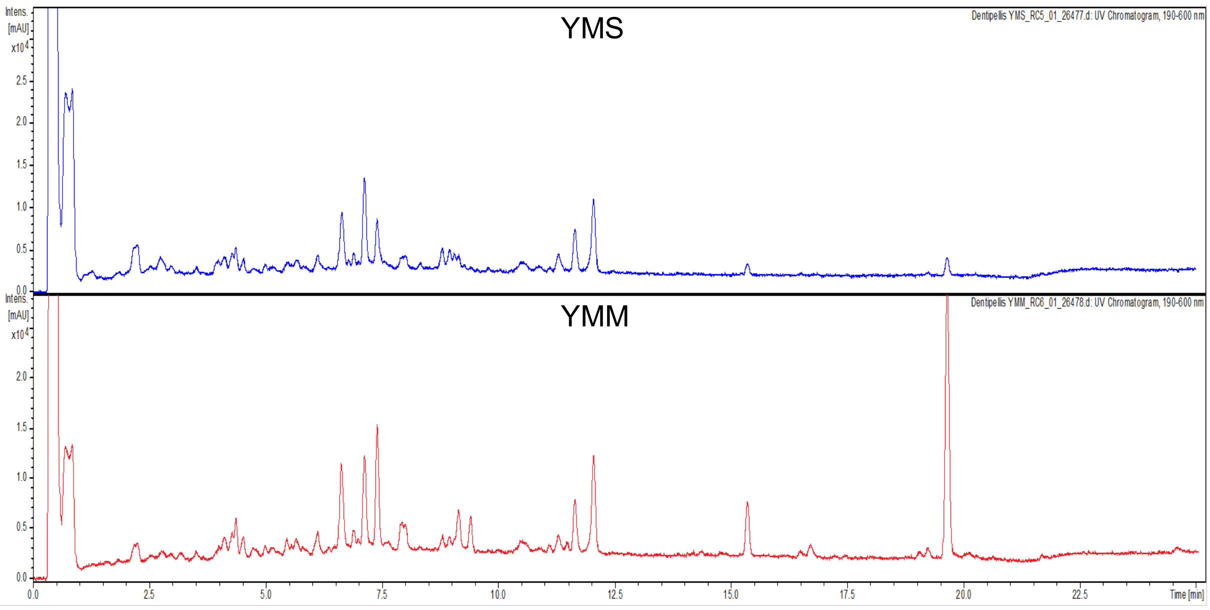

Figure S3: HPLC-DAD chromatograms (detection at 190–600 nm) of the supernatant (YMS) and mycelial (YMM) ethyl acetate crude extracts of *D. fragilis* DSM 105465, cultured in YMG liquid media.

(5) Analytical data of compound 1

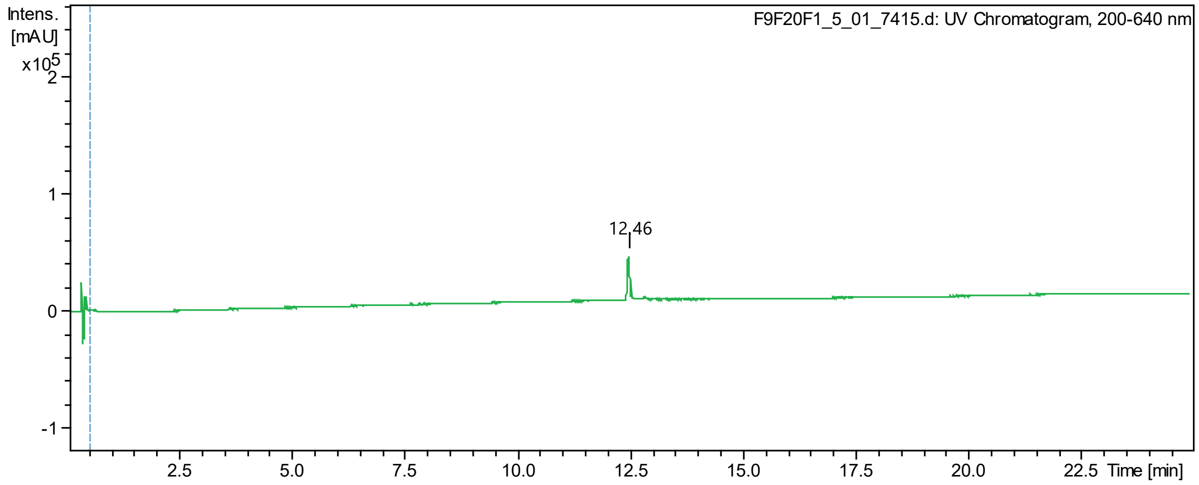

Figure S4: HPLC-DAD chromatogram of compound **1**.

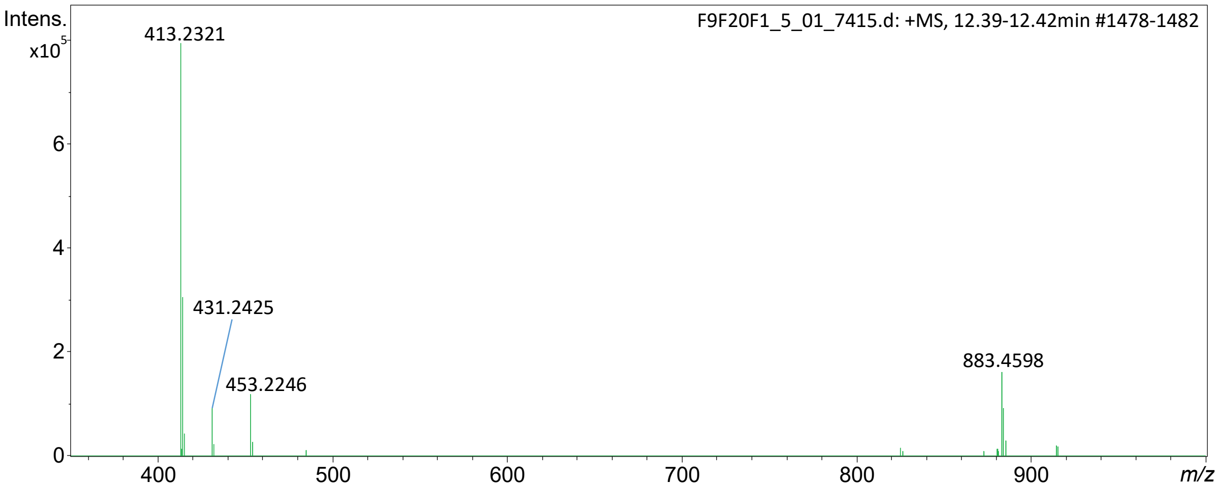

Figure S5: HR-(+)ESIMS spectrum of compound **1**.

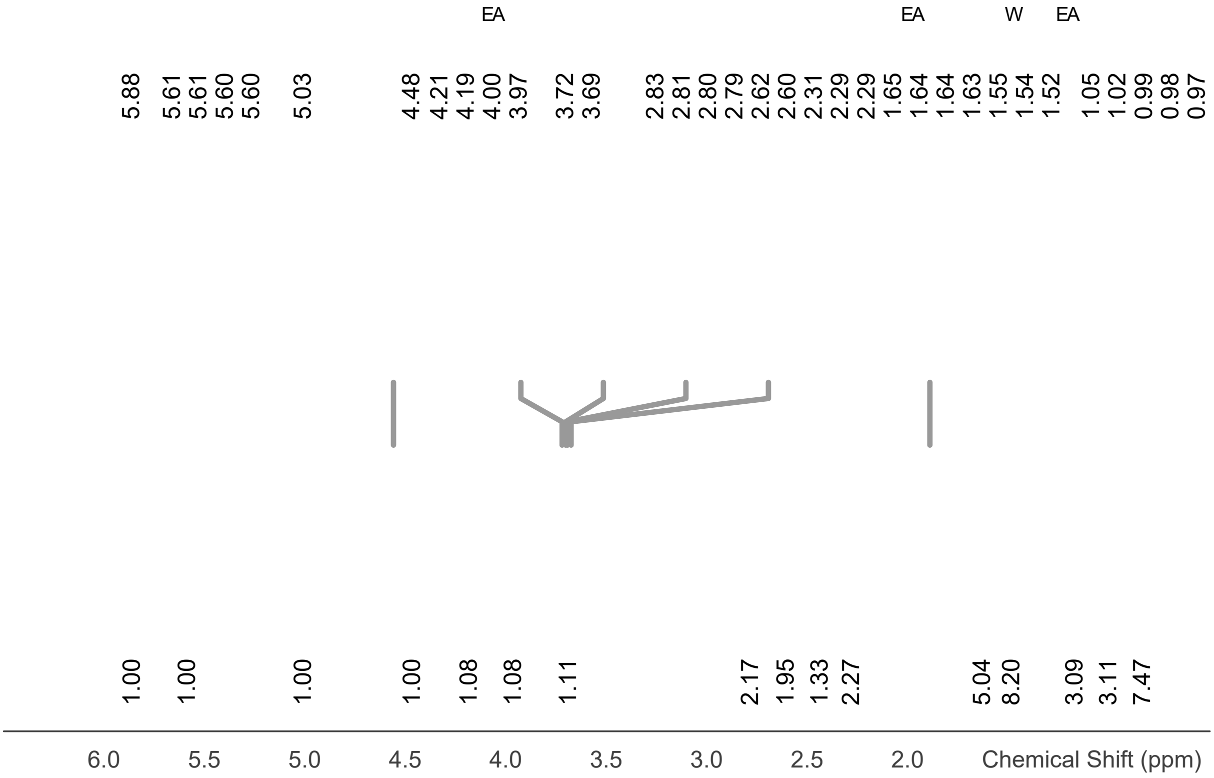

Figure S6: ^{1}H NMR spectrum (500 MHz, $CDCl_3$, 298 K) of the purified compound **1**. W: H_2O, EA: ethyl acetate.

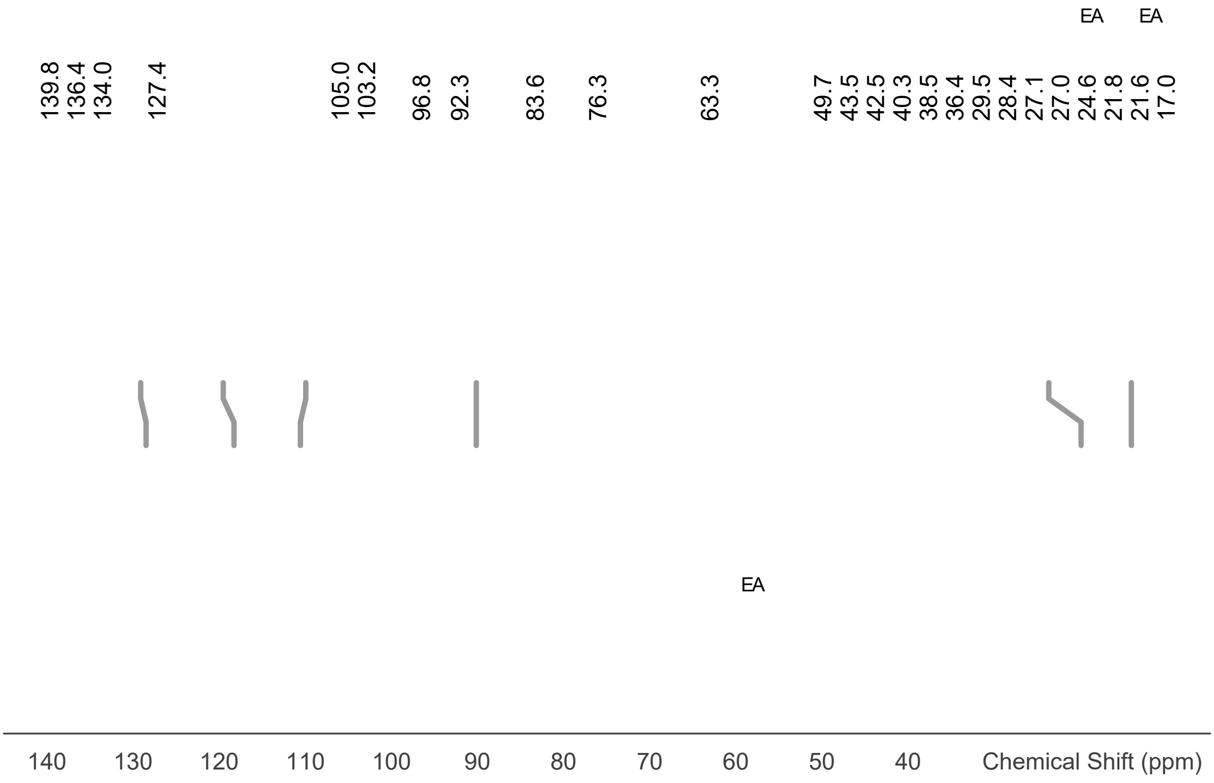

Figure S7: ^{13}C NMR spectrum (125 MHz, $CDCl_3$, 298 K) of the purified compound **1**. EA: ethyl acetate.

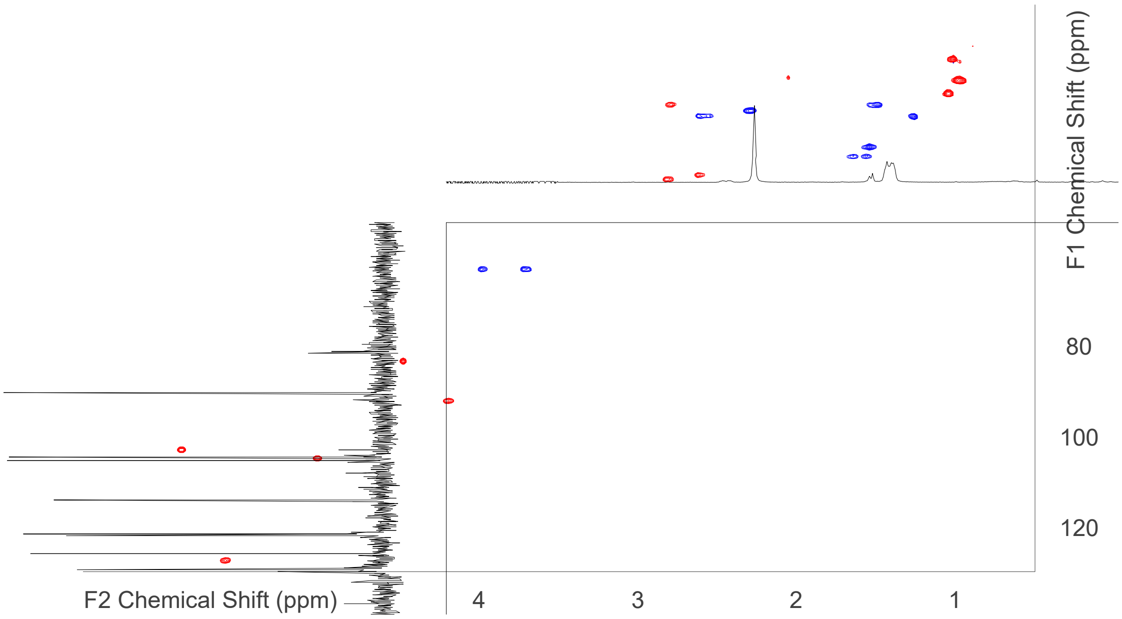

Figure S8: ^{1}H,^{13}C HSQC-DEPT spectrum (500 MHz, $CDCl_3$, 298 K) of the purified compound **1**.

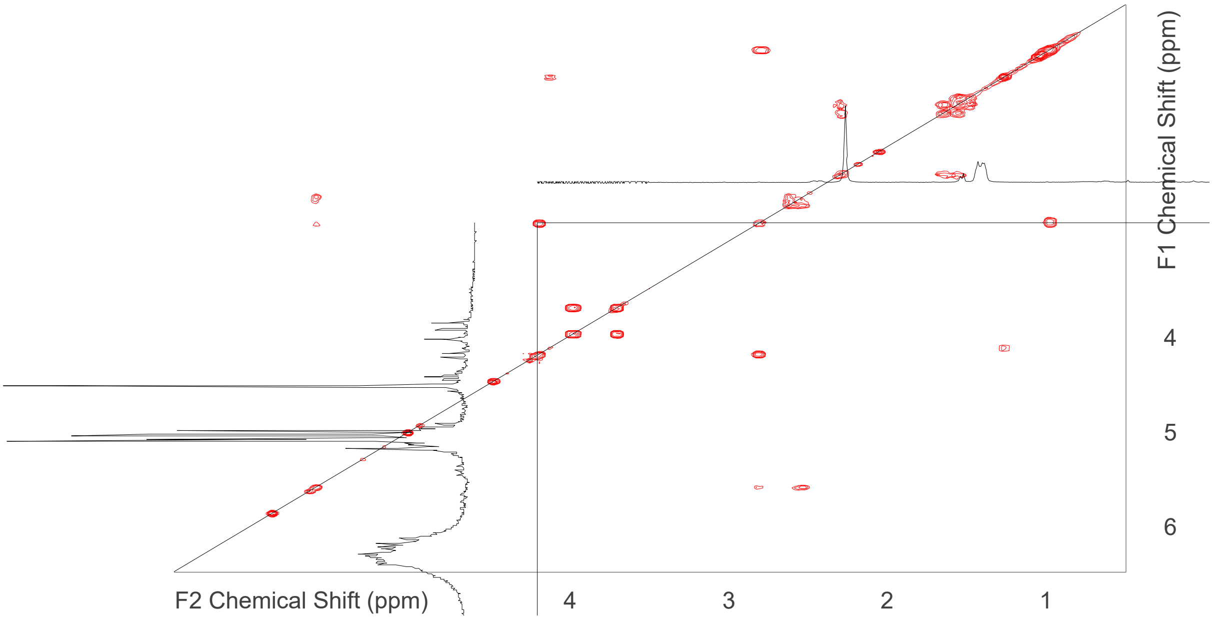

Figure S9: ^{1}H,^{1}H COSY spectrum (500 MHz, $CDCl_3$, 298 K) of the purified compound **1**.

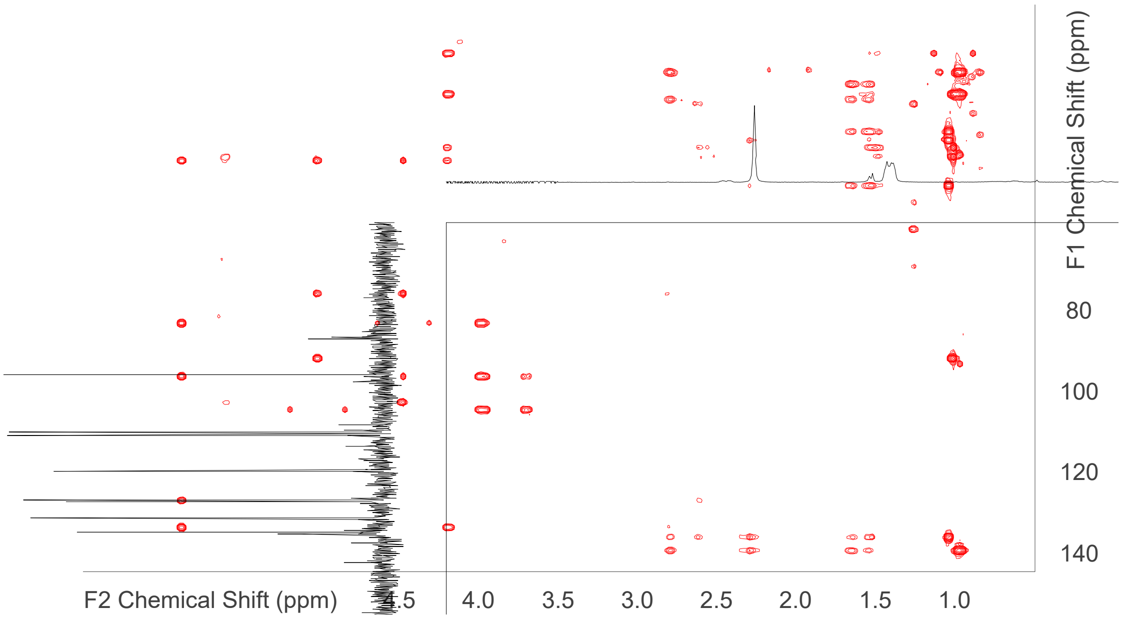

Figure S10: $^{1}H,^{13}C$ HMBC spectrum (500 MHz, $CDCl_3$, 298 K) of the purified compound **1**.

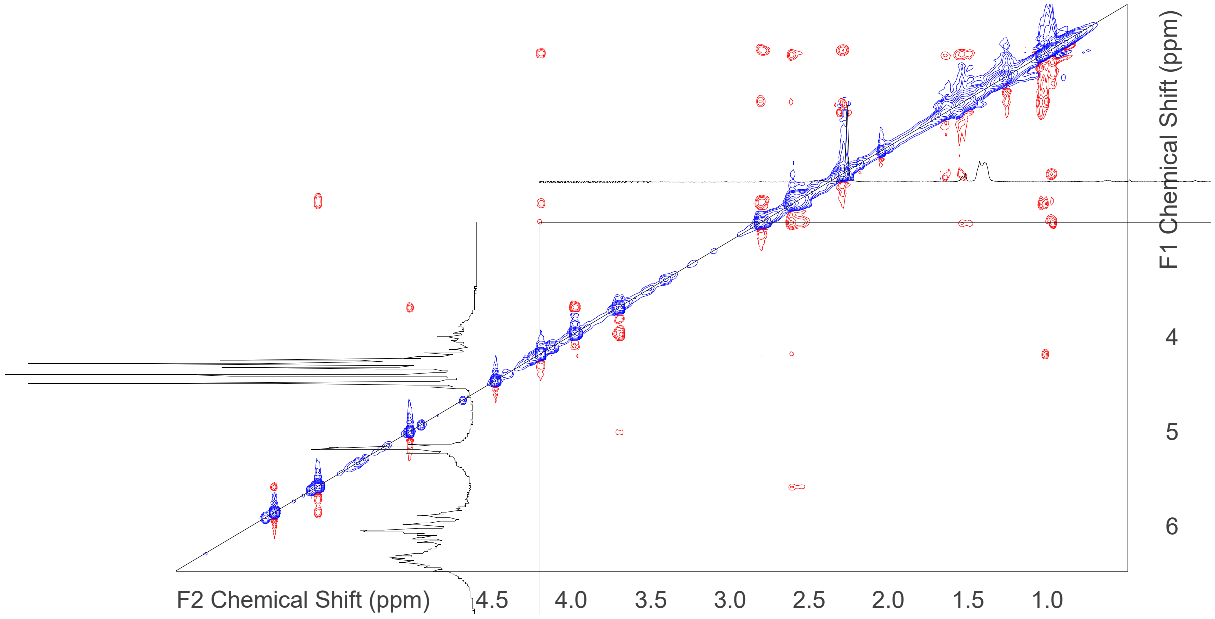

Figure S11: $^{1}H,^{1}H$ ROESY spectrum (500 MHz, $CDCl_3$, 298 K) of the purified compound **1**.

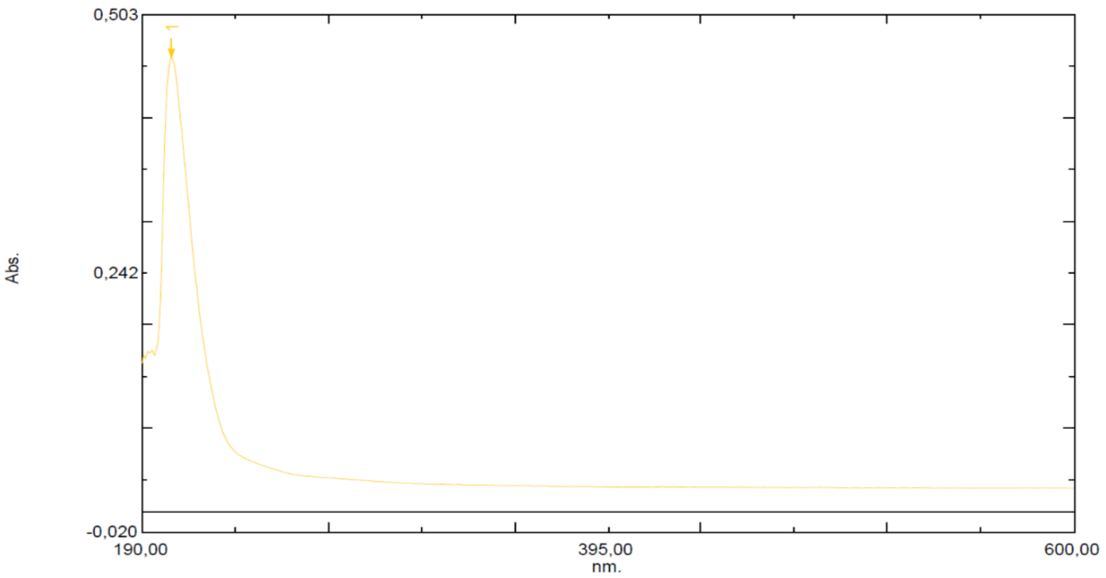

Figure S12: UV/VIS spectrum of compound **1** in MeOH.

(6) Analytical data of compound 2

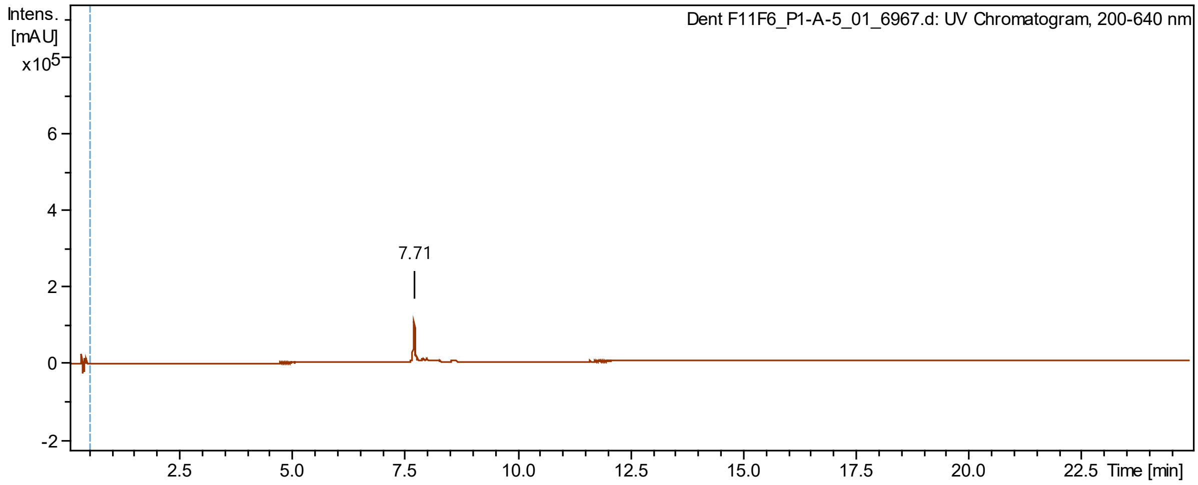

Figure S13: HPLC-DAD chromatogram of compound **2**.

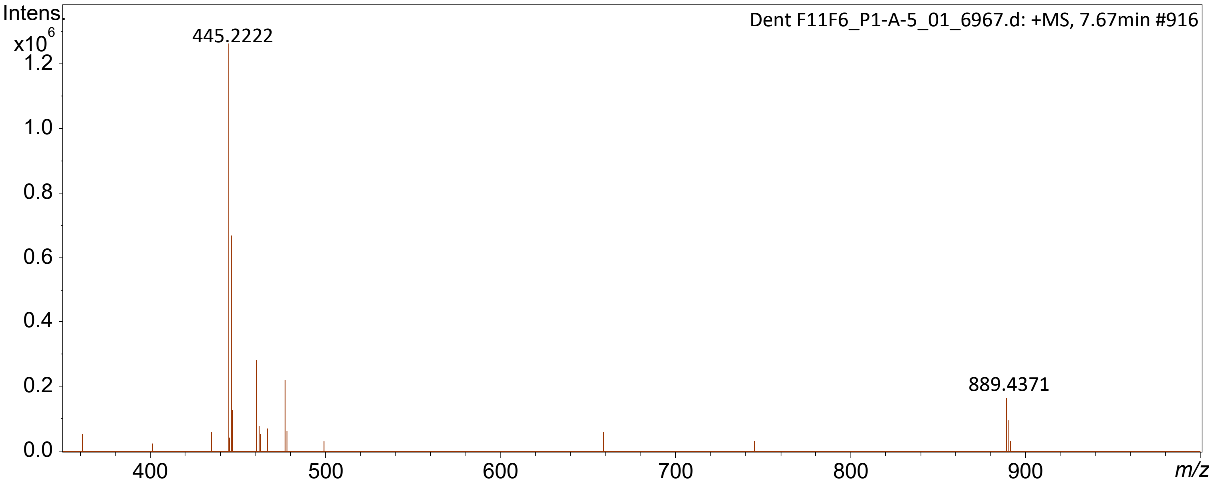

Figure S14: HR-(+)ESIMS spectrum of compound **2**.

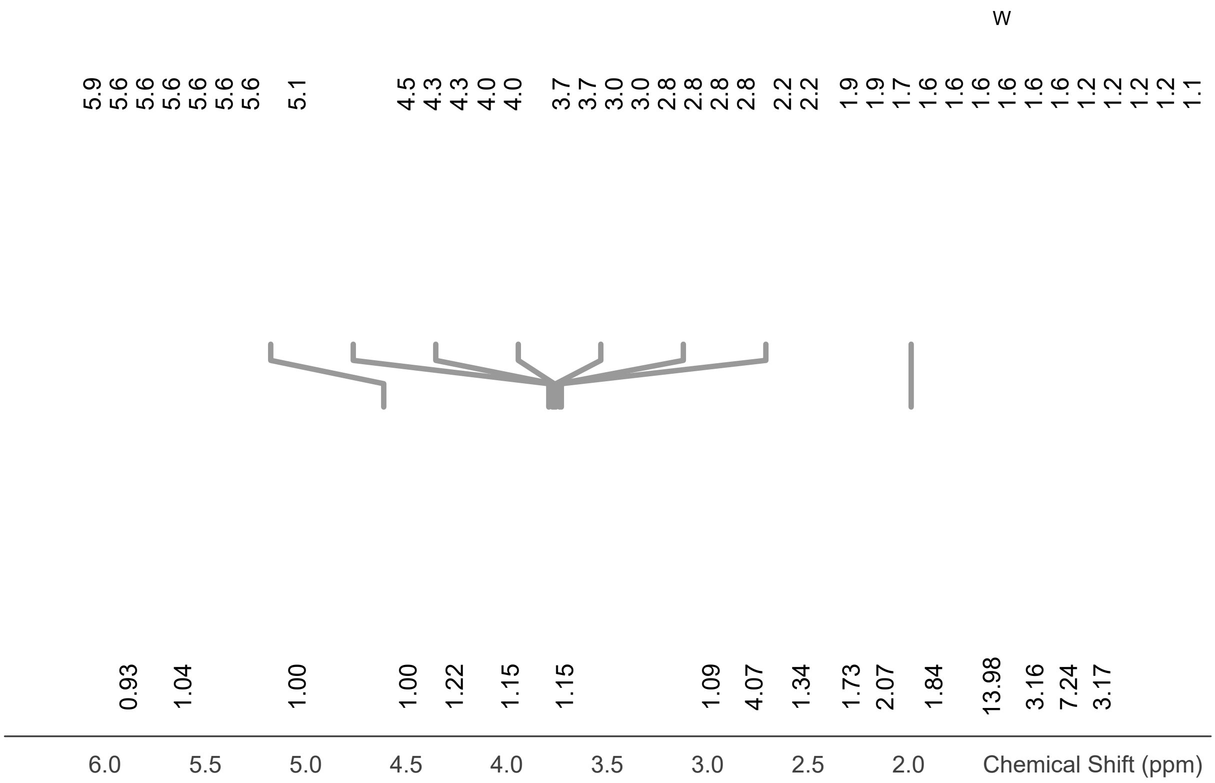

Figure S15: ^{1}H NMR spectrum (500 MHz, $CDCl_3$, 298 K) of the purified compound **2**. W: H_2O.

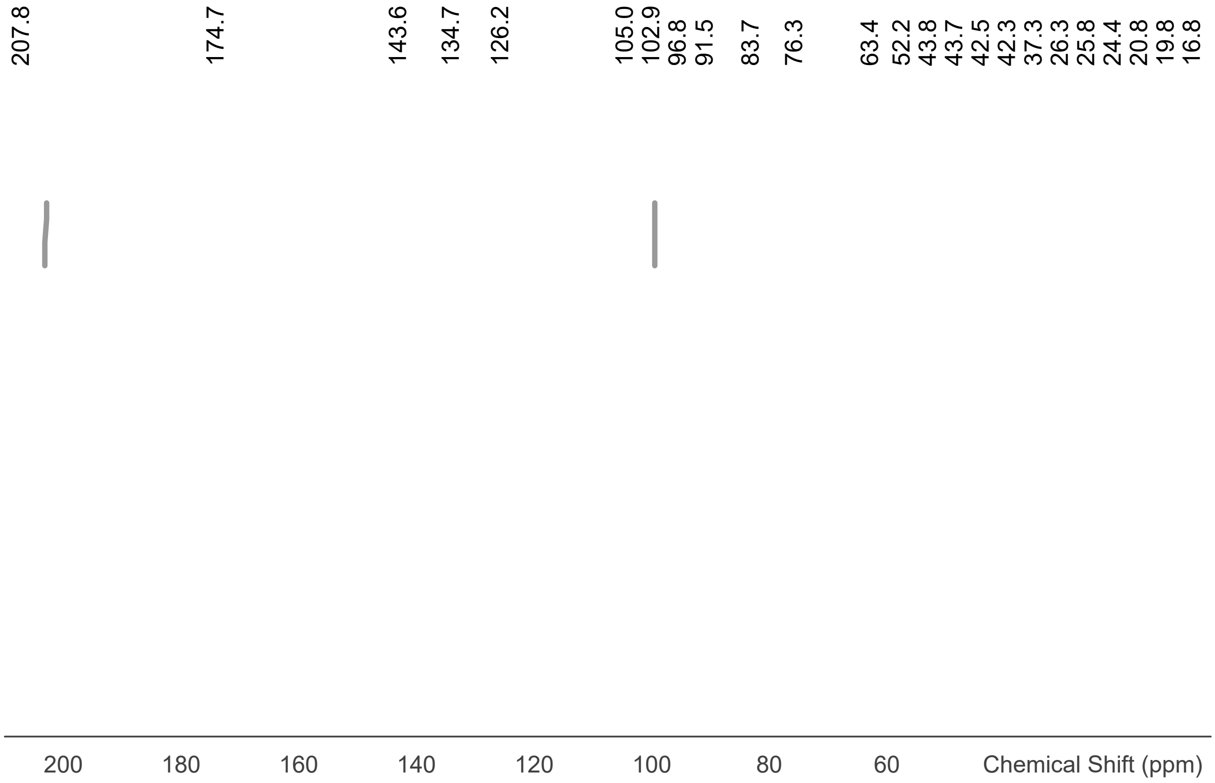

Figure S16: ^{13}C NMR spectrum (125 MHz, $CDCl_3$, 298 K) of the purified compound **2**.

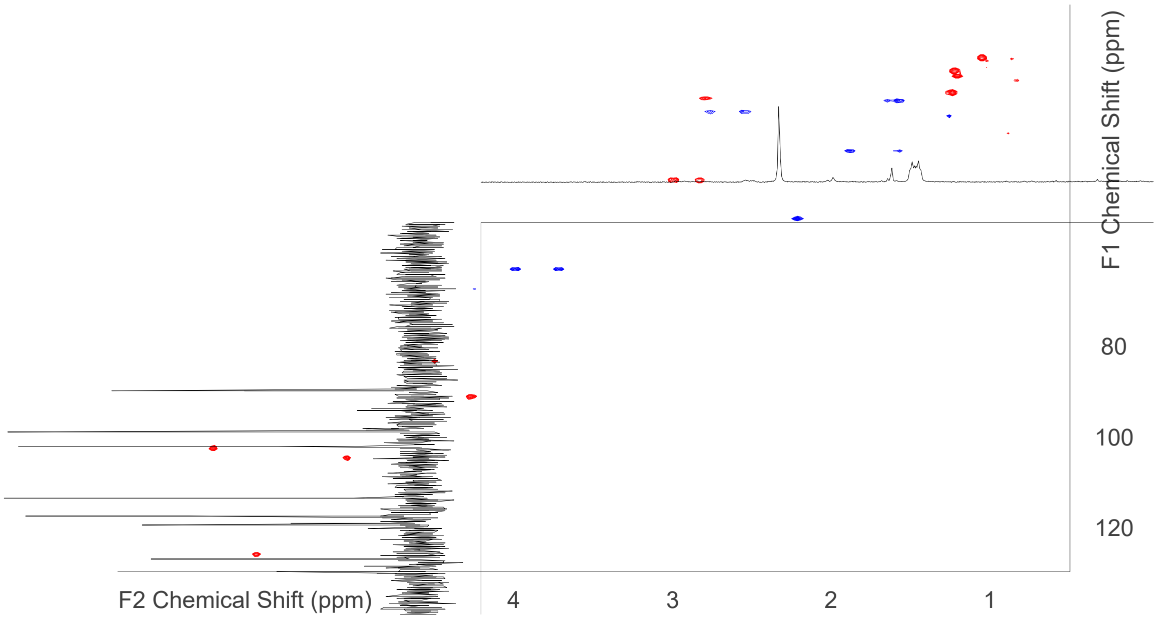

Figure S17: $^{1}H,^{13}C$ HSQC-DEPT spectrum (500 MHz, $CDCl_3$, 298 K) of the purified compound **2**.

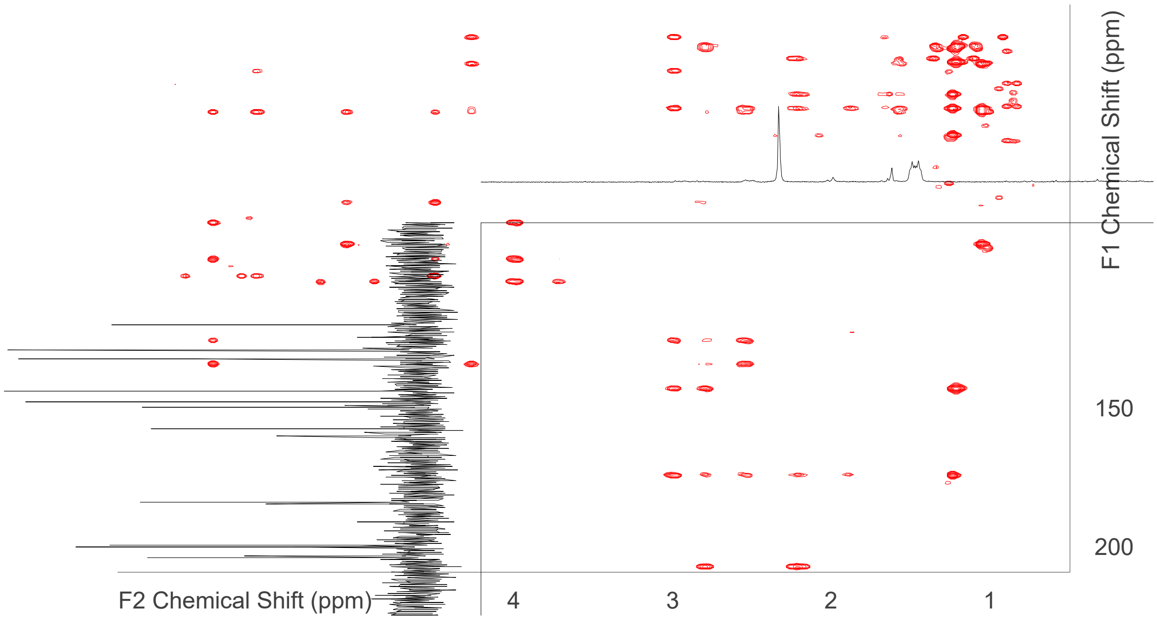

Figure S18: $^{1}H,^{13}C$ HMBC spectrum (500 MHz, $CDCl_3$, 298 K) of the purified compound **2**.

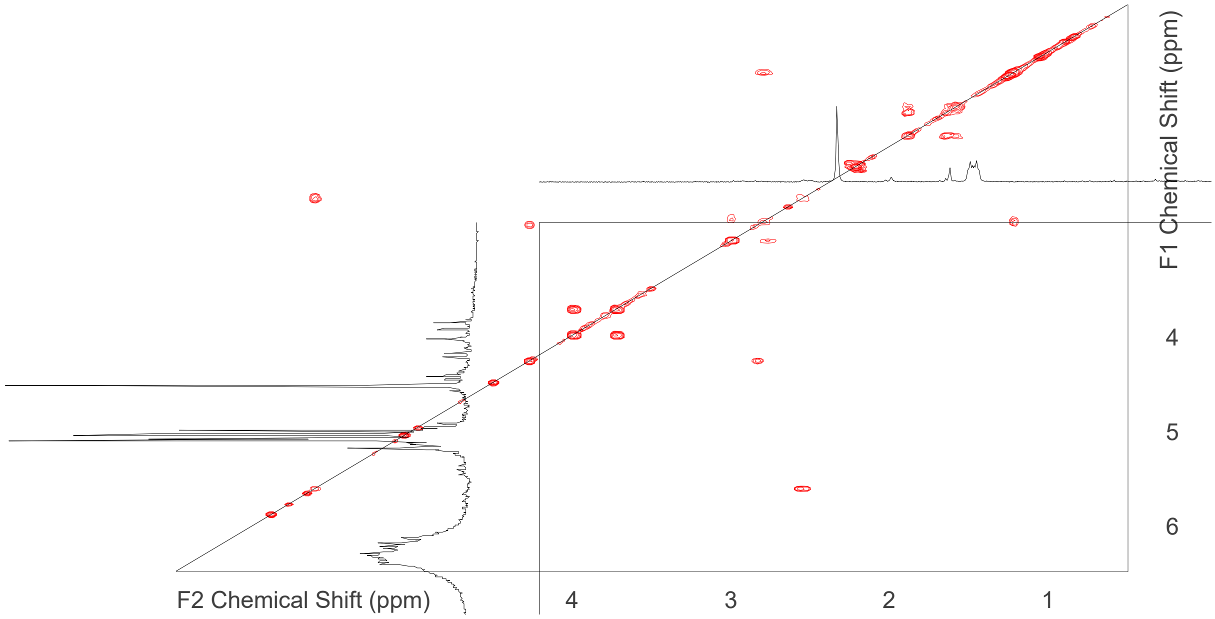

Figure S19: ^{1}H,^{1}H COSY spectrum (500 MHz, $CDCl_3$, 298 K) of the purified compound **2**.

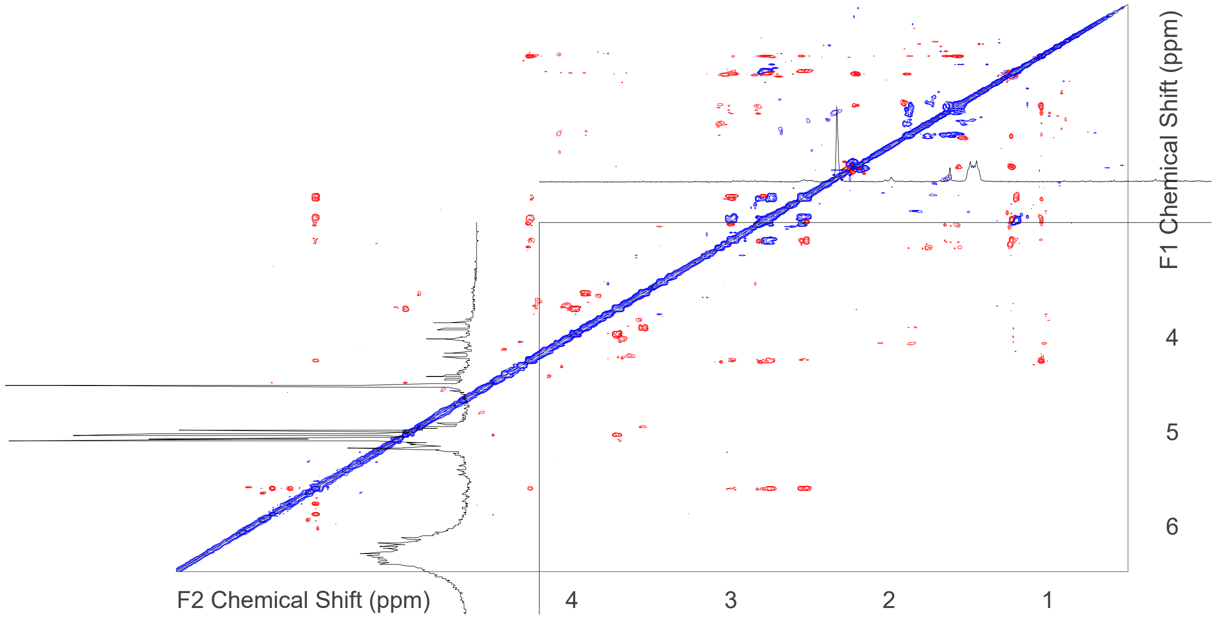

Figure S20: ^{1}H,^{1}H ROESY spectrum (500 MHz, $CDCl_3$, 298 K) of the purified compound **2**.

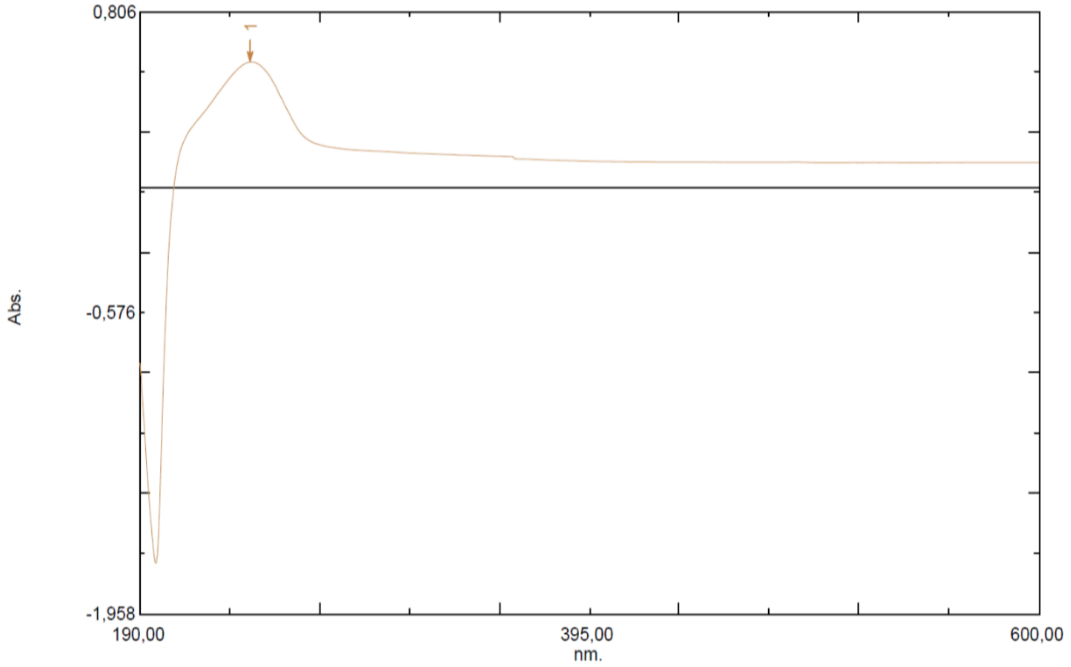

Figure S21: UV/VIS spectrum of compound **2** in MeOH.

(7) Analytical data of compound 3

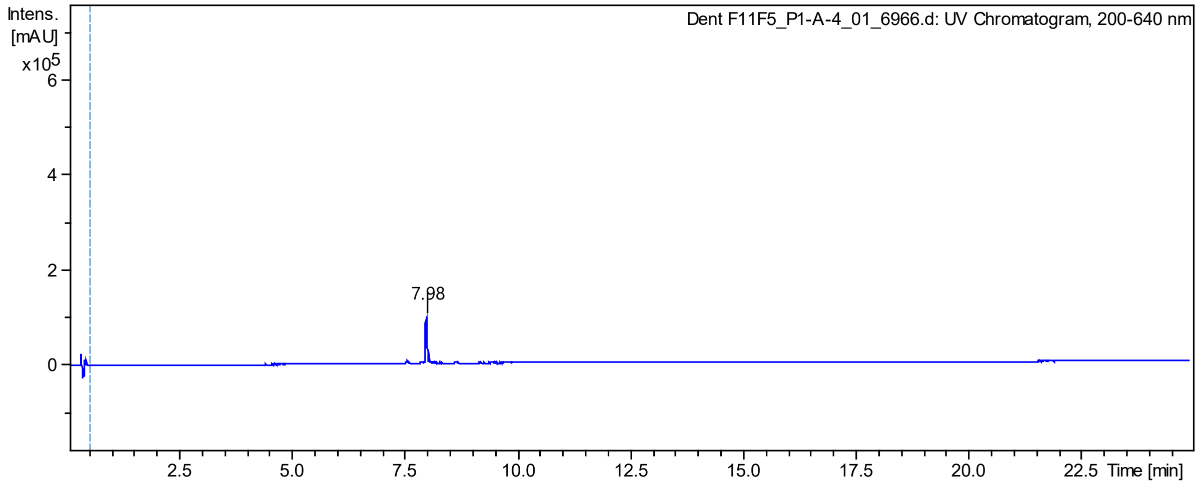

Figure S22: HPLC-DAD chromatogram of compound **3**.

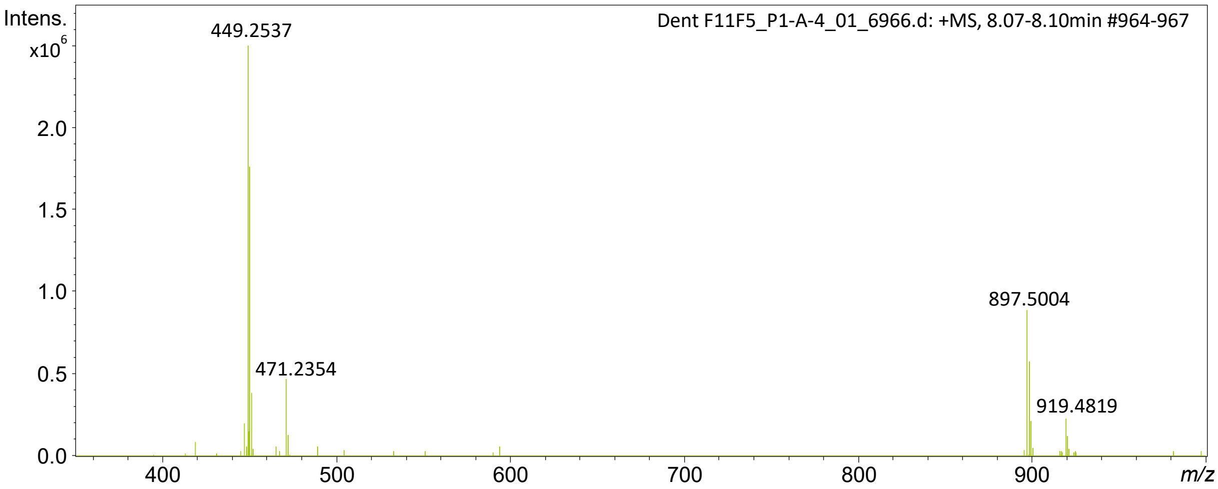

Figure S23: HR-(+)ESIMS spectrum of compound **3**.

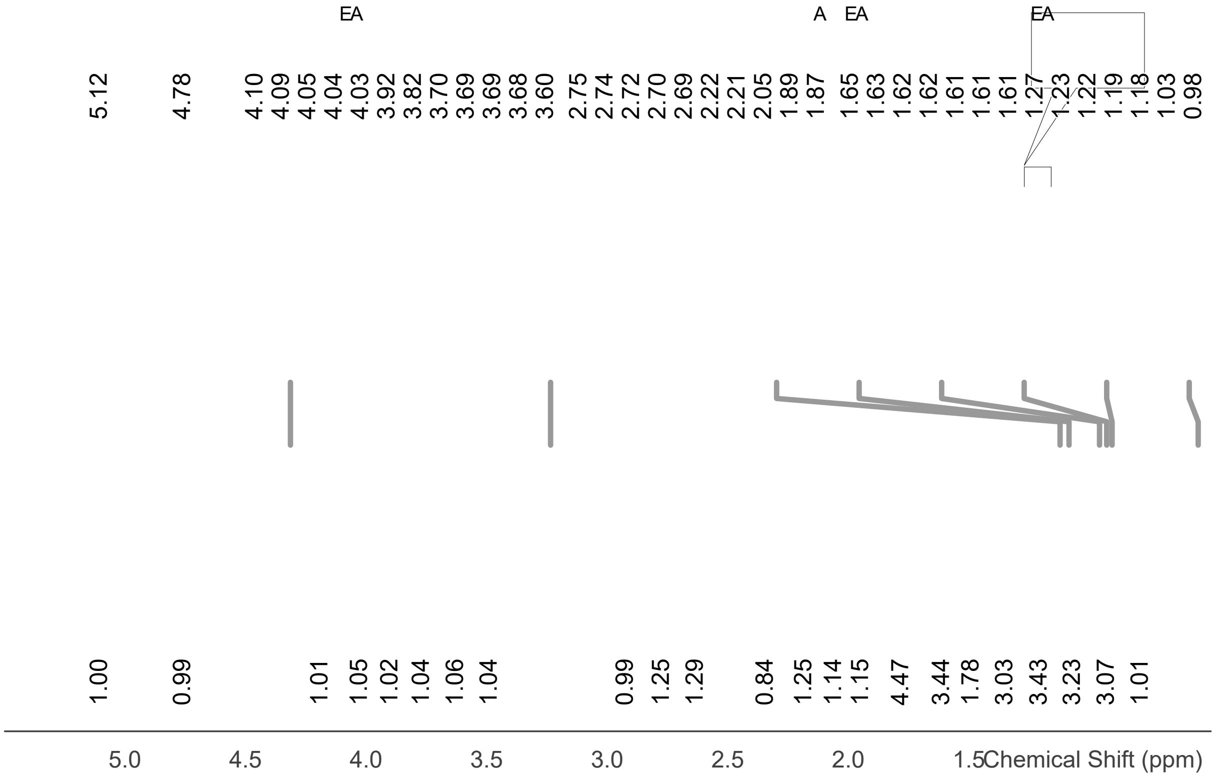

Figure S24: ^{1}H NMR spectrum (700 MHz, $CDCl_3$, 298 K) of the purified compound **3**. EA: ethyl acetate, A: acetone.

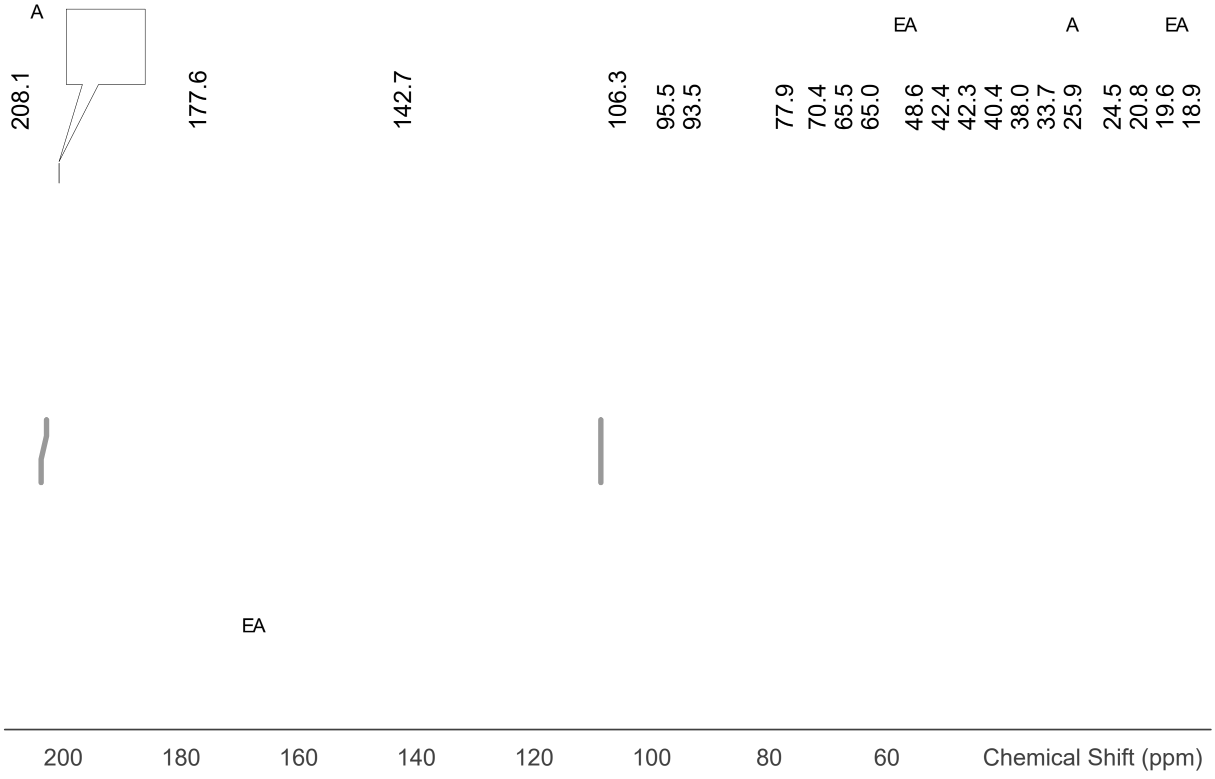

Figure S25: ^{13}C NMR spectrum (175 MHz, $CDCl_3$, 298 K) of the purified compound **3**. EA: ethyl acetate, A: acetone.

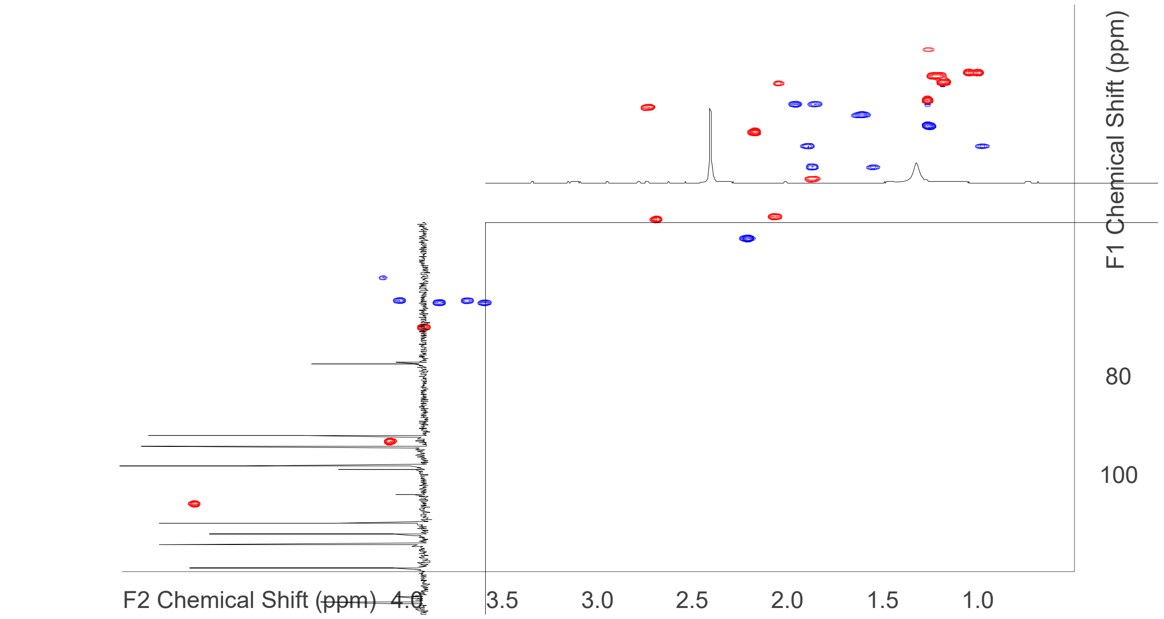

Figure S26: ^{1}H,^{13}C HSQC-DEPT spectrum (700 MHz, $CDCl_3$, 298 K) of the purified compound **3**.

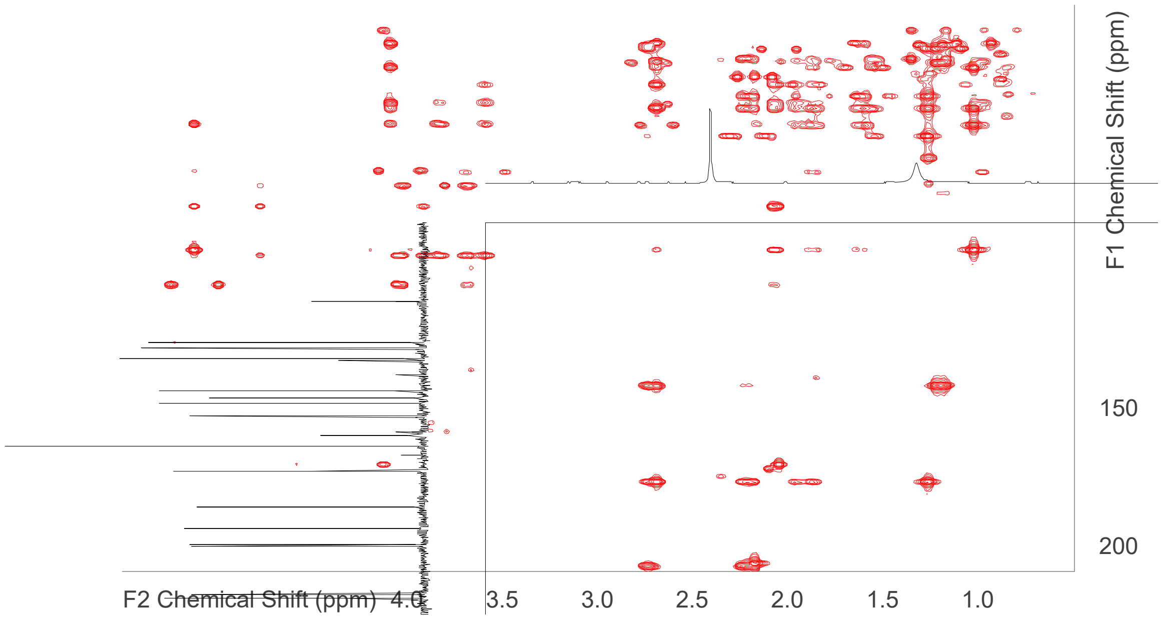

Figure S27: ^{1}H,^{13}C HMBC spectrum (700 MHz, $CDCl_3$, 298 K) of the purified compound **3**.

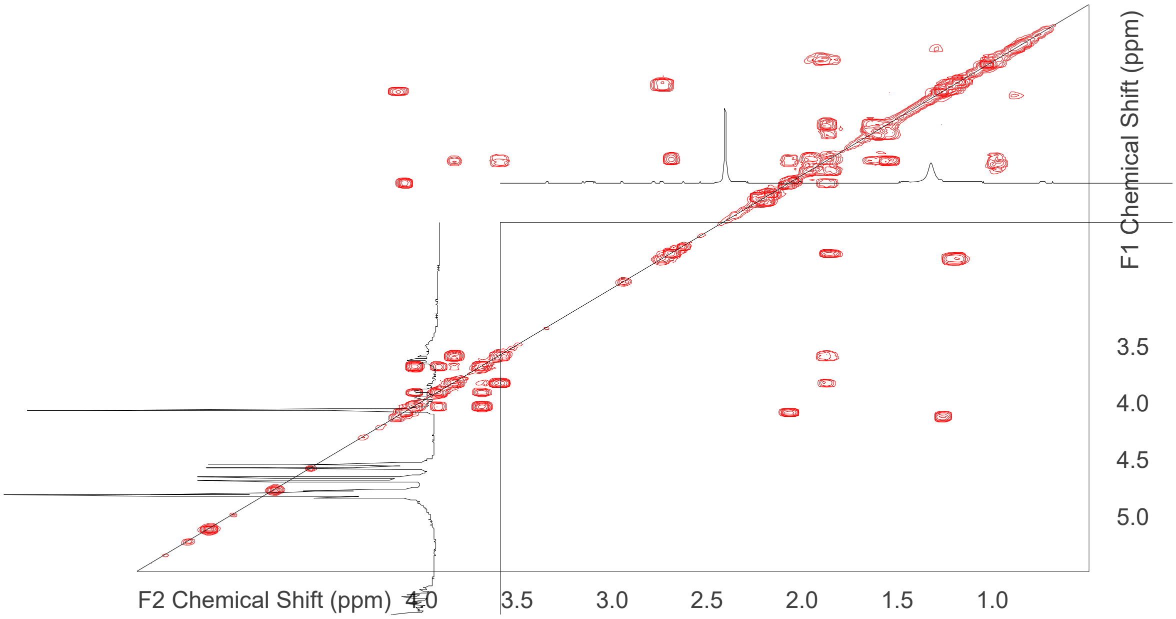

Figure S28: ^{1}H,^{1}H COSY spectrum (700 MHz, $CDCl_3$, 298 K) of the purified compound **3**.

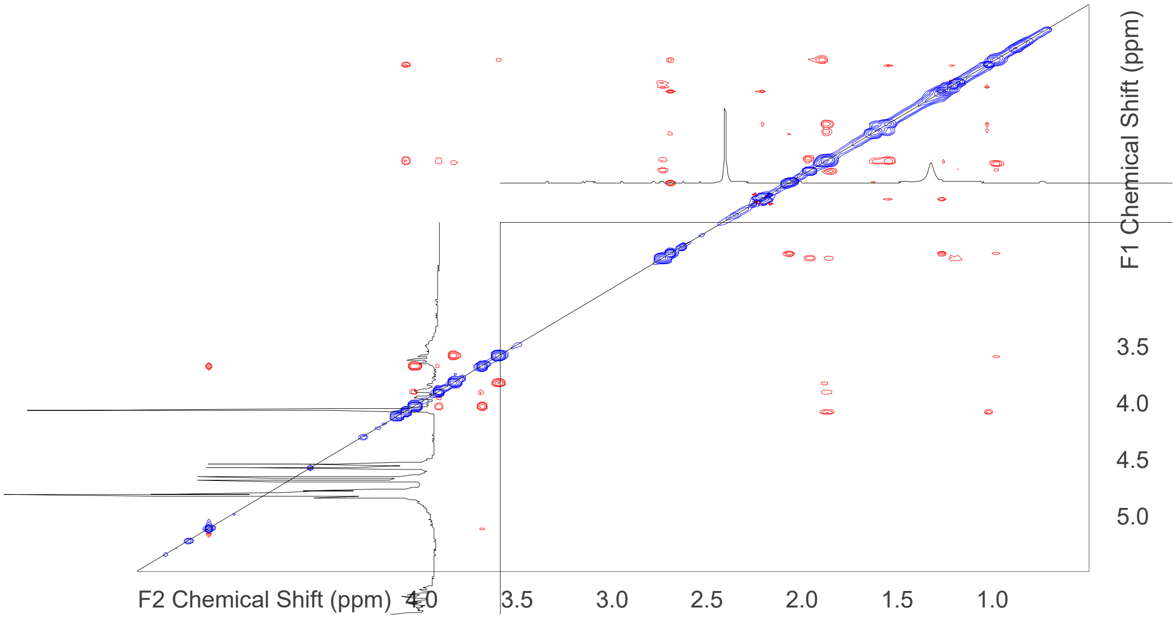

Figure S29: ^{1}H,^{1}H ROESY spectrum (700 MHz, $CDCl_3$, 298 K) of the purified compound **3**.

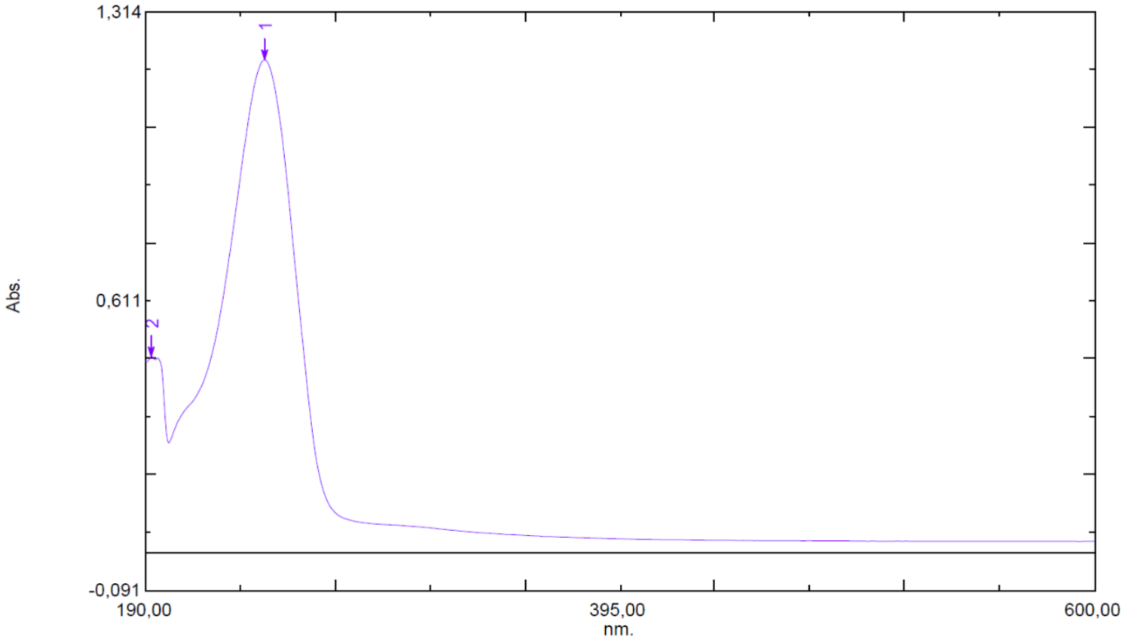

Figure S30: UV/VIS spectrum of compound **3** in MeOH.

(8) Analytical data of compound 4

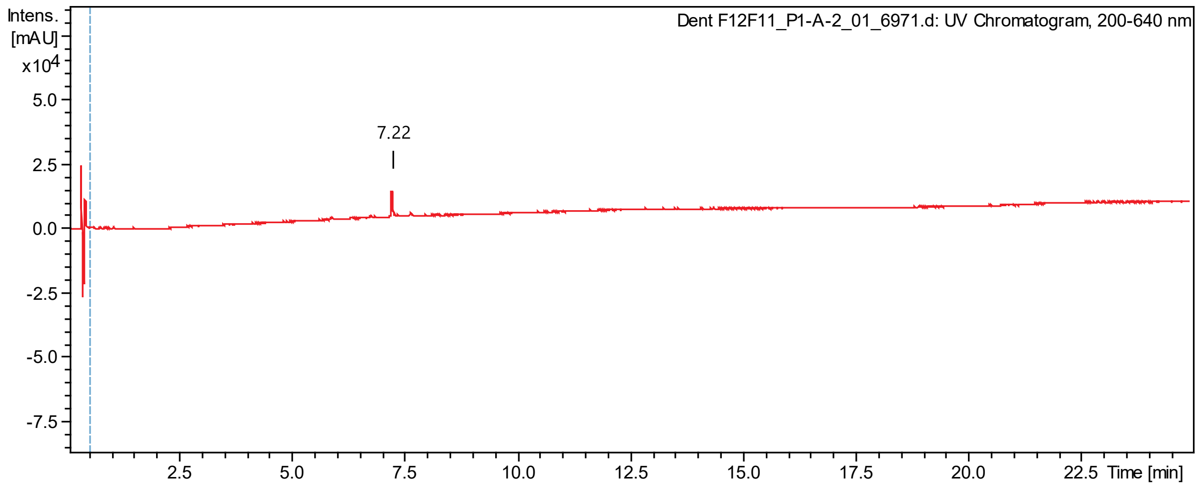

Figure S31: HPLC-DAD chromatogram of compound **4**.

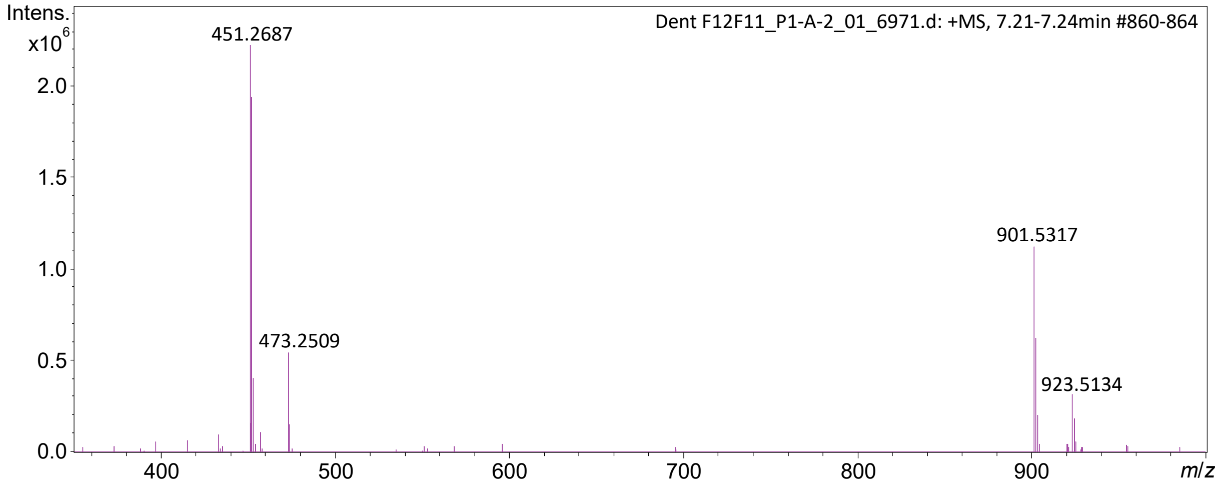

Figure S32: HR-(+)ESIMS spectrum of compound **4**.

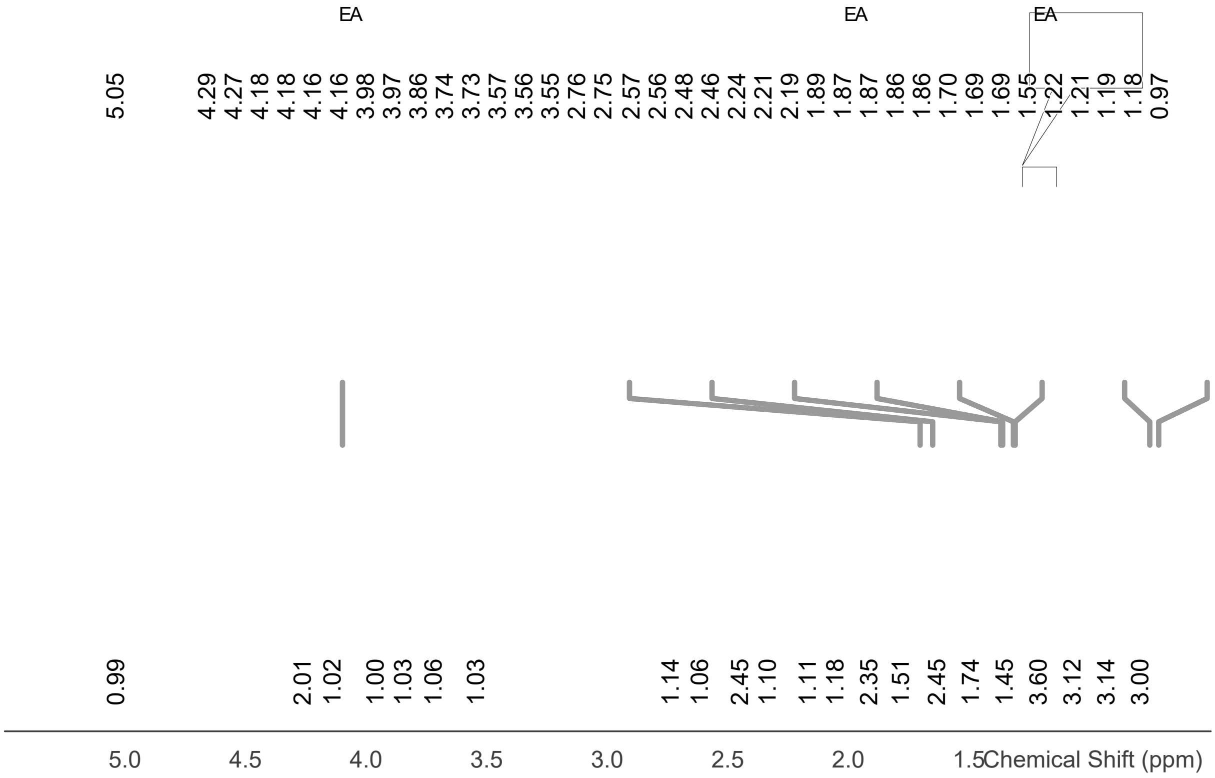

Figure S33: ^{1}H NMR spectrum (700 MHz, $CDCl_3$, 298 K) of the purified compound **4**. EA: ethyl acetate.

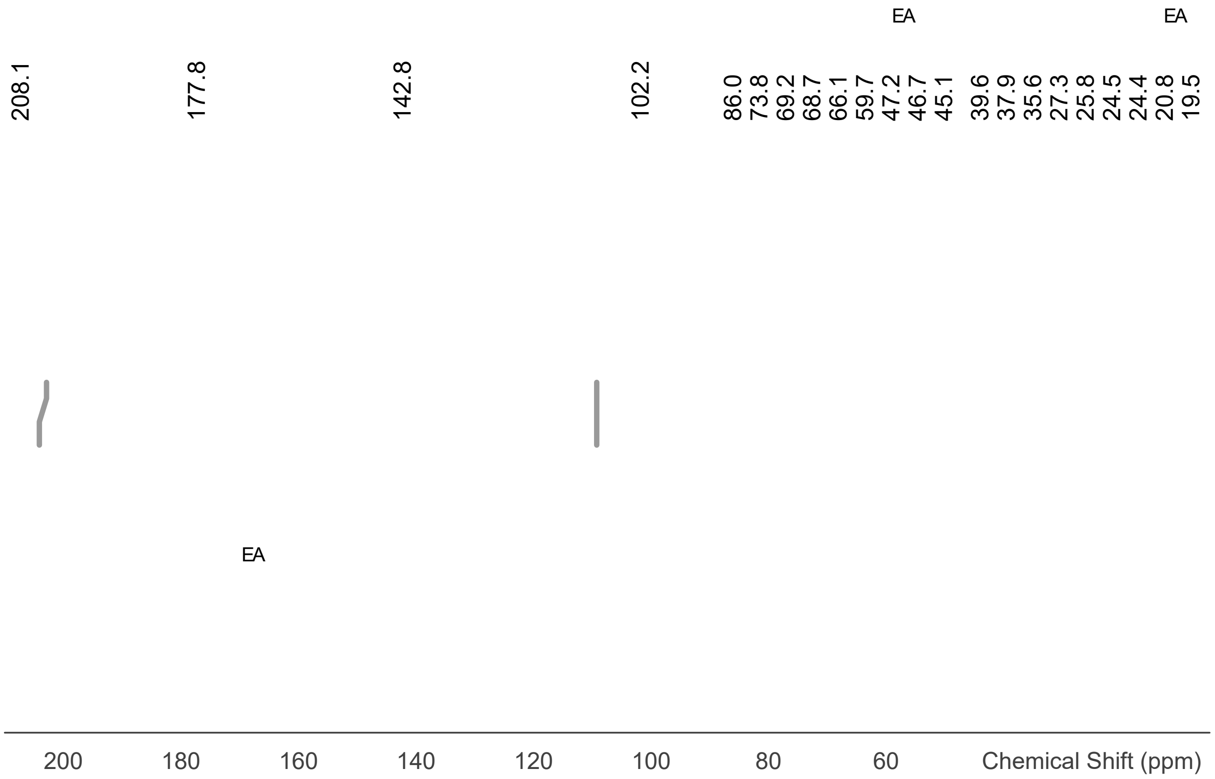

Figure S34: ^{13}C NMR spectrum (175 MHz, $CDCl_3$, 298 K) of the purified compound **4**. EA: ethyl acetate.

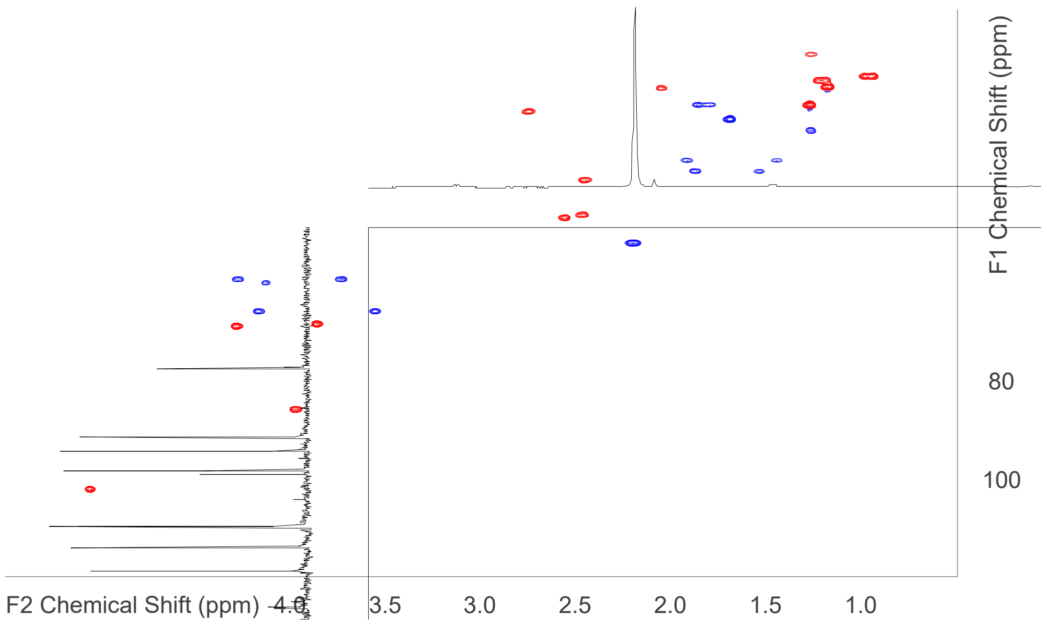

Figure S35: $^{1}H,^{13}C$ HSQC-DEPT spectrum (700 MHz, $CDCl_3$, 298 K) of the purified compound **4**.

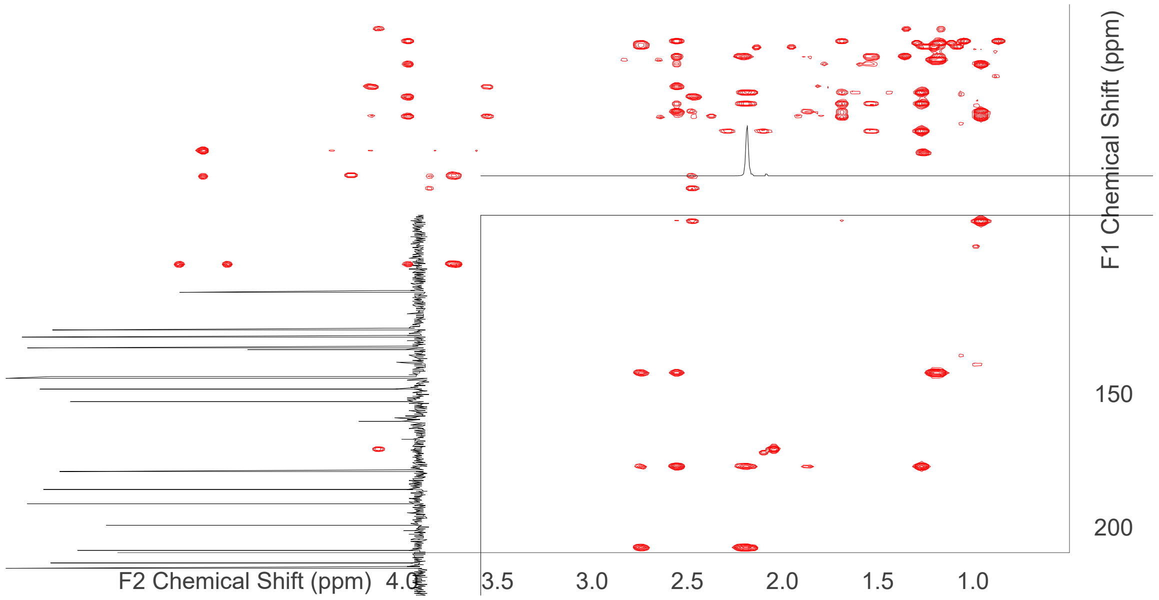

Figure S36: $^{1}H,^{13}C$ HMBC spectrum (700 MHz, $CDCl_3$, 298 K) of the purified compound **4**.

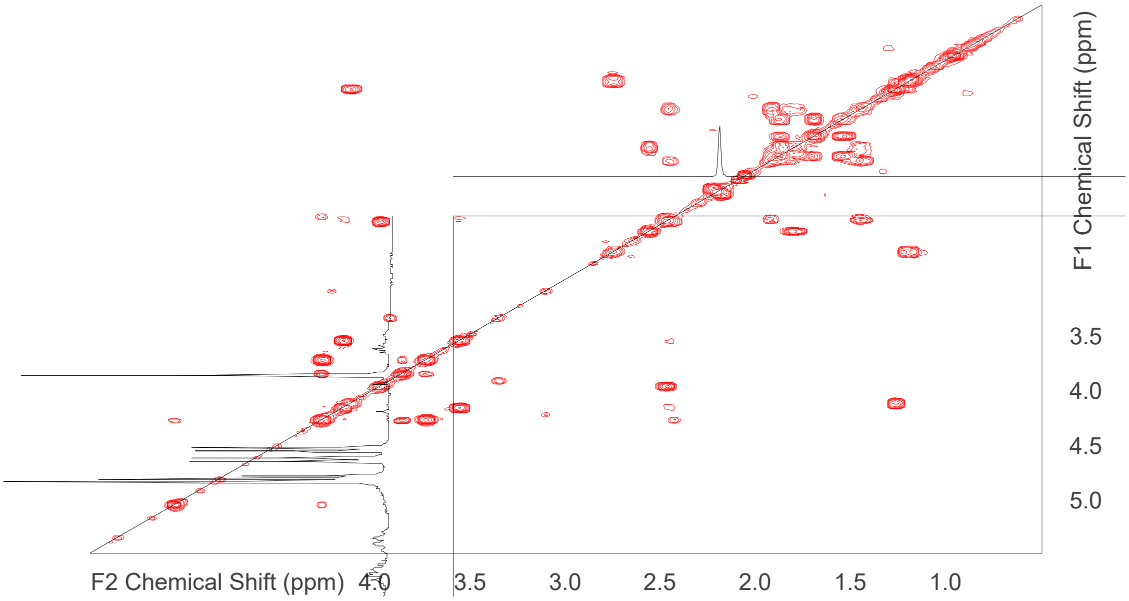

Figure S37: $^{1}H,^{1}H$ COSY spectrum (700 MHz, $CDCl_3$, 298 K) of the purified compound **4**.

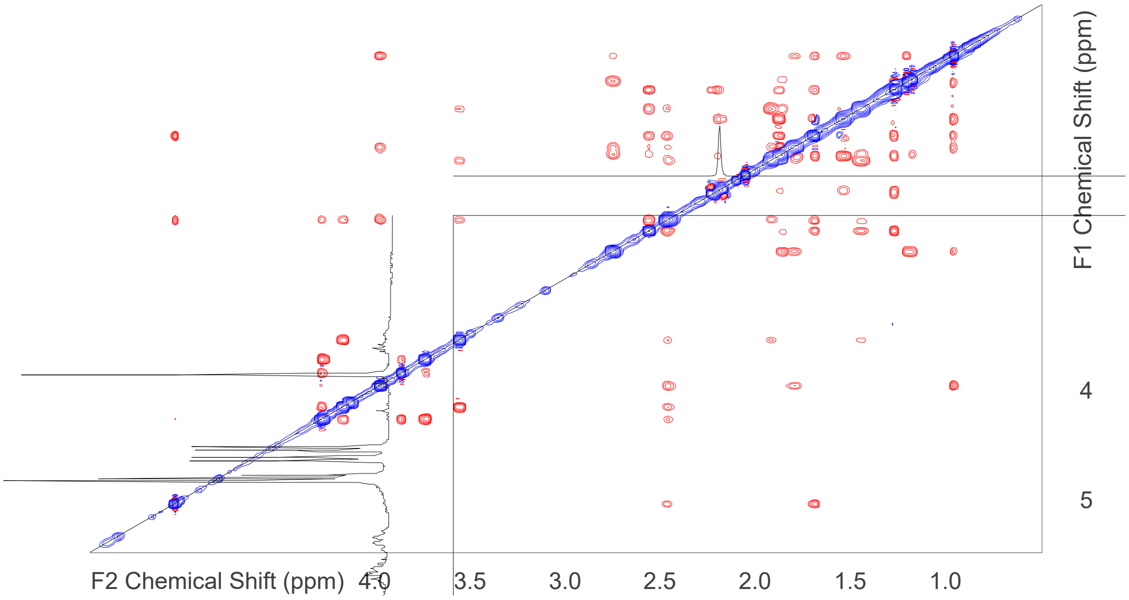

Figure S38: $^{1}H,^{1}H$ ROESY spectrum (700 MHz, $CDCl_3$, 298 K) of the purified compound **4**.

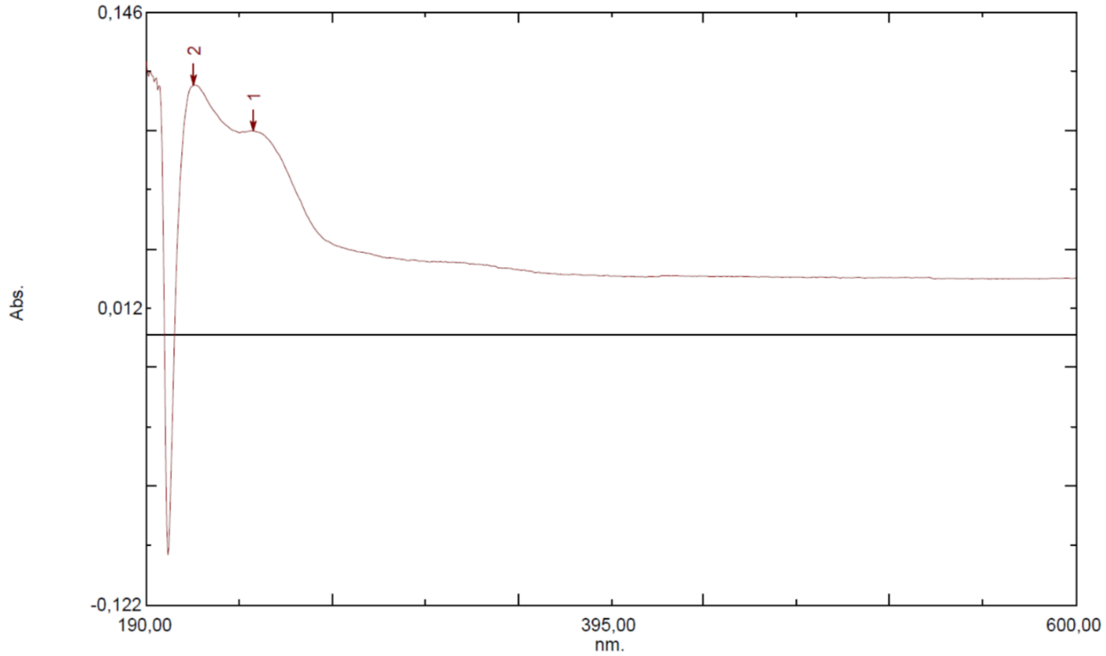

Figure S39: UV/VIS spectrum of compound **4** in MeOH.

(9) Analytical data of compound 5

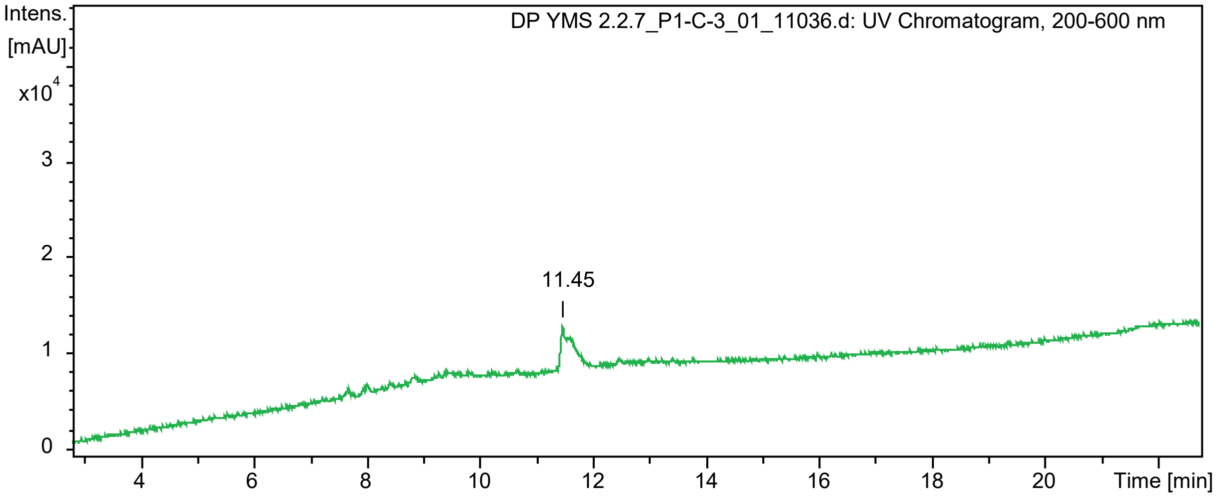

Figure S40: HPLC-DAD chromatogram of compound **5**.

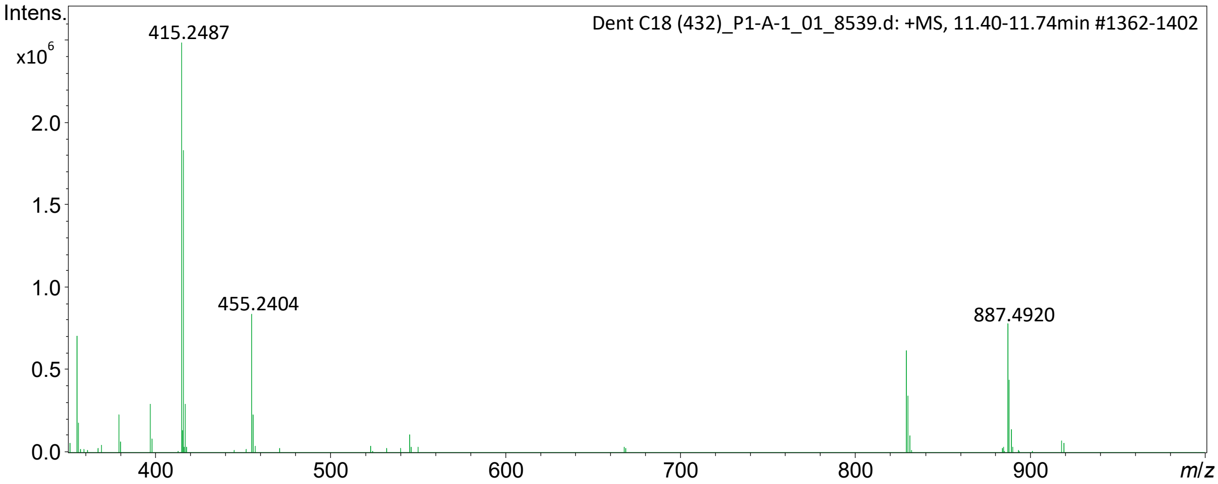

Figure S41: HR-(+)ESIMS spectrum of compound **5**.

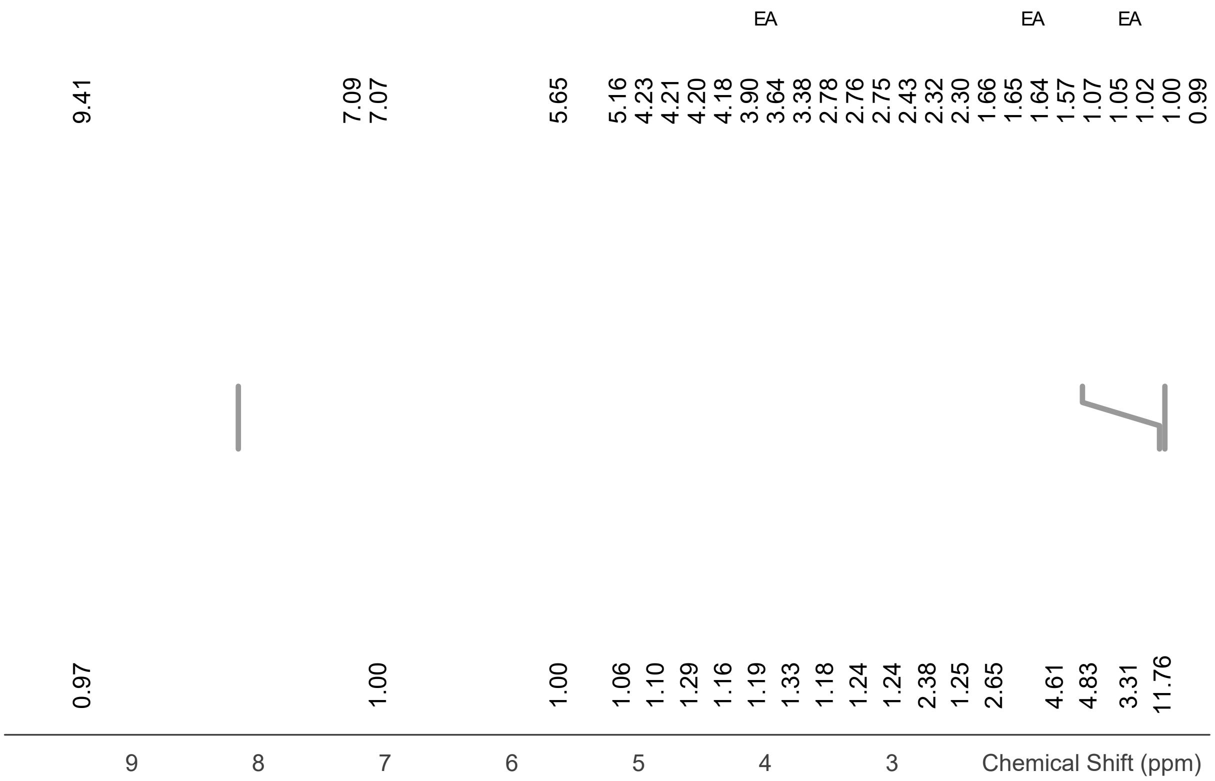

Figure S42: ^{1}H NMR spectrum (500 MHz, $CDCl_3$, 298 K) of the purified compound **5**. EA: ethyl acetate.

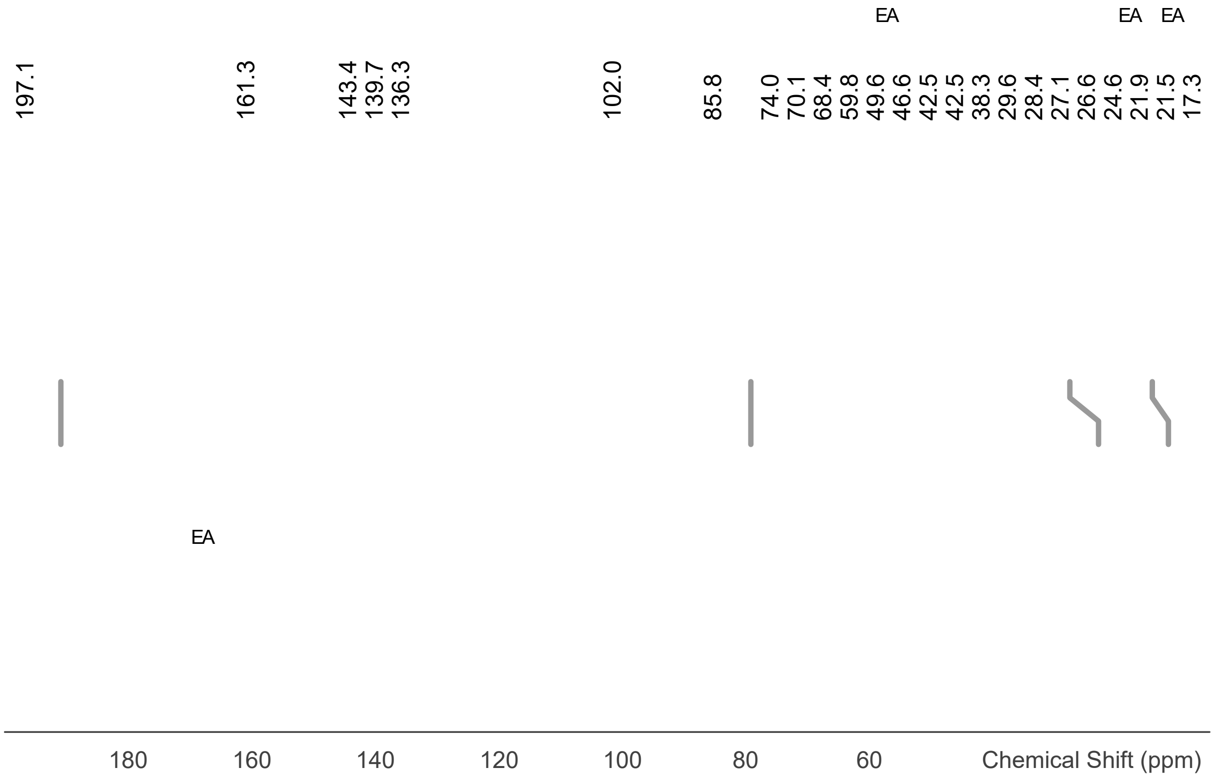

Figure S43: ^{13}C NMR spectrum (125 MHz, $CDCl_3$, 298 K) of the purified compound **5**.

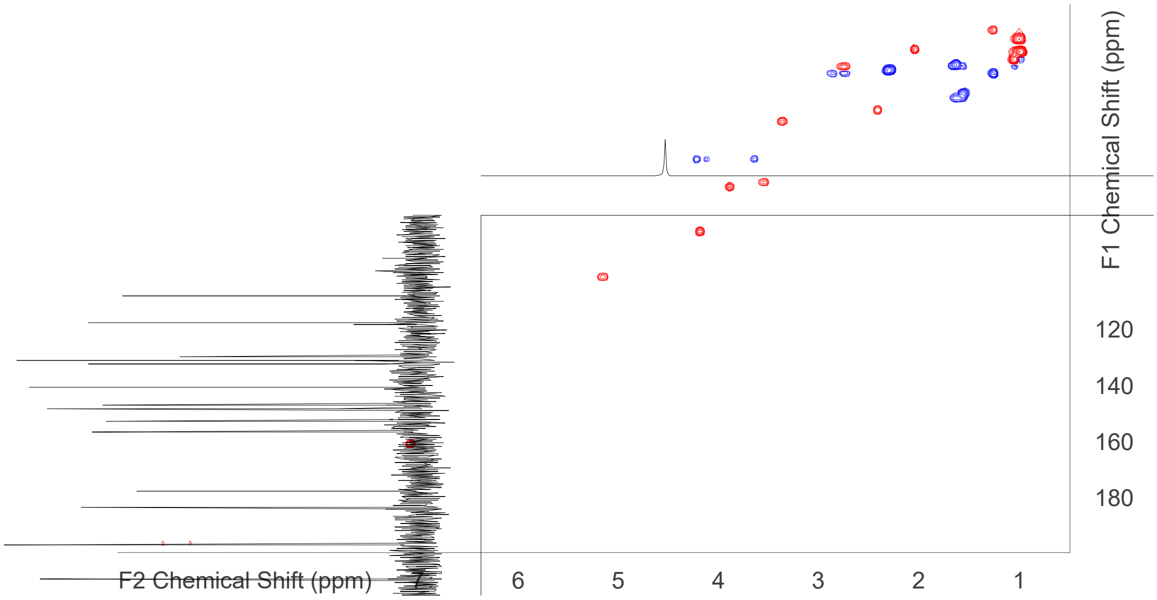

Figure S44: $^{1}H,^{13}C$ HSQC-DEPT spectrum (500 MHz, $CDCl_3$, 298 K) of the purified compound **5**.

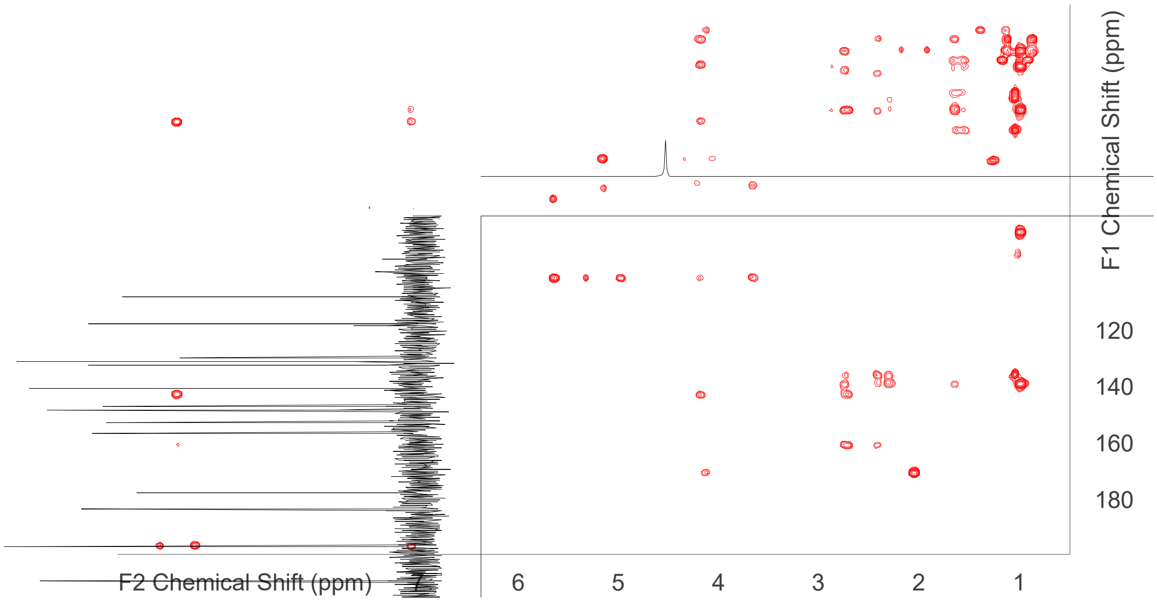

Figure S45: $^{1}H,^{13}C$ HMBC spectrum (500 MHz, $CDCl_3$, 298 K) of the purified compound **5**.

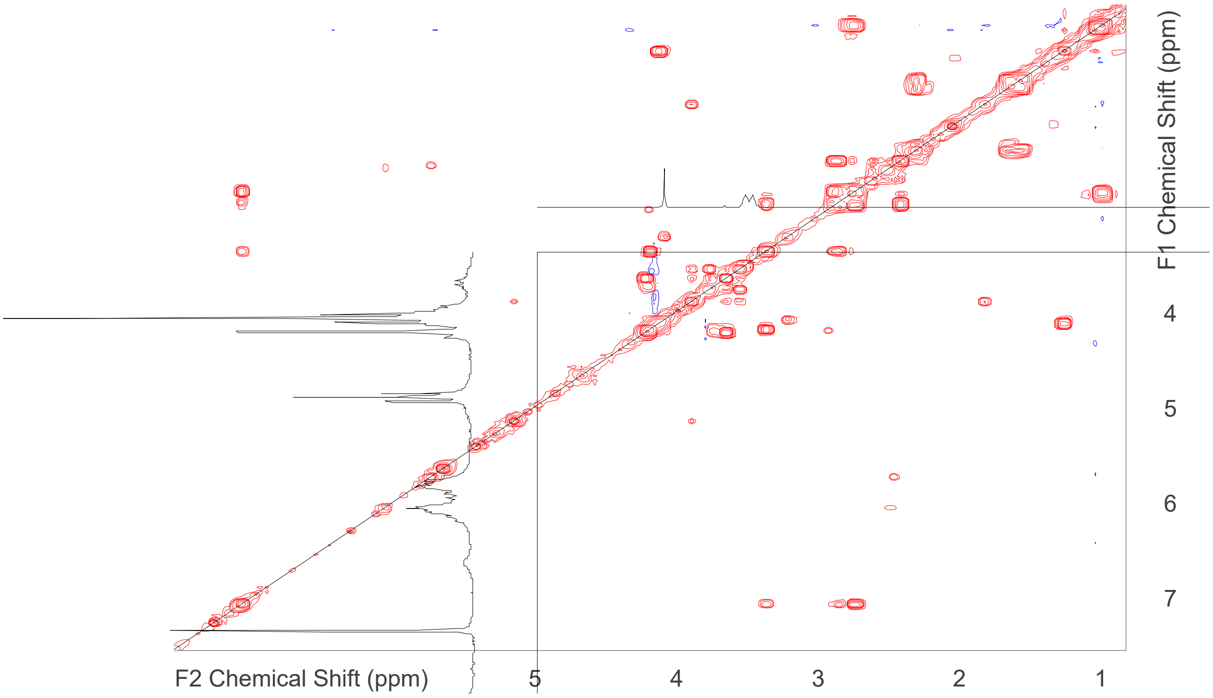

Figure S46: $^{1}H,^{1}H$ COSY spectrum (500 MHz, $CDCl_3$, 298 K) of the purified compound **5**.

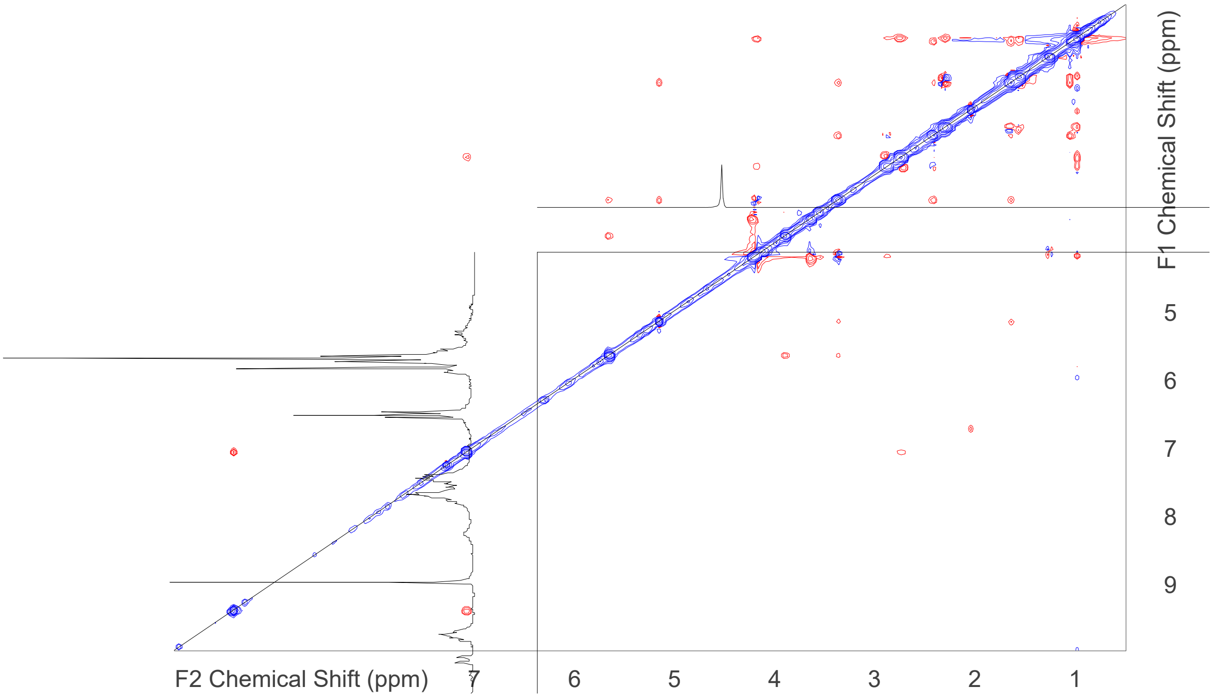

Figure S47: $^{1}H,^{1}H$ ROESY spectrum (500 MHz, $CDCl_3$, 298 K) of the purified compound **5**.

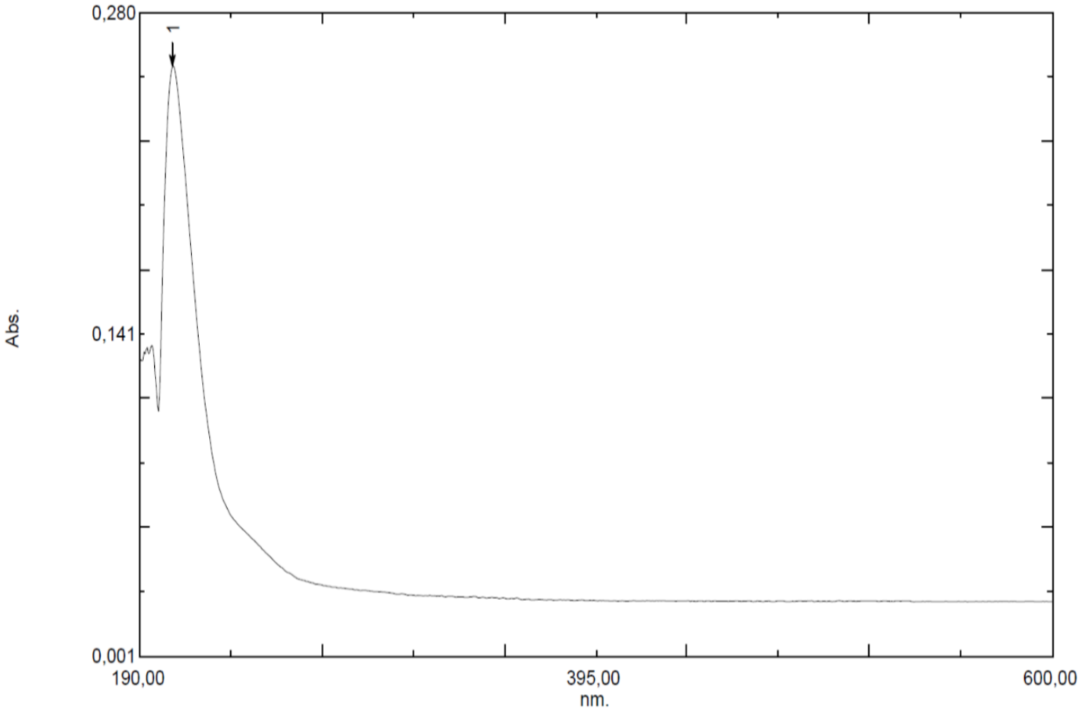

Figure S48: UV/VIS spectrum of compound **5** in MeOH.

(10) Analytical data of compound 6

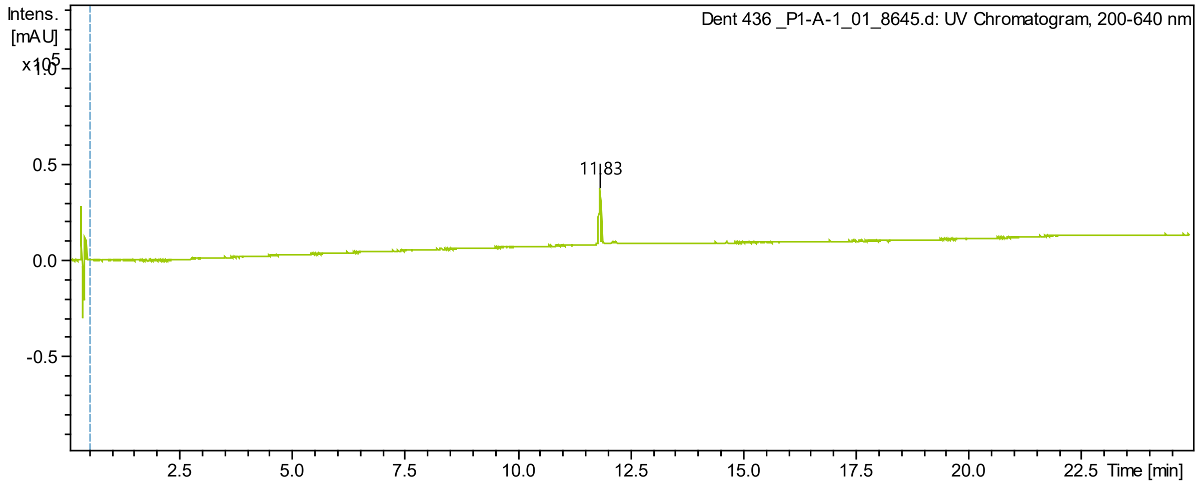

Figure S49: HPLC-DAD chromatogram of compound **6**.

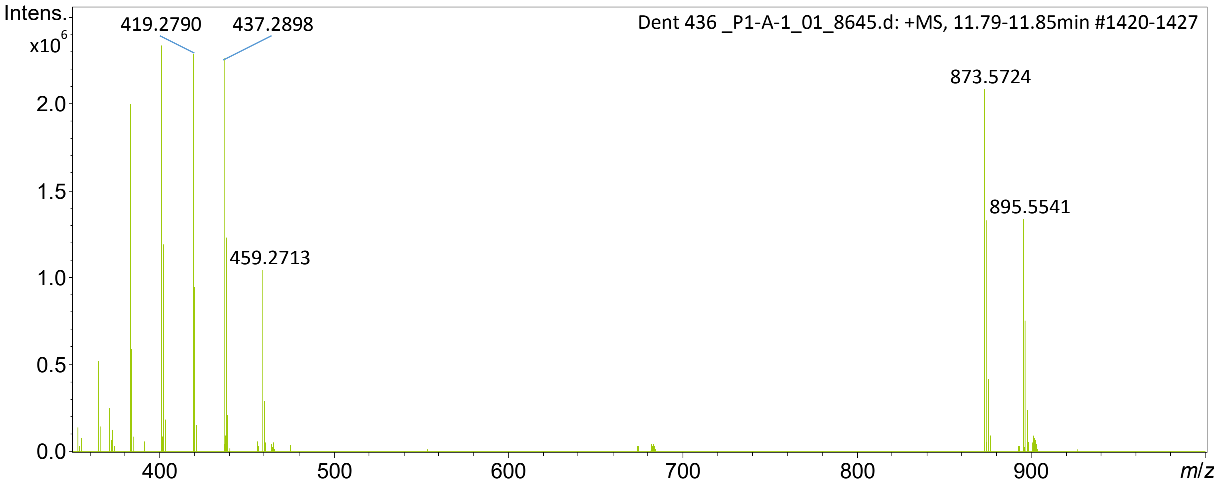

Figure S50: HR-(+)ESIMS spectrum of compound **6**.

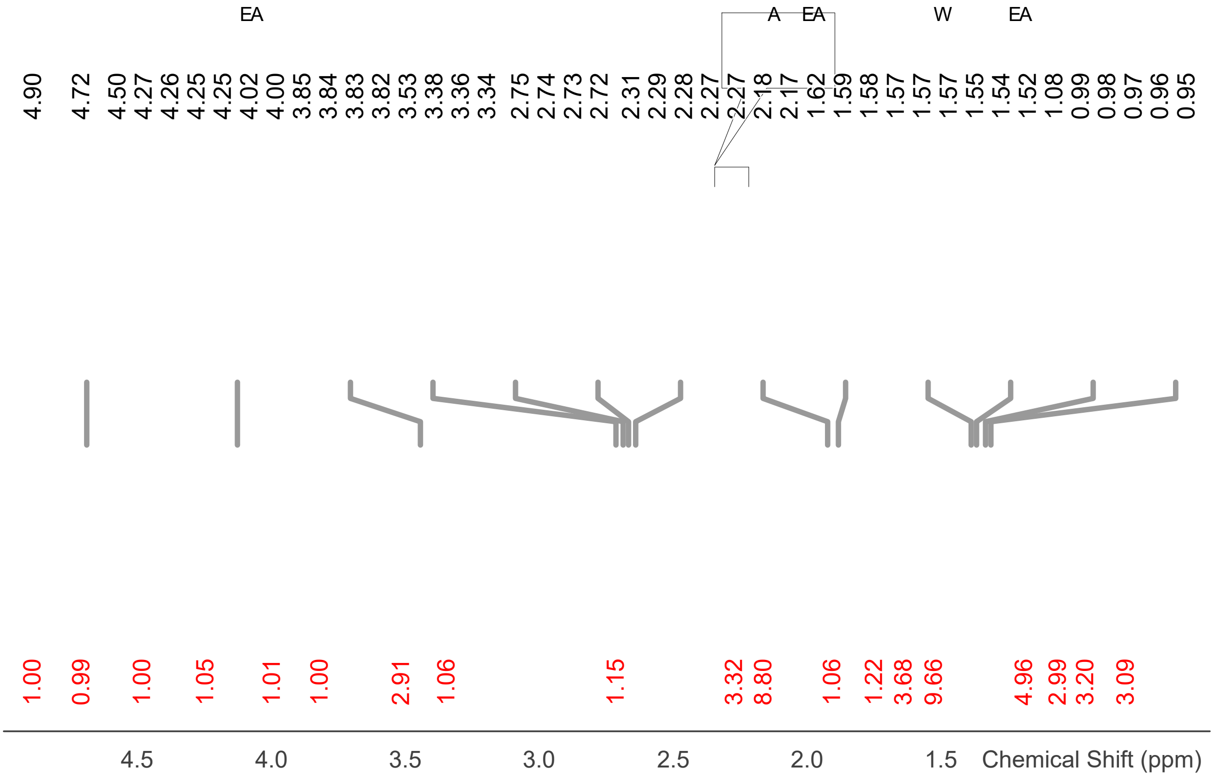

Figure S51: ^{1}H NMR spectrum (500 MHz, $CDCl_3$, 298 K) of the purified compound **6**. W: H_2O, A: acetone, M: methanol.

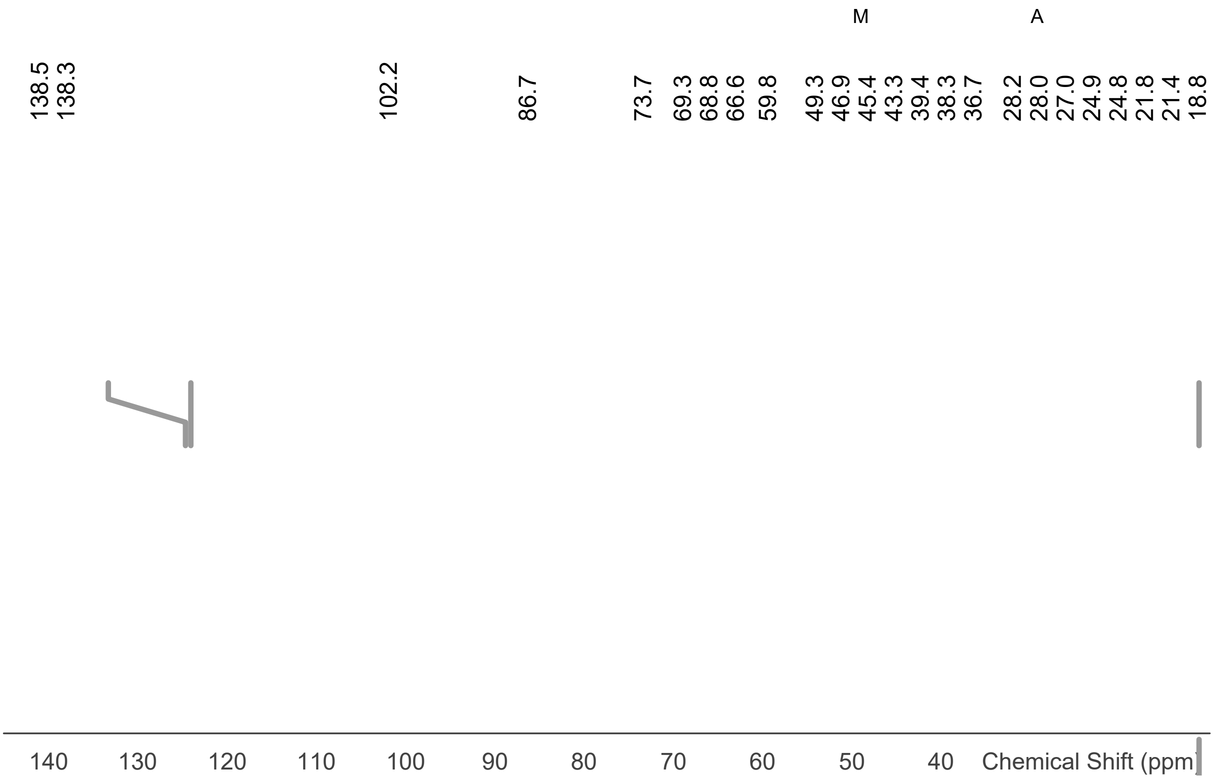

Figure S52: ^{13}C NMR spectrum (125 MHz, $CDCl_3$, 298 K) of the purified compound **6**. A: acetone, M: methanol.

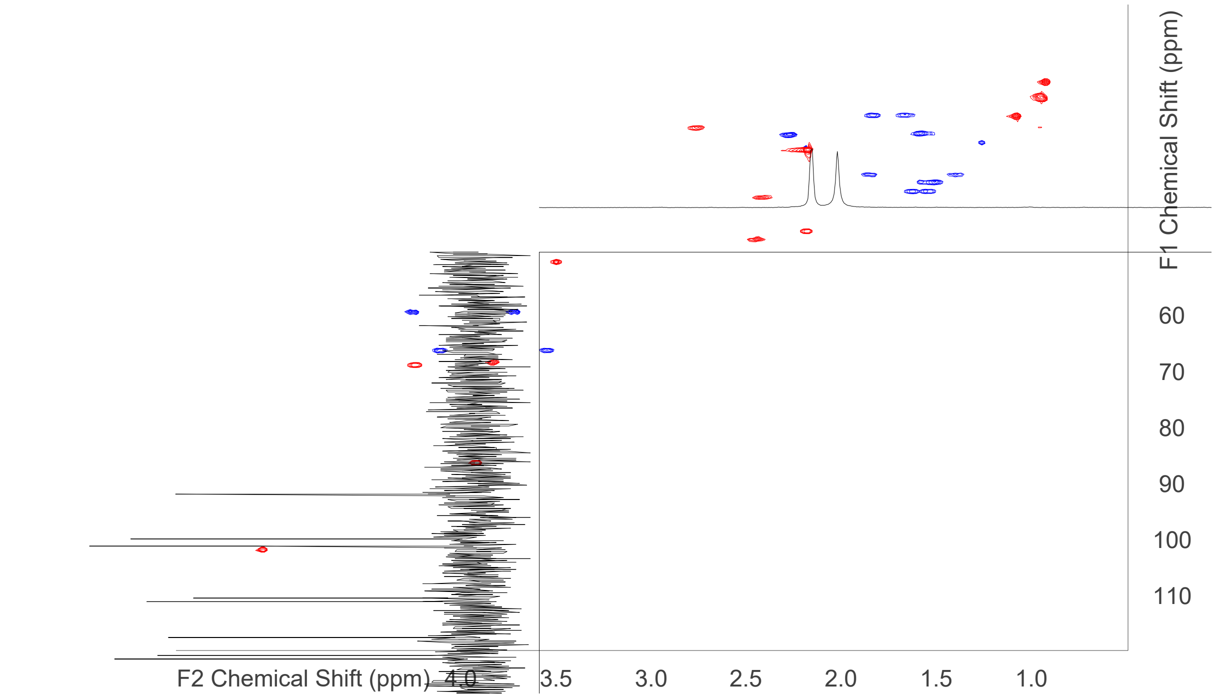

Figure S53: $^{1}H,^{13}C$ HSQC-DEPT spectrum (500 MHz, $CDCl_3$, 298 K) of the purified compound **6**.

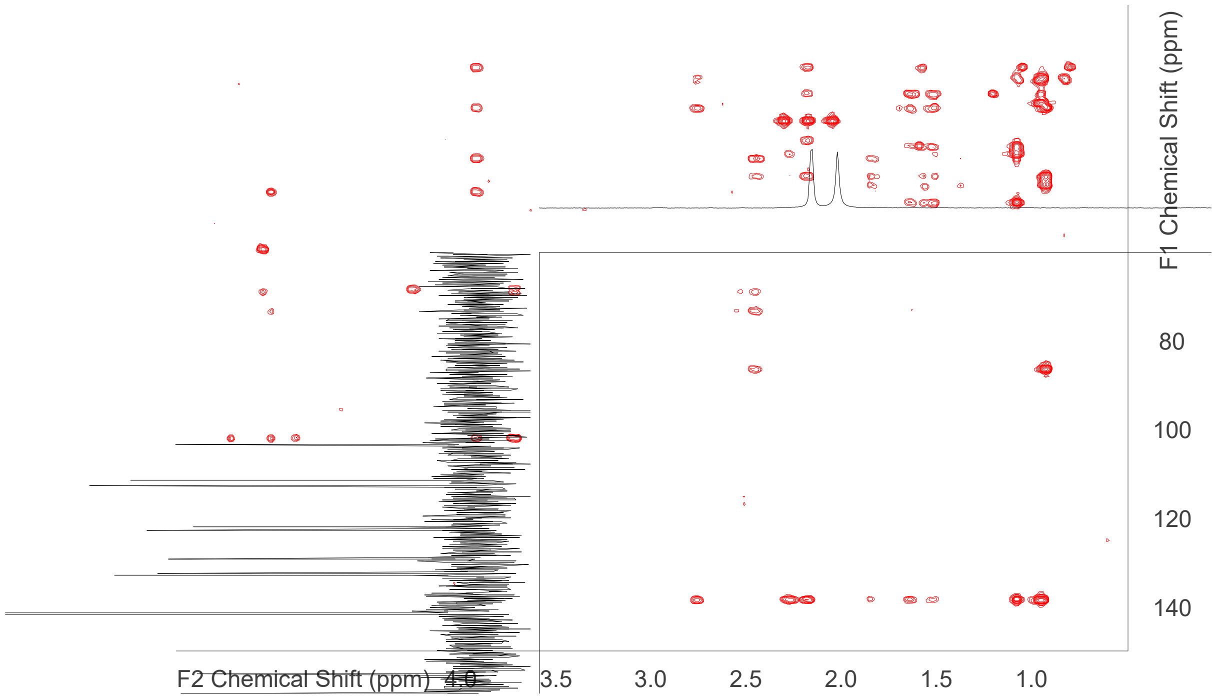

Figure S54: $^{1}H,^{13}C$ HMBC spectrum (500 MHz, $CDCl_3$, 298 K) of the purified compound **6**.

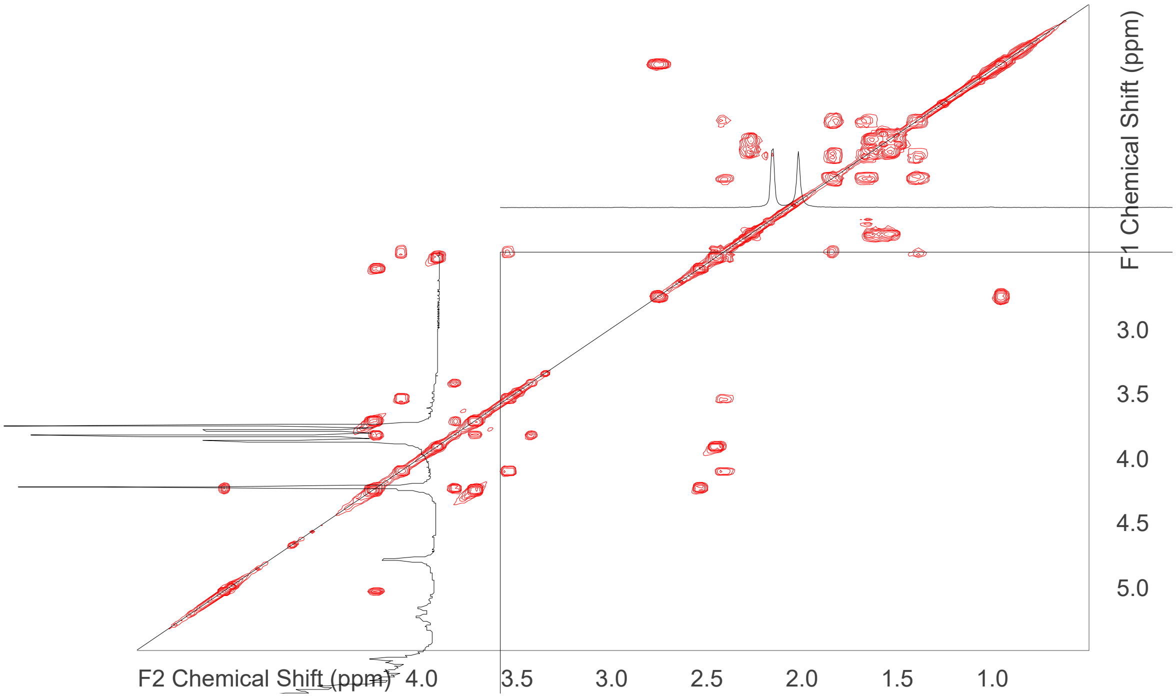

Figure S55: $^1H,^1H$ COSY spectrum (500 MHz, $CDCl_3$, 298 K) of the purified compound **6**.

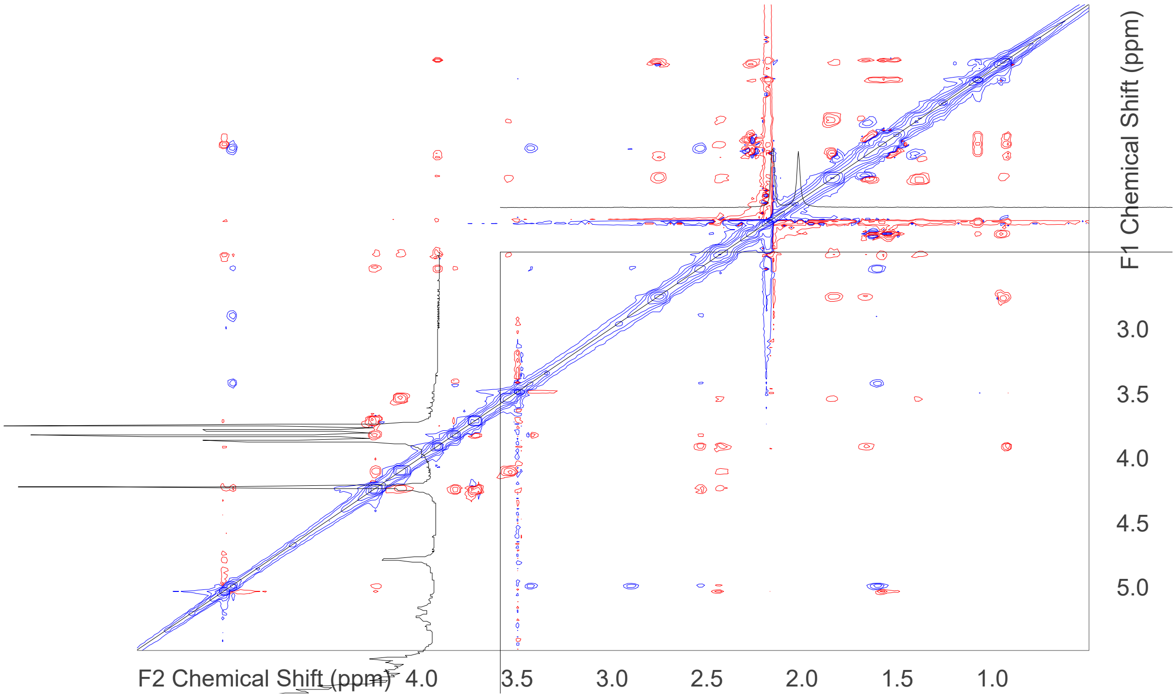

Figure S56: $^1H,^1H$ ROESY spectrum (500 MHz, $CDCl_3$, 298 K) of the purified compound **6**.

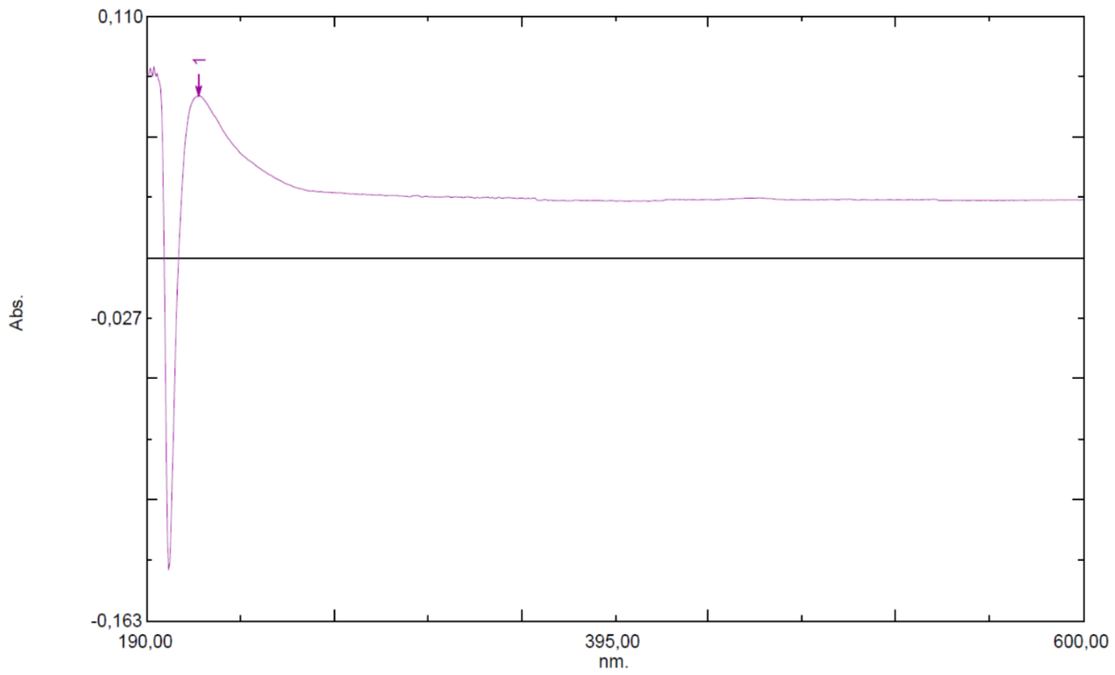

Figure S57: UV/VIS spectrum of compound **6** in MeOH.

(11) Analytical data of compound 7

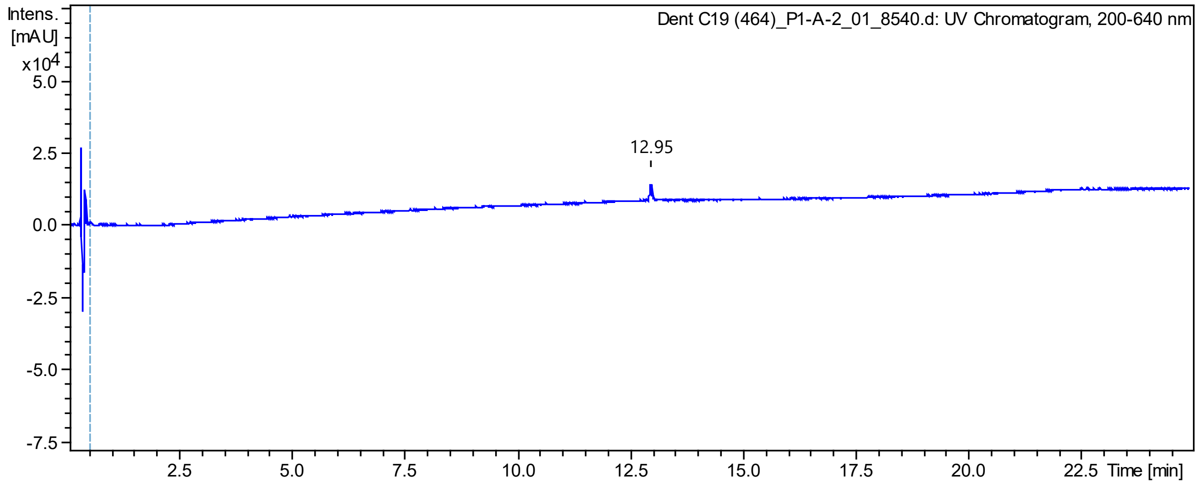

Figure S58: HPLC-DAD chromatogram of compound **7**.

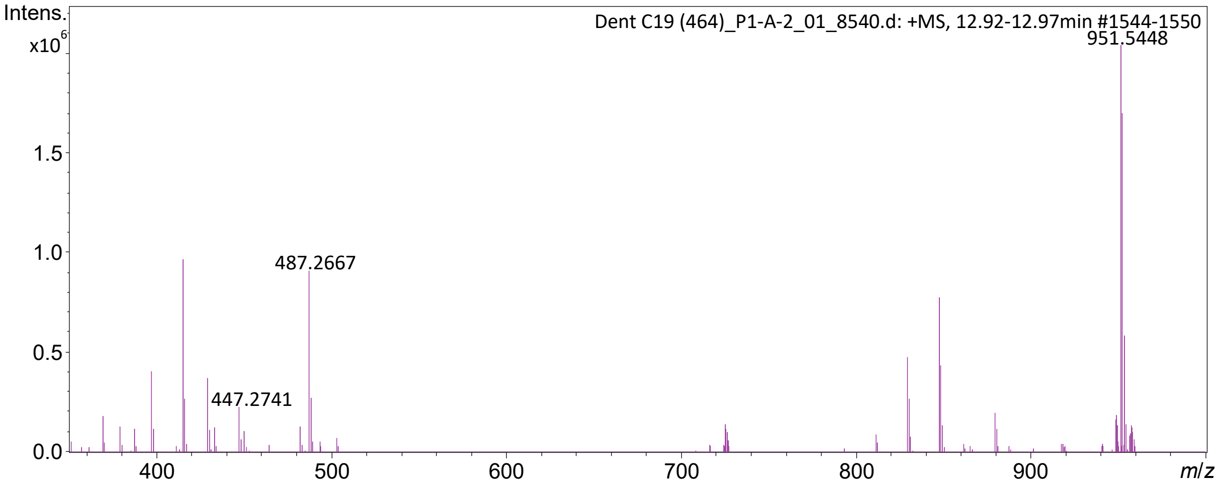

Figure S59: HR-(+)ESIMS spectrum of compound **7**.

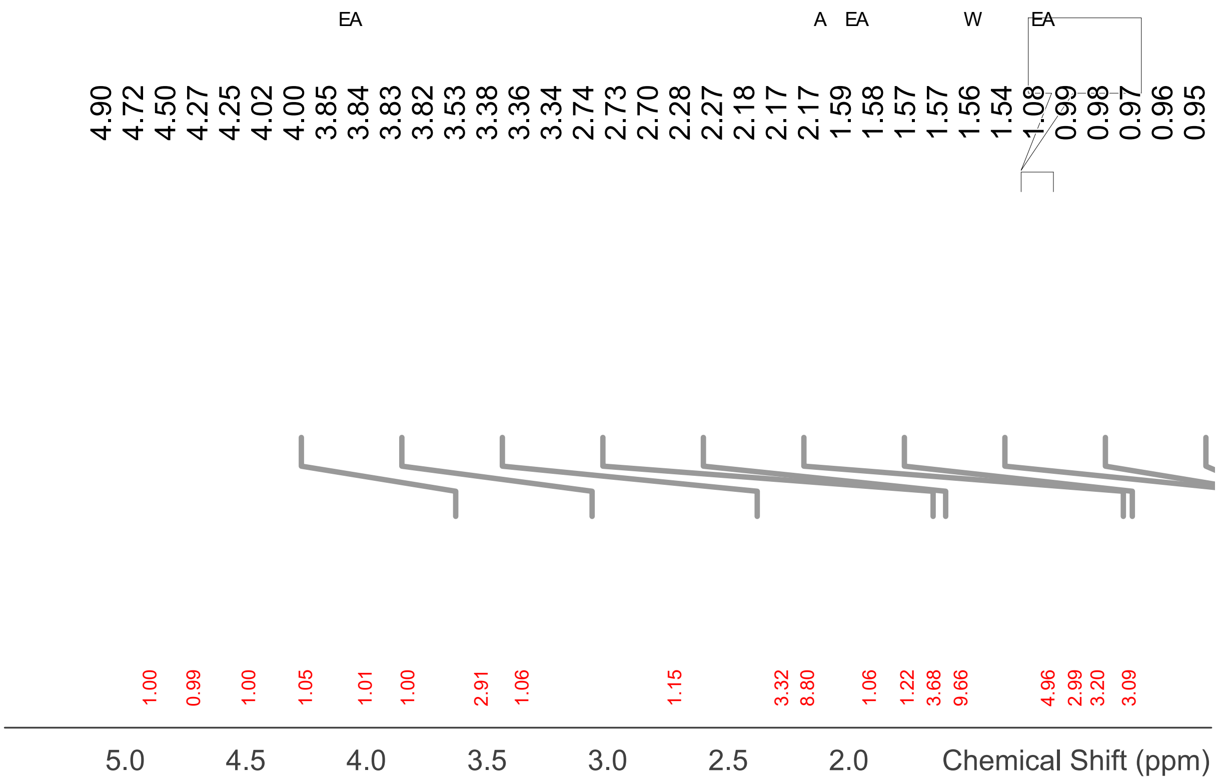

Figure S60: ^{1}H NMR spectrum (700 MHz, $CDCl_3$, 298 K) of the purified compound **7**. W: H_2O, EA: ethyl acetate, A: acetone.

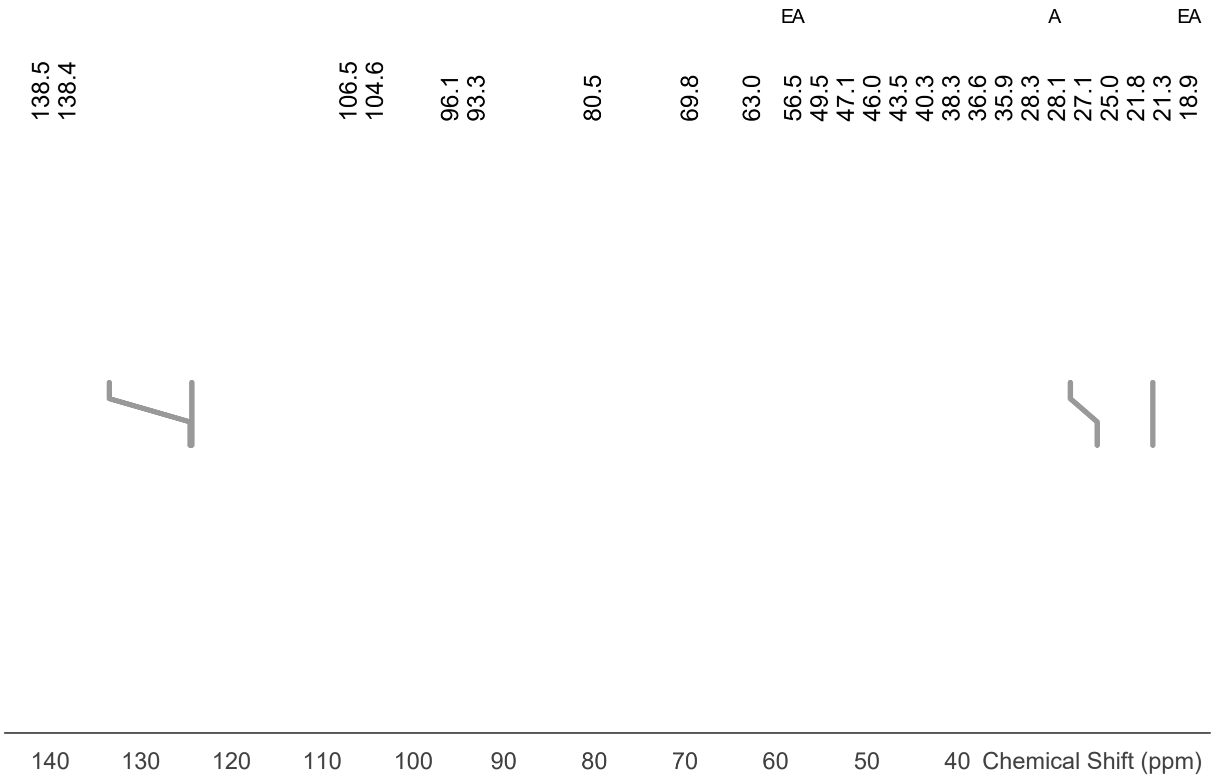

Figure S61: ^{13}C NMR spectrum (175 MHz, $CDCl_3$, 298 K) of the purified compound **7**. EA: ethyl acetate, A: acetone.

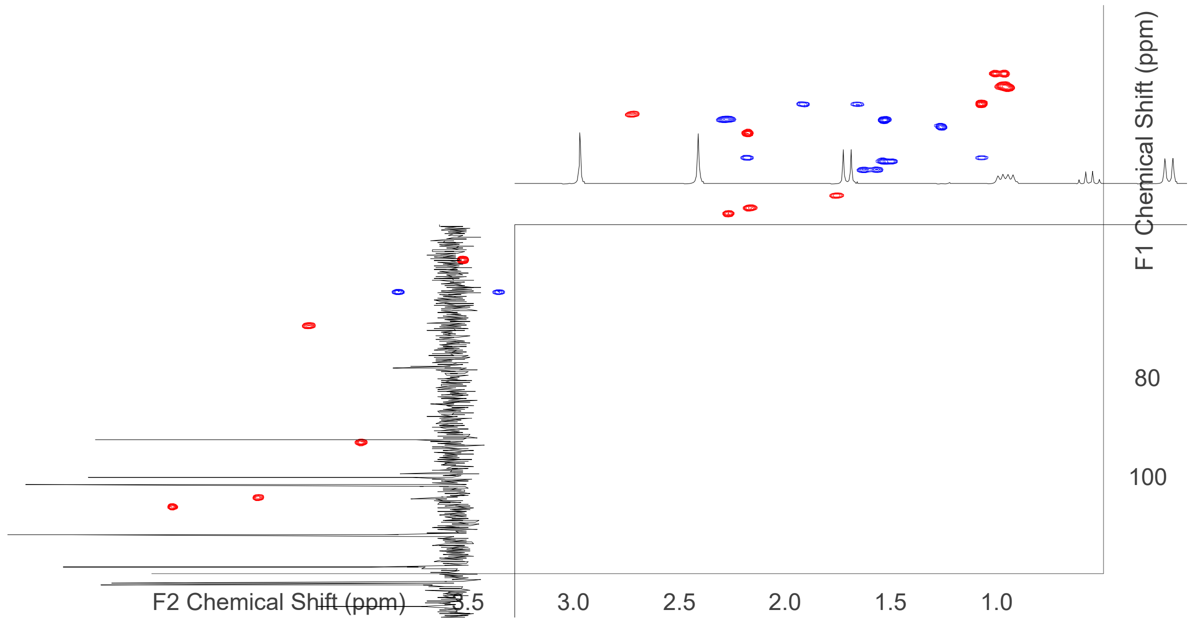

Figure S62: ^{1}H,^{13}C HSQC-DEPT spectrum (700 MHz, $CDCl_3$, 298 K) of the purified compound **7**.

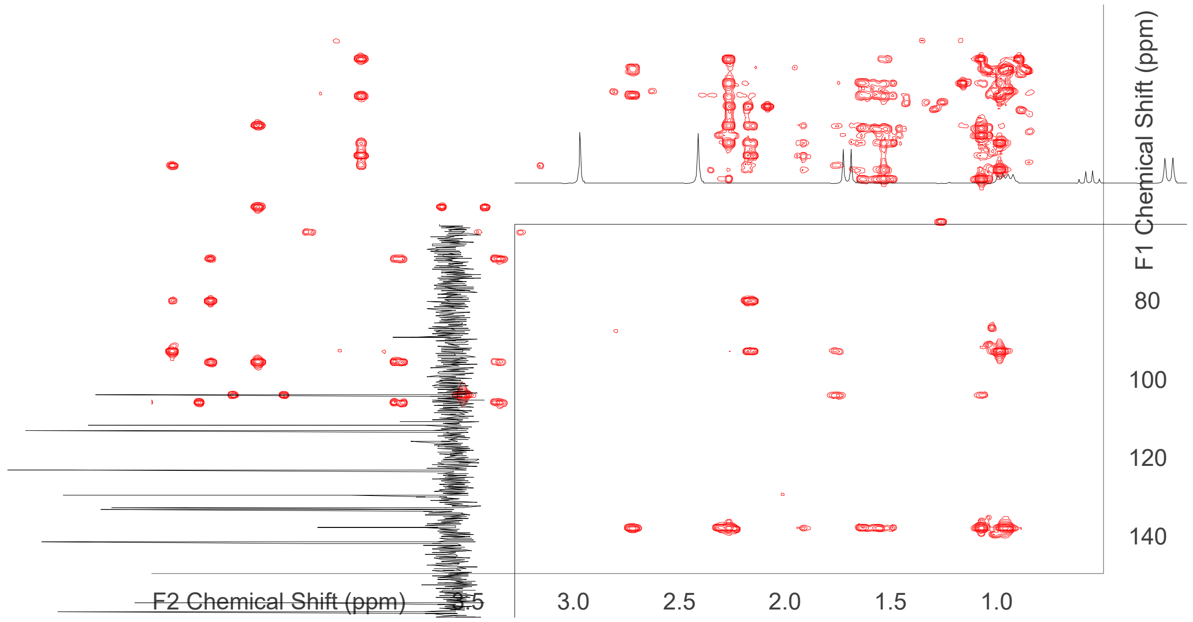

Figure S63: ^{1}H,^{13}C HMBC spectrum (700 MHz, $CDCl_3$, 298 K) of the purified compound **7**.

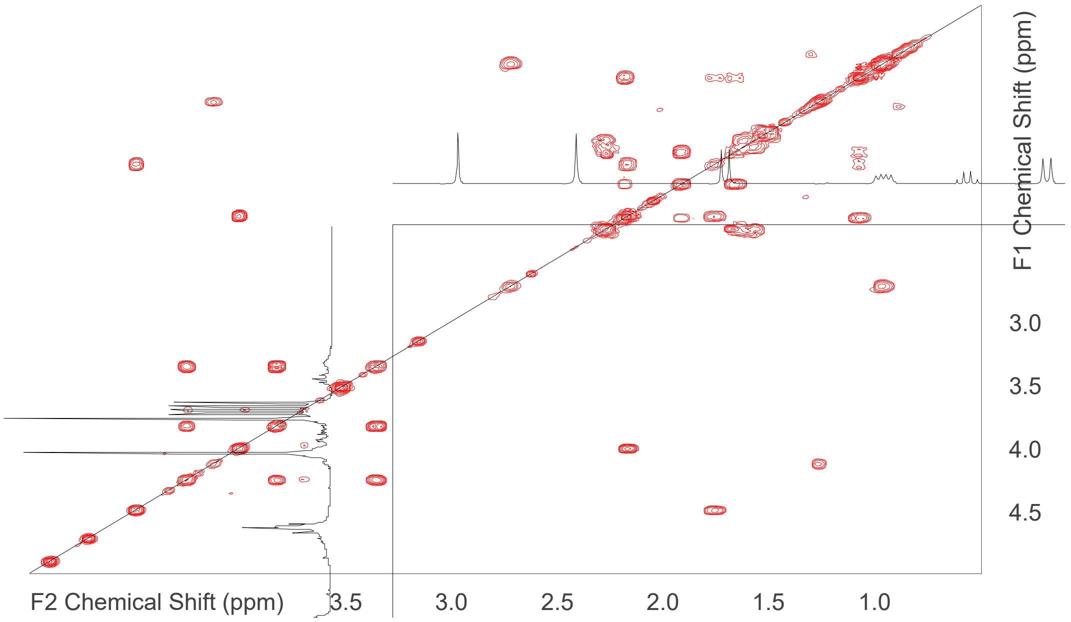

Figure S64: $^{1}H,^{1}H$ COSY spectrum (700 MHz, $CDCl_3$, 298 K) of the purified compound **7**.

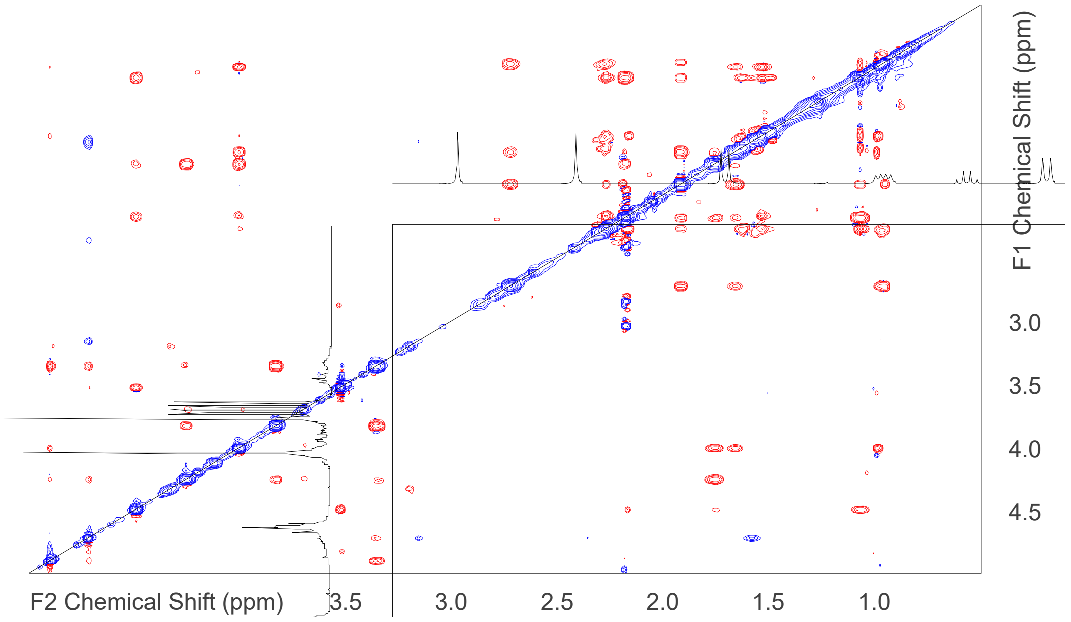

Figure S65: $^{1}H,^{1}H$ ROESY spectrum (700 MHz, $CDCl_3$, 298 K) of the purified compound **7**.

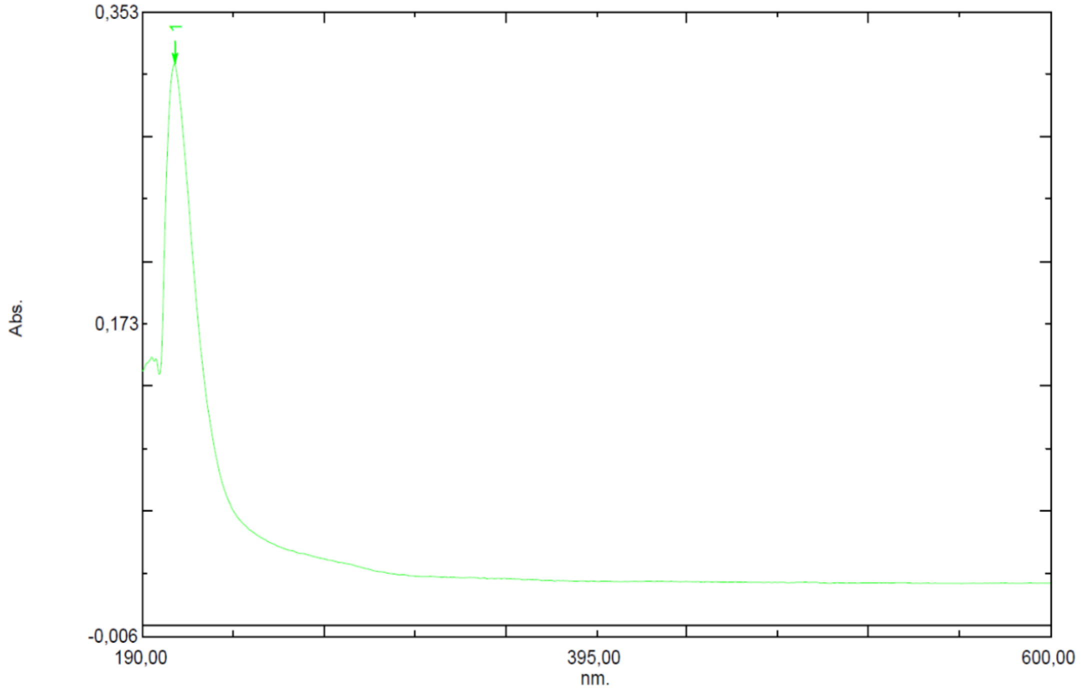

Figure S66: UV/VIS spectrum of compound **7** in MeOH.

(12) Analytical data of compound 8

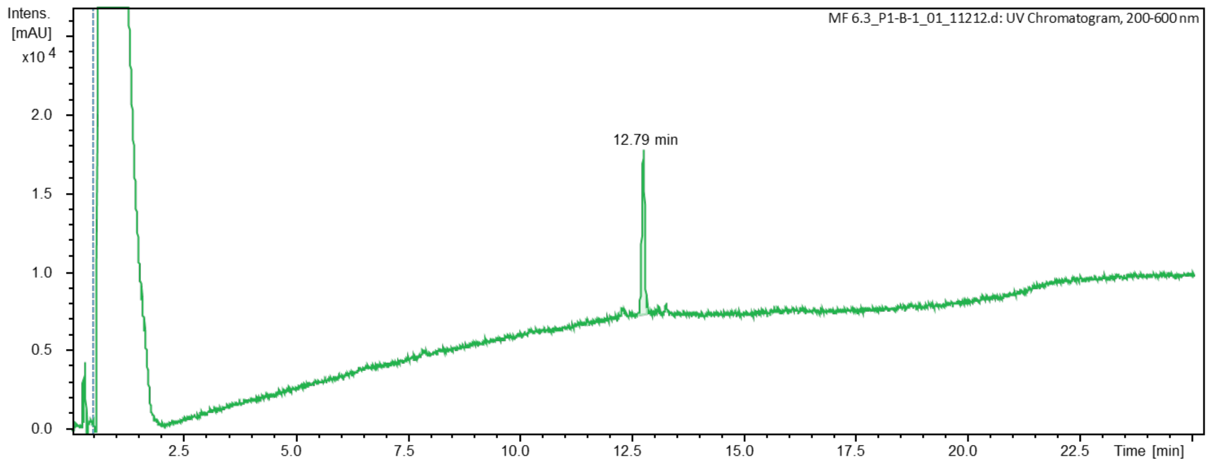

Figure S67: HPLC-DAD chromatogram of compound **8**.

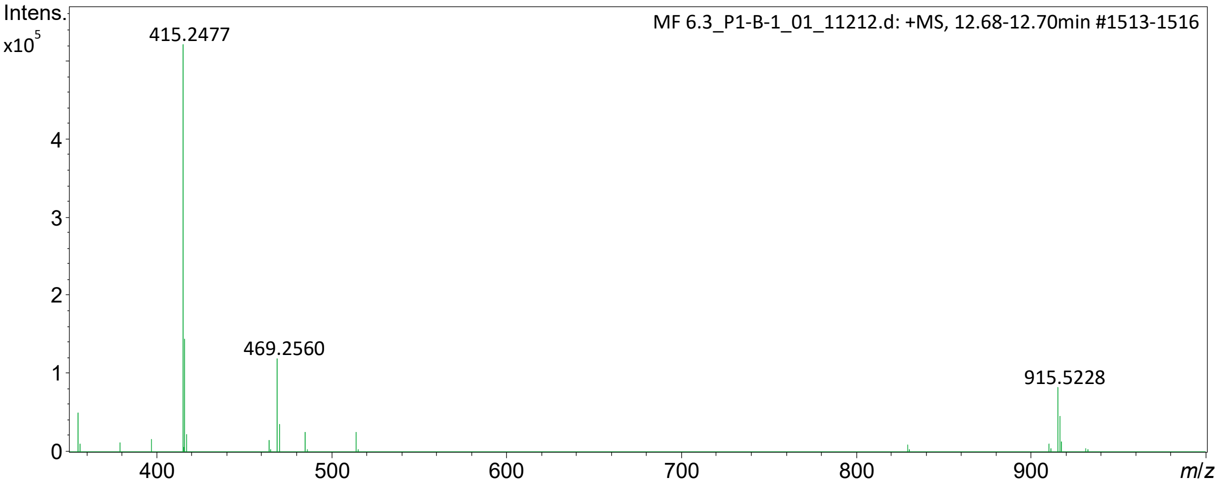

Figure S68: HR-(+)ESIMS spectrum of compound **8**.

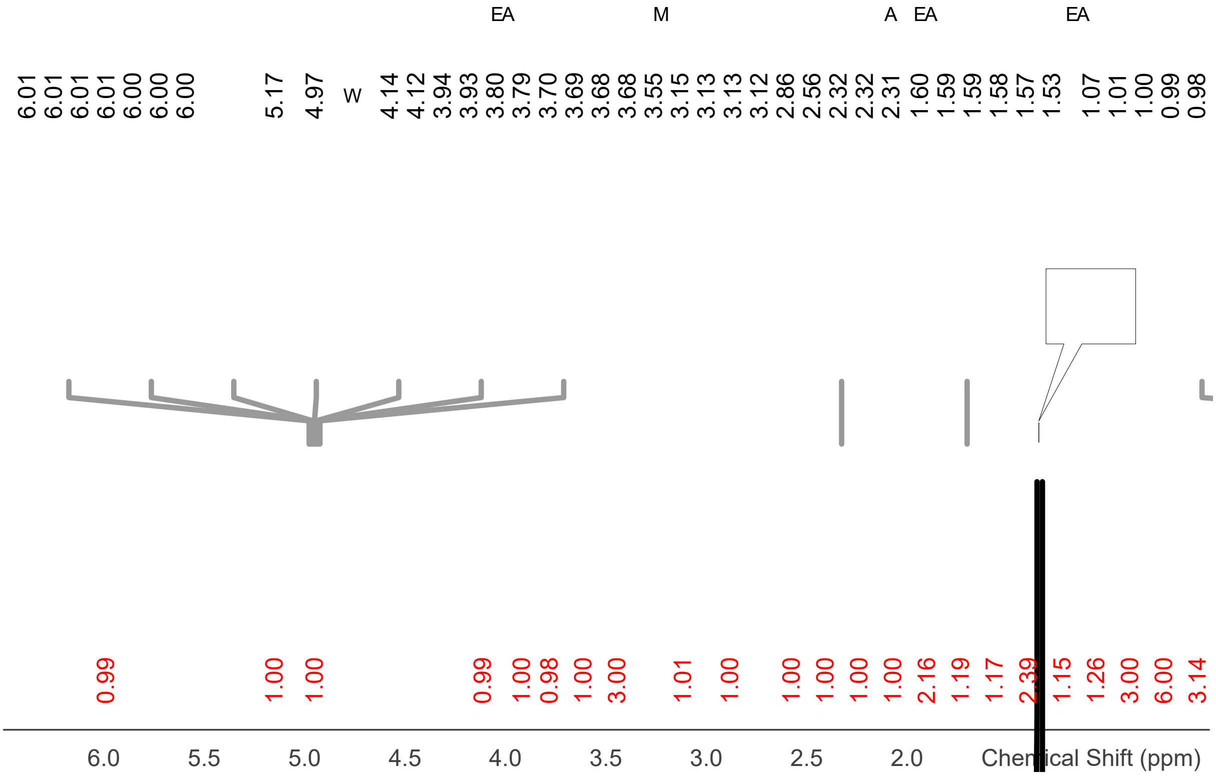

Figure S69: ^{1}H NMR spectrum (700 MHz, CD_3OD, 298 K) of the purified compound **8**. W: H_2O, EA: ethyl acetate, A: acetone, M: methanol.

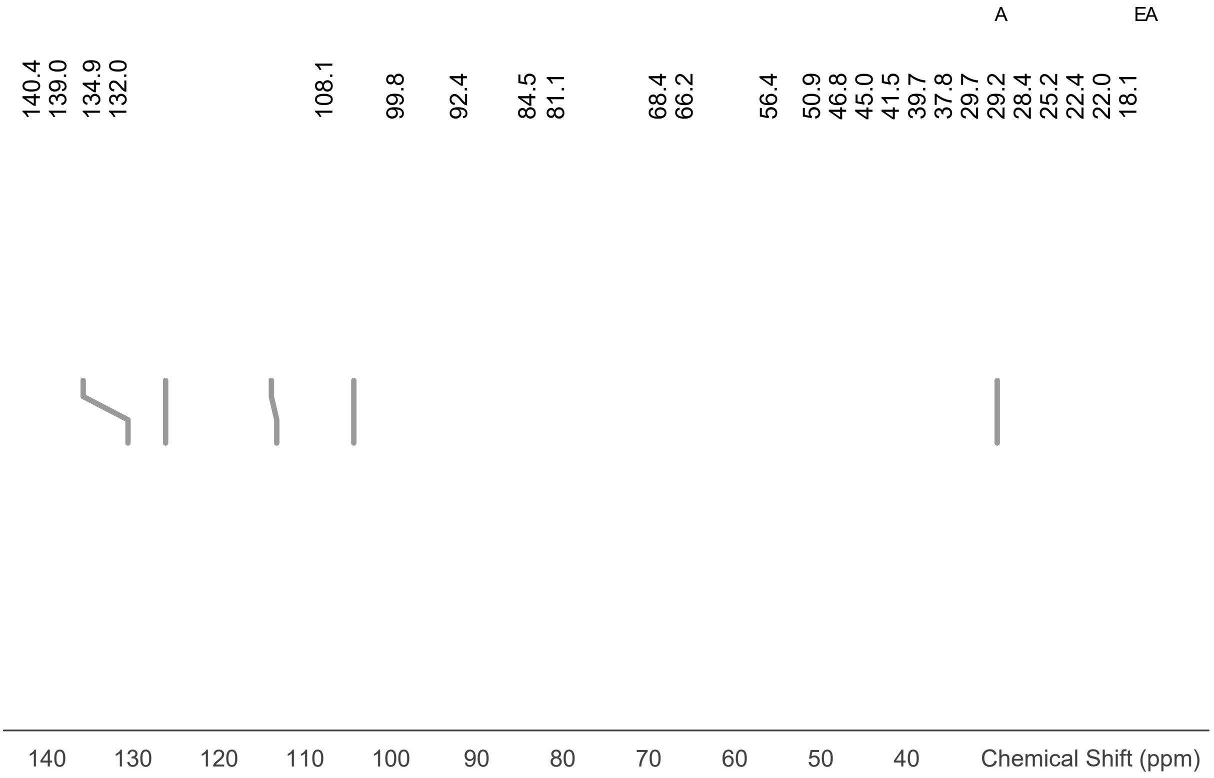

Figure S70: ^{13}C NMR spectrum (175 MHz, CD_3OD, 298 K) of the purified compound **8**. EA: ethyl acetate, A: acetone.

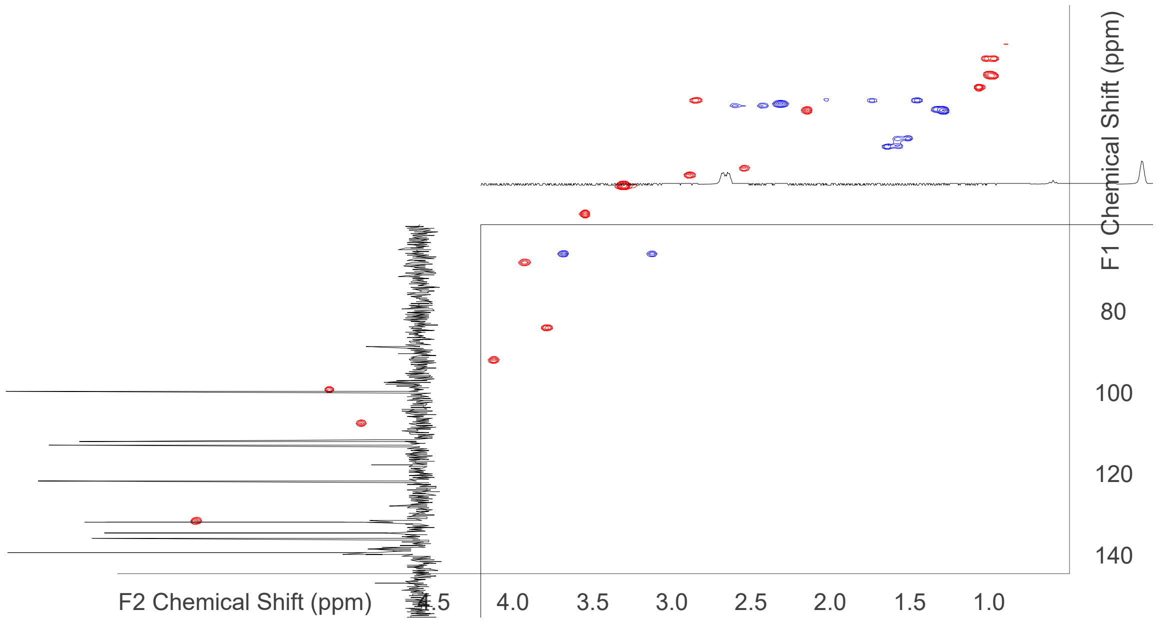

Figure S71: ^{1}H,^{13}C HSQC-DEPT spectrum (700 MHz, CD_3OD, 298 K) of the purified compound **8**.

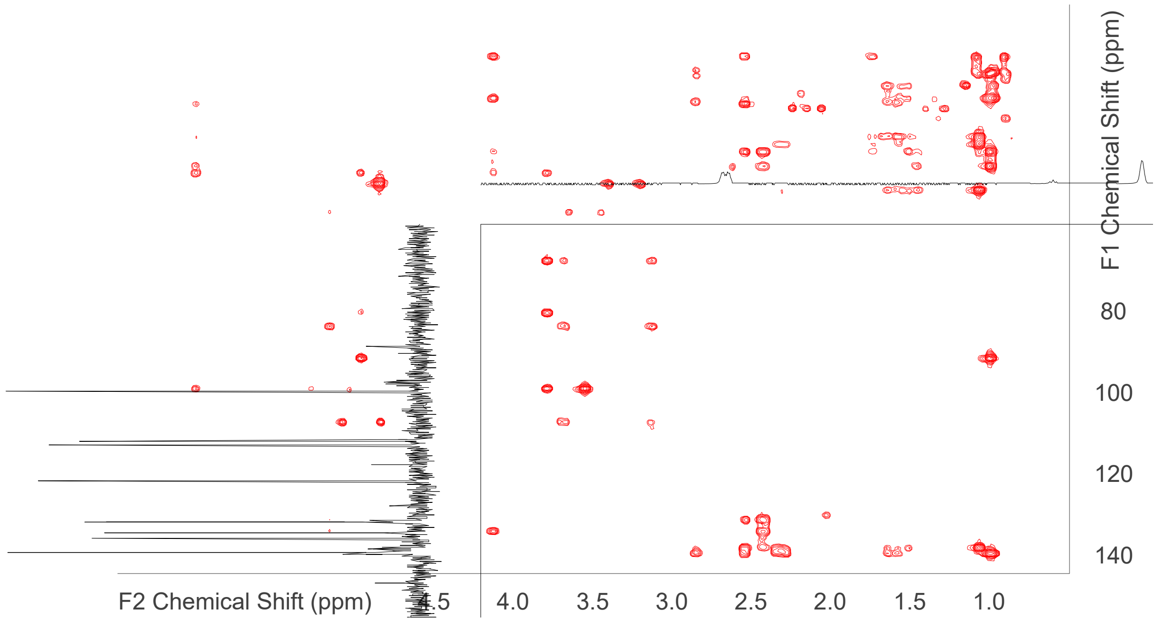

Figure S72: ^{1}H,^{13}C HMBC spectrum (700 MHz, CD_3OD, 298 K) of the purified compound **8**.

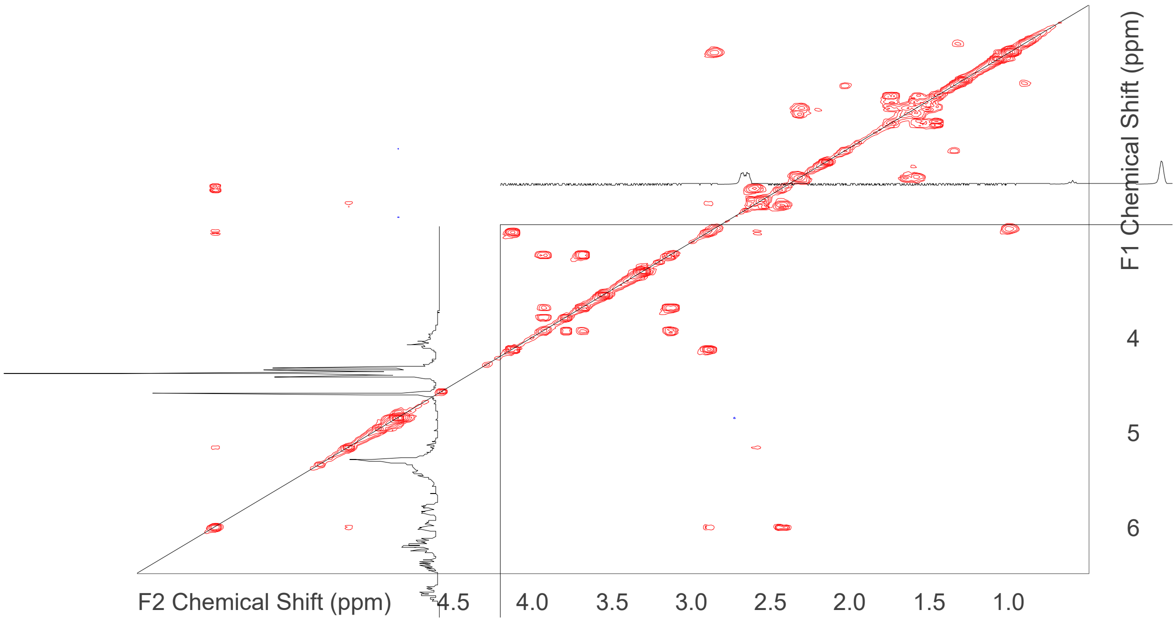

Figure S73: ^{1}H,^{1}H COSY spectrum (700 MHz, CD_3OD, 298 K) of the purified compound **8**.

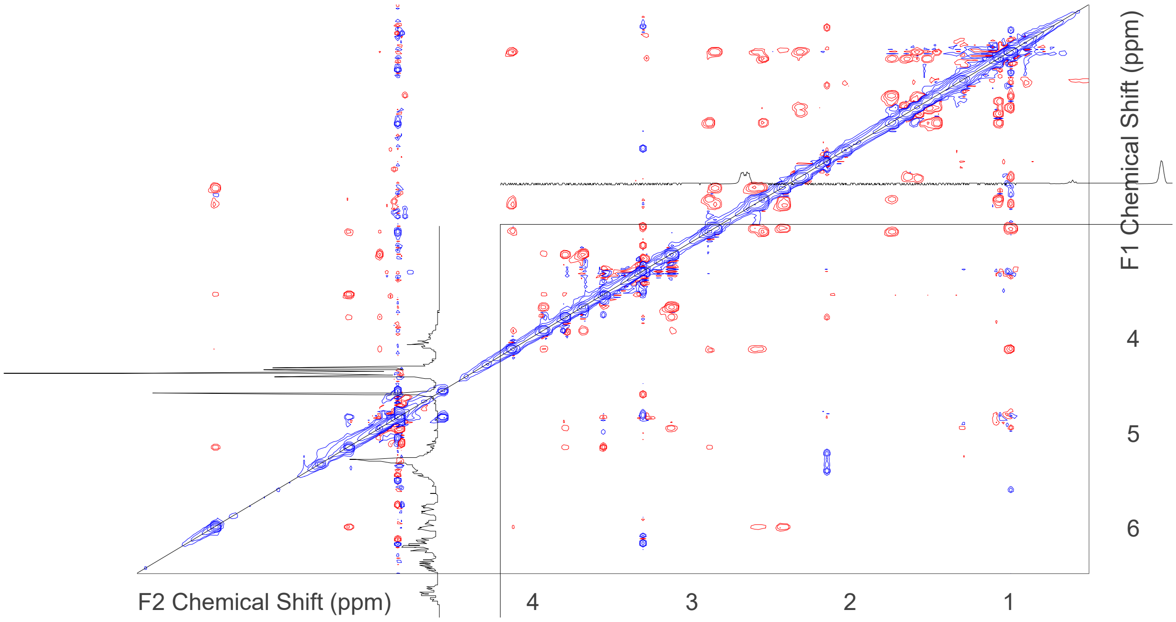

Figure S74: ^{1}H,^{1}H ROESY spectrum (700 MHz, CD_3OD, 298 K) of the purified compound **8**.

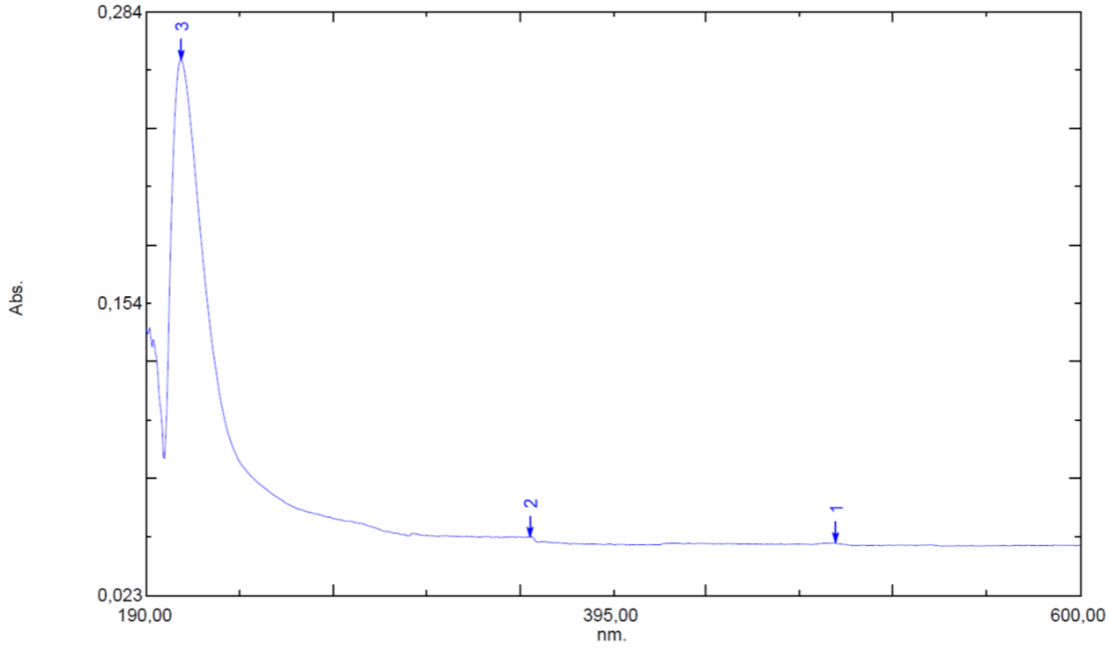

Figure S75: UV/VIS spectrum of compound **8** in MeOH.

(13) Analytical data of compound 9

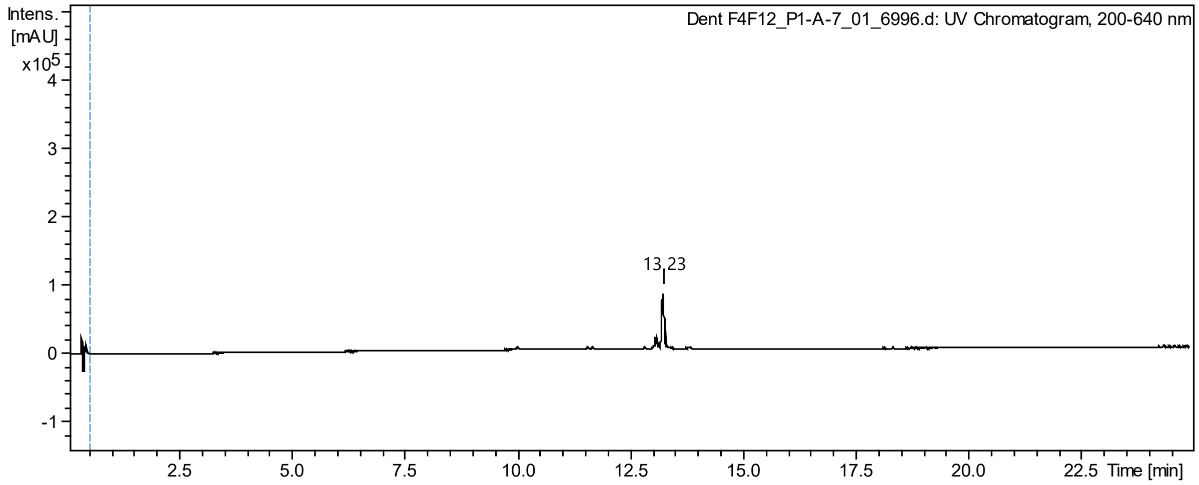

Figure S76: HPLC-DAD chromatogram of compound **9**.

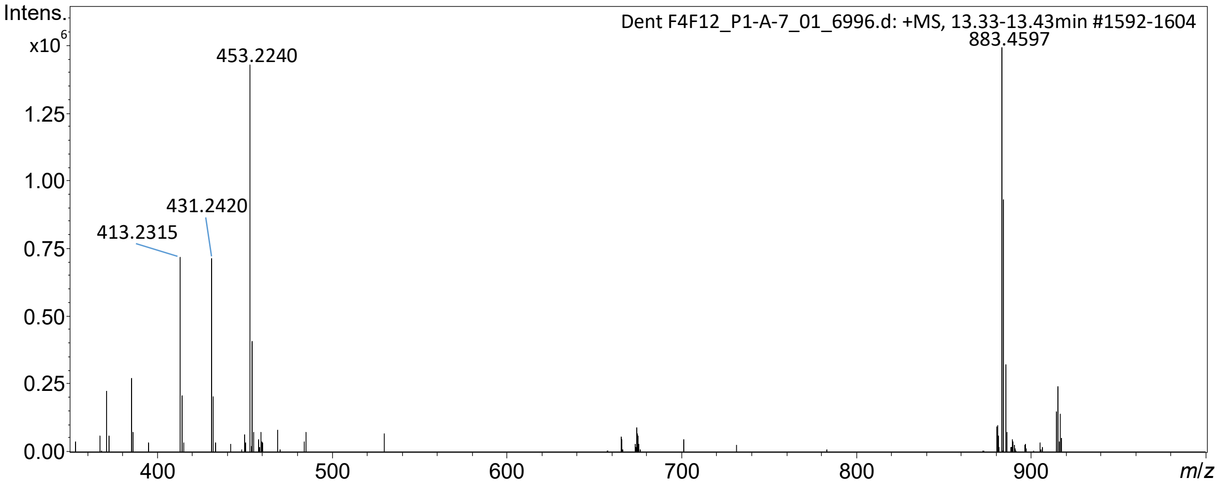

Figure S77: HR-(+)ESIMS spectrum of compound **9**.

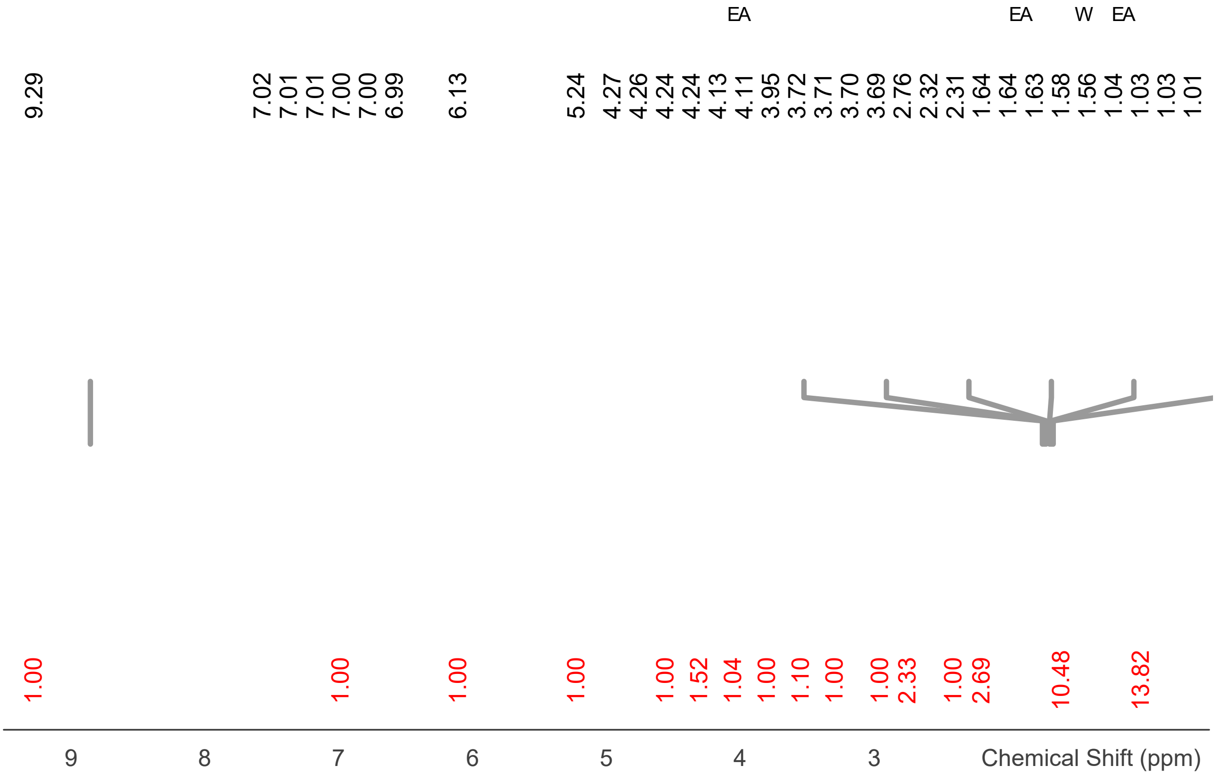

Figure S78: ^{1}H NMR spectrum (500 MHz, $CDCl_3$, 298 K) of the purified striatal D (**9**). W: H_2O, EA: ethyl acetate.

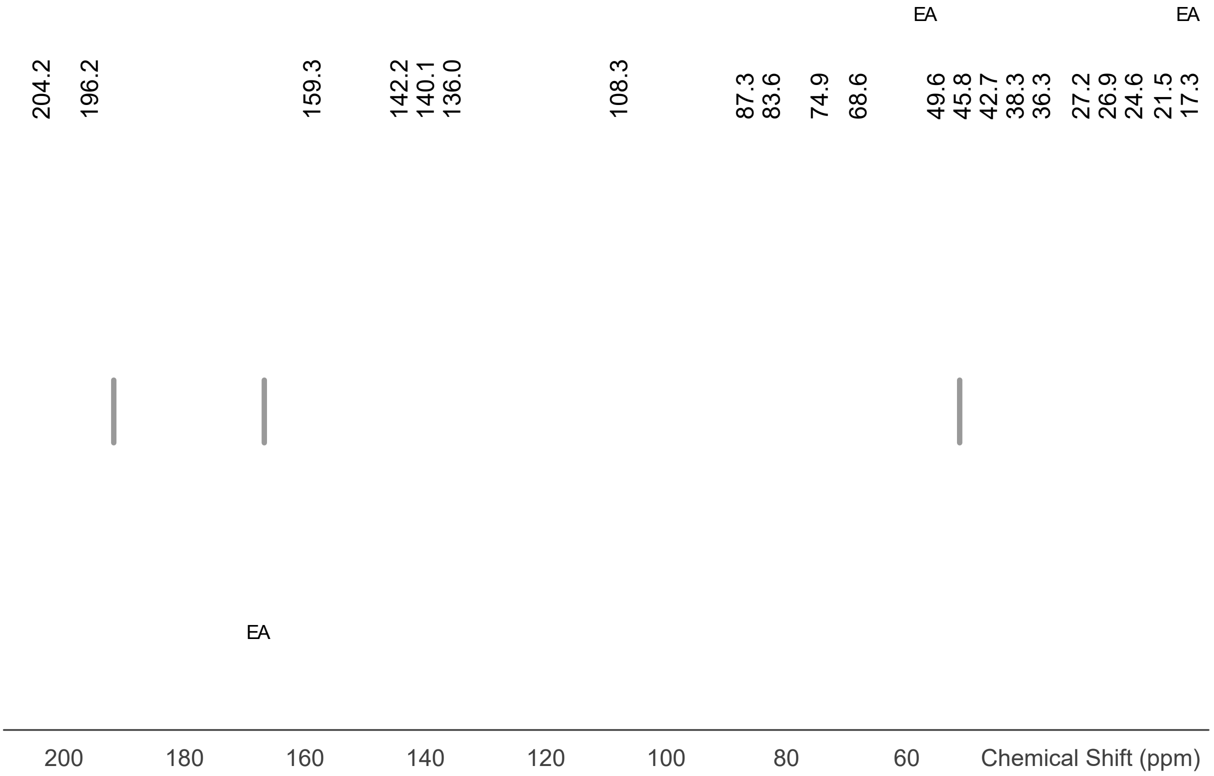

Figure S79: ^{13}C NMR spectrum (125 MHz, $CDCl_3$, 298 K) of the purified striatal D (**9**). EA: ethyl acetate.

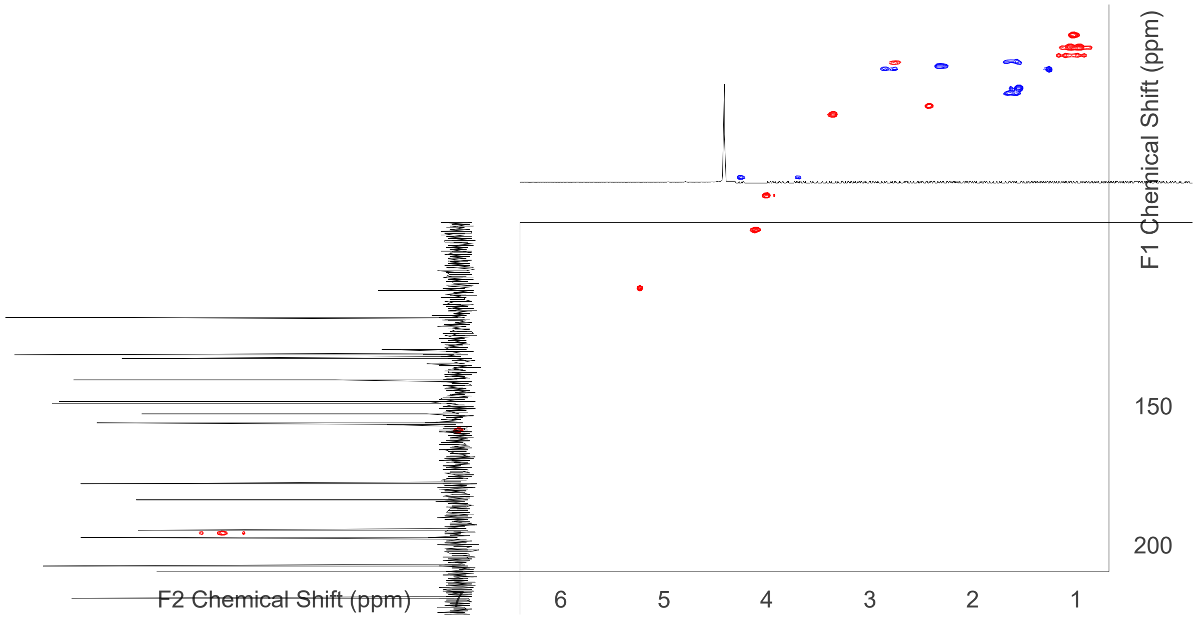

Figure S80: $^{1}H,^{13}C$ HSQC-DEPT spectrum (500 MHz, $CDCl_3$, 298 K) of the purified striatal D (**9**).

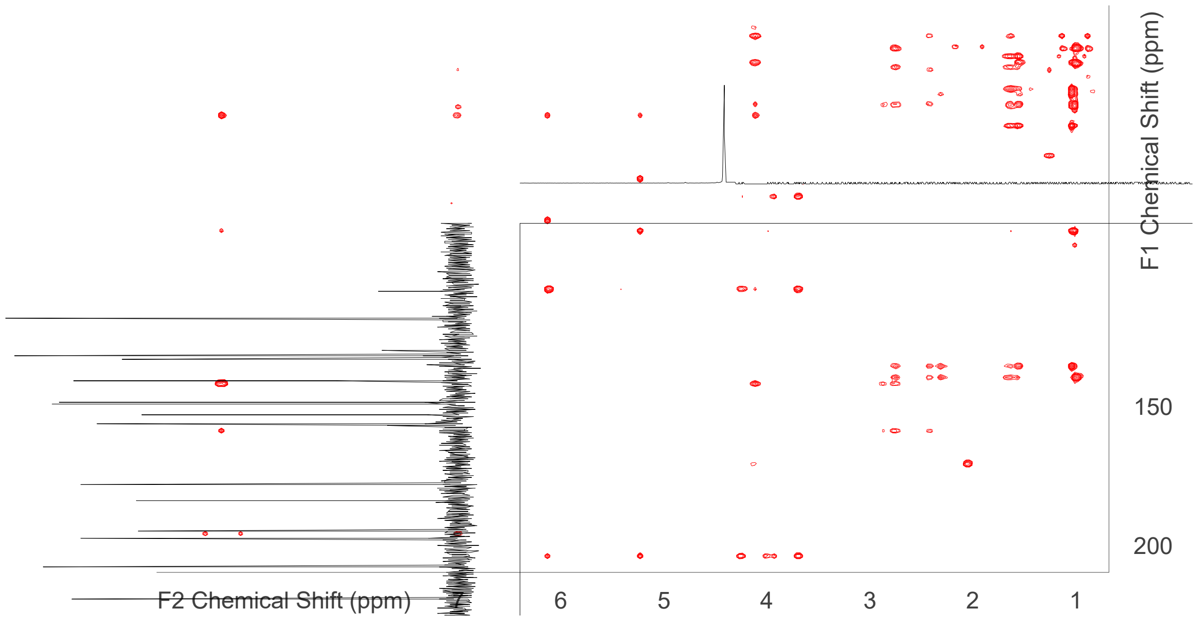

Figure S81: $^{1}H,^{13}C$ HMBC spectrum (500 MHz, $CDCl_3$, 298 K) of the purified striatal D (**9**).

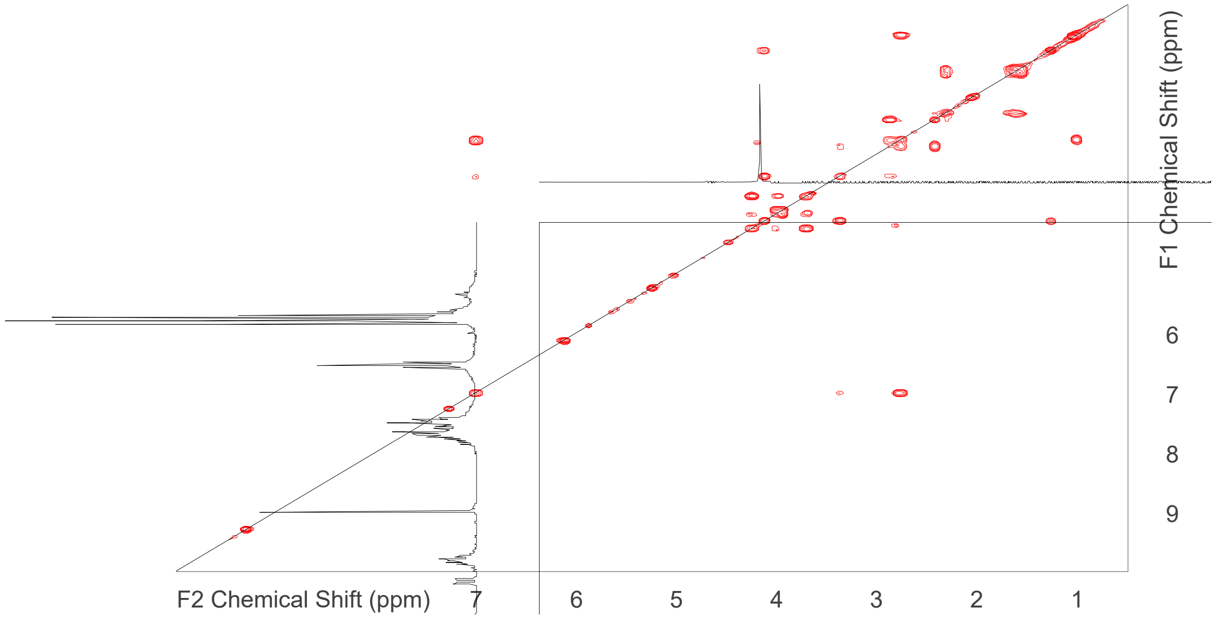

Figure S82: ^{1}H,^{1}H COSY spectrum (500 MHz, $CDCl_3$, 298 K) of the purified striatal D (**9**).

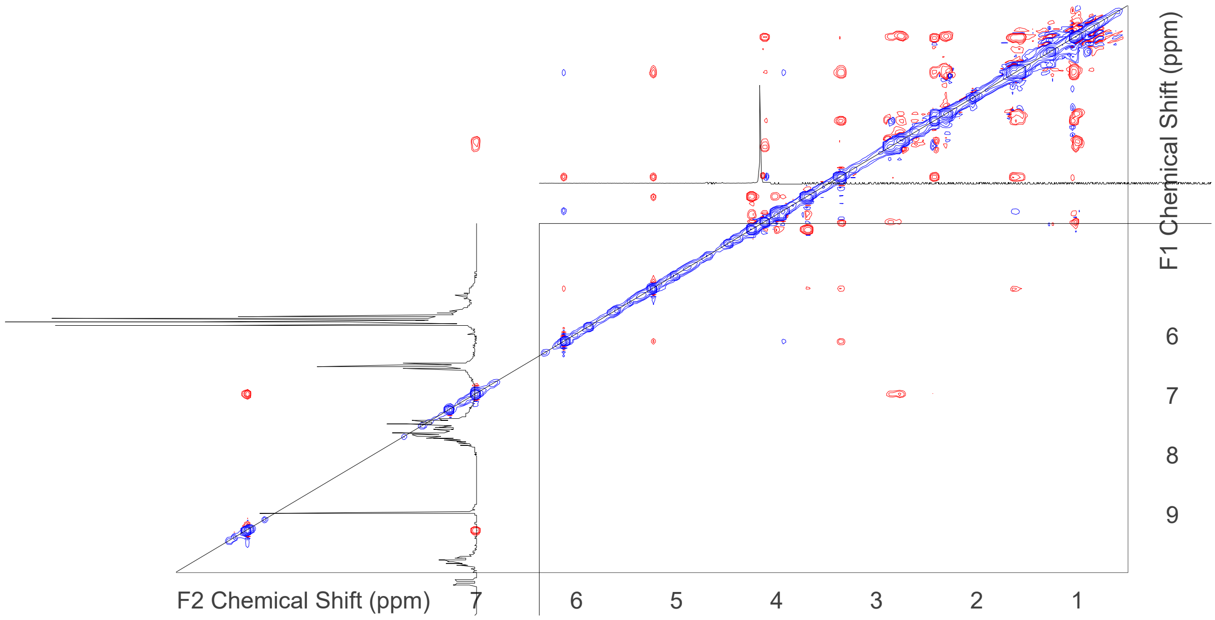

Figure S83: ^{1}H,^{1}H ROESY spectrum (500 MHz, $CDCl_3$, 298 K) of the purified striatal D (**9**).

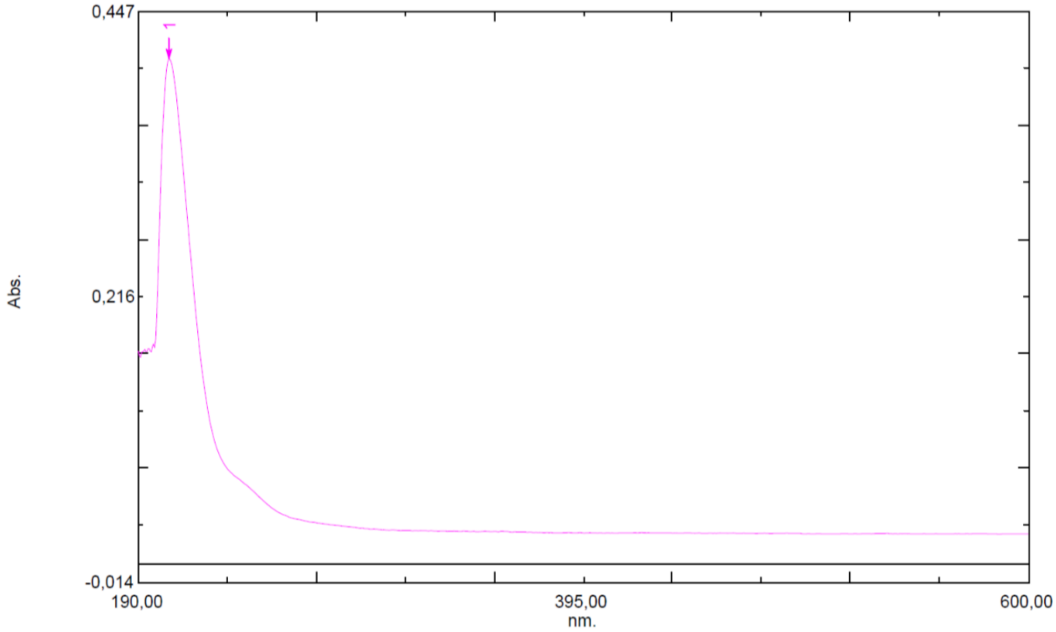

Figure S84: UV/VIS spectrum of compound **9** in MeOH.

(14) Analytical data of compound 10

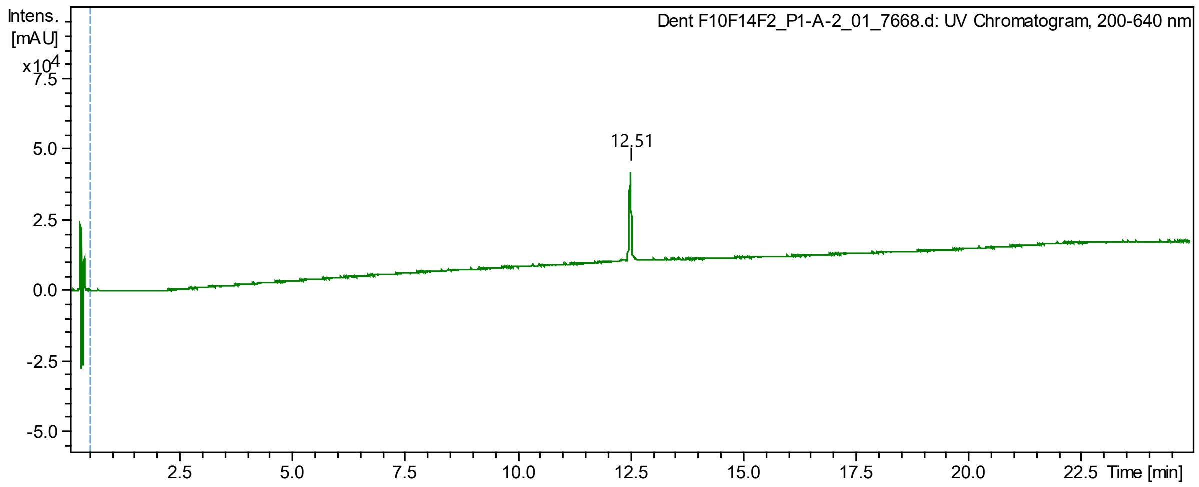

Figure S85: HPLC-DAD chromatogram of compound **10**.

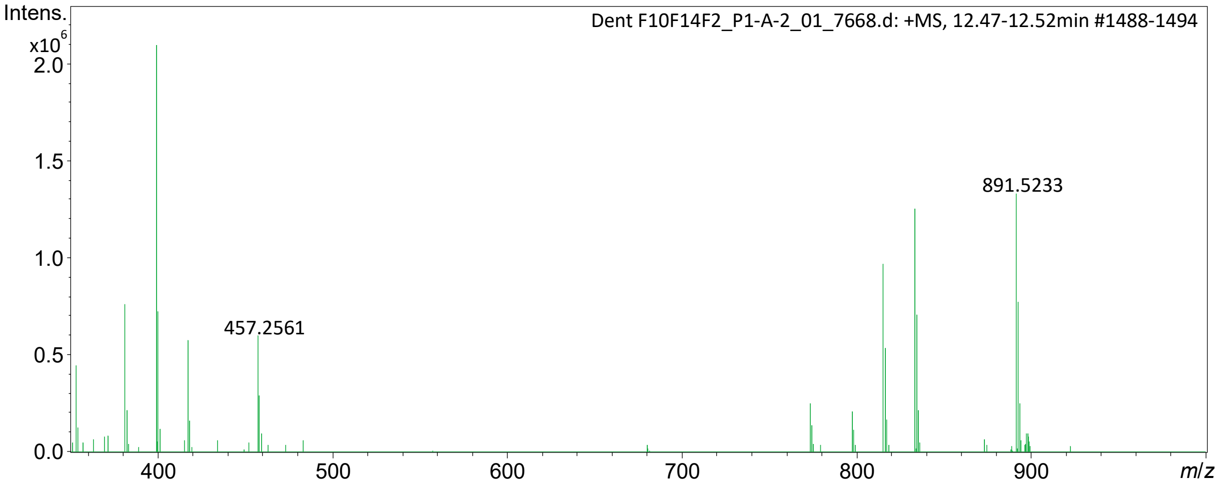

Figure S86: HR-(+)ESIMS spectrum of compound **10**.

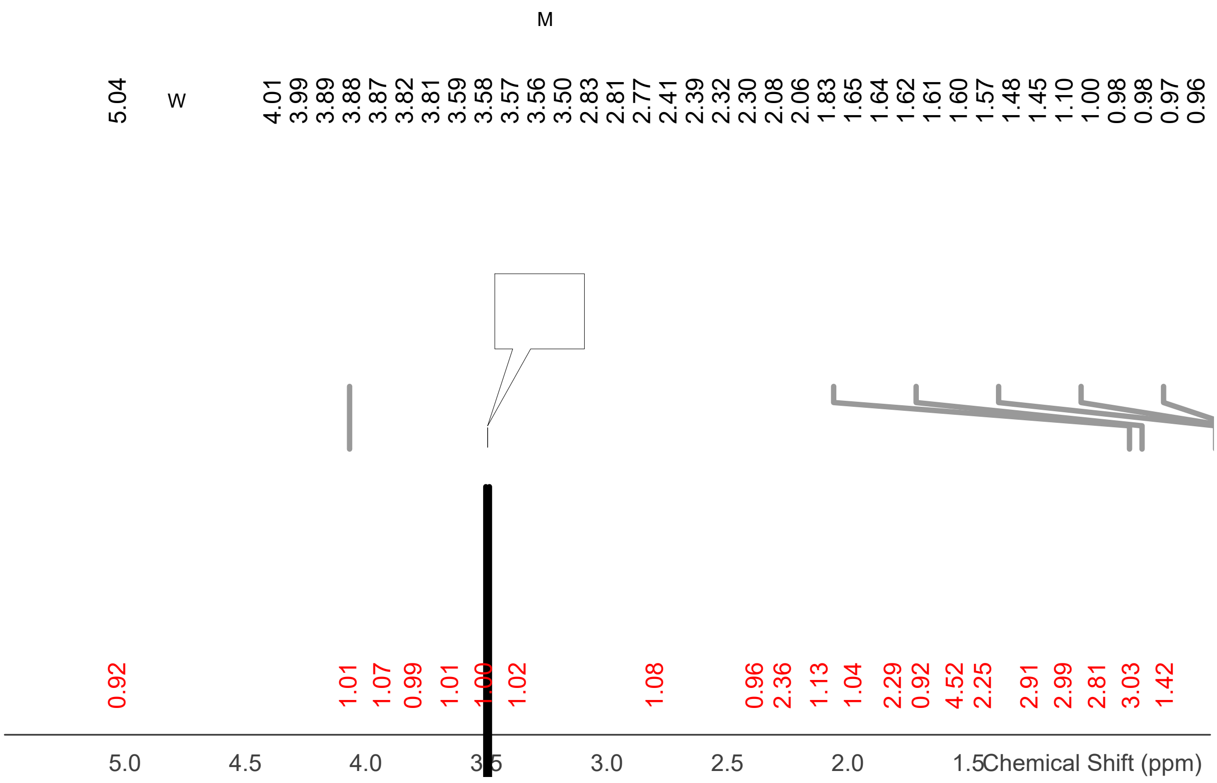

Figure S87: ^{1}H NMR spectrum (500 MHz, CD_3OD, 298 K) of the purified laxitextine A (**10**). W: H_2O, M: methanol.

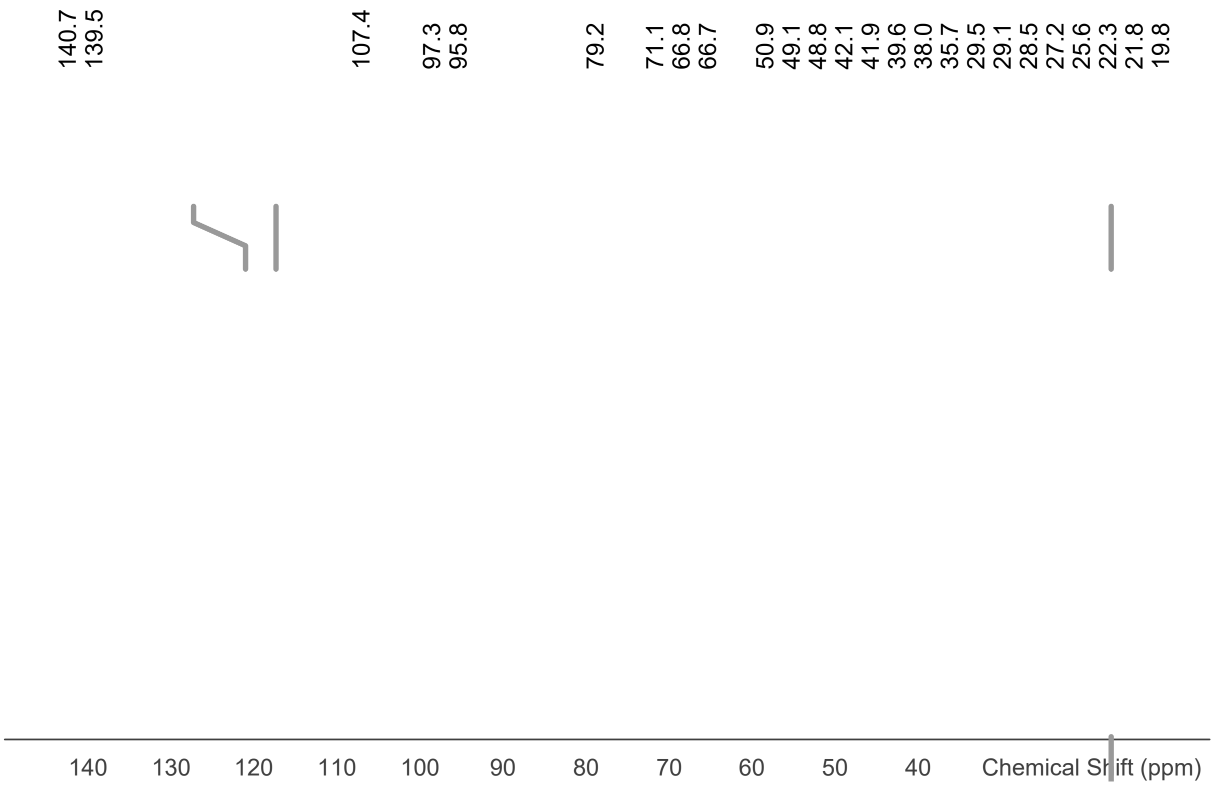

Figure S88: ^{13}C NMR spectrum (125 MHz, CD_3OD, 298 K) of the purified laxitextine A (**10**).

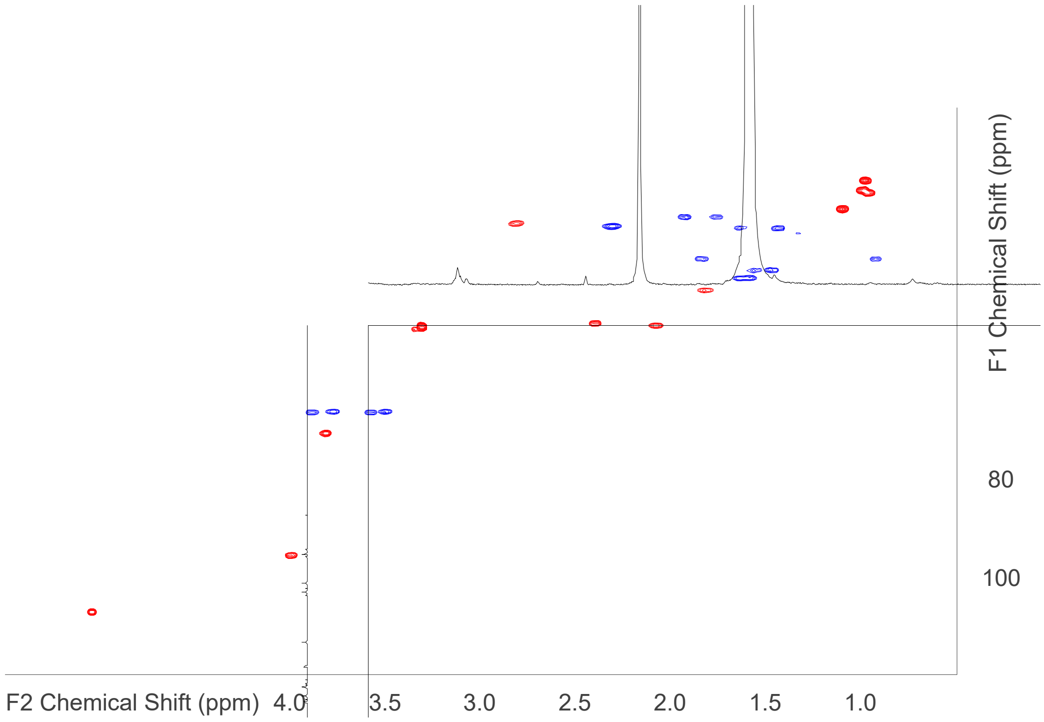

Figure S89: $^{1}H,^{13}C$ HSQC-DEPT spectrum (500 MHz, CD_3OD, 298 K) of the purified laxitextine A (**10**).

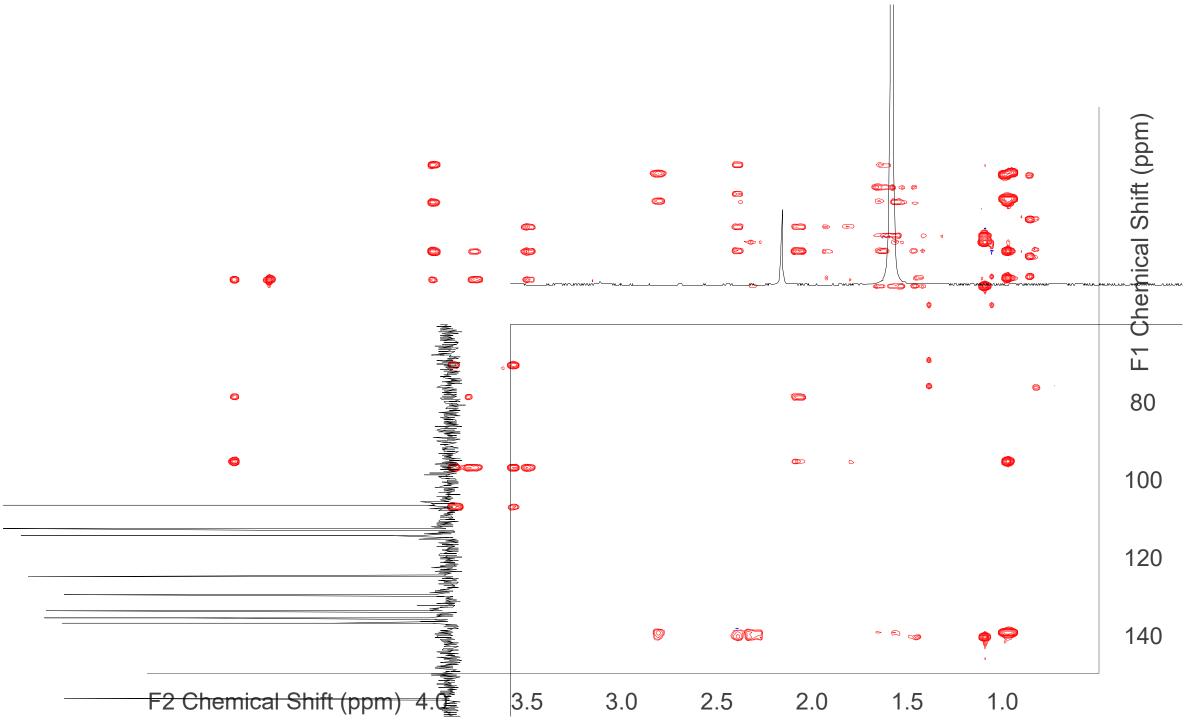

Figure S90: $^{1}H,^{13}C$ HMBC spectrum (500 MHz, CD_3OD, 298 K) of the purified laxitextine A (**10**).

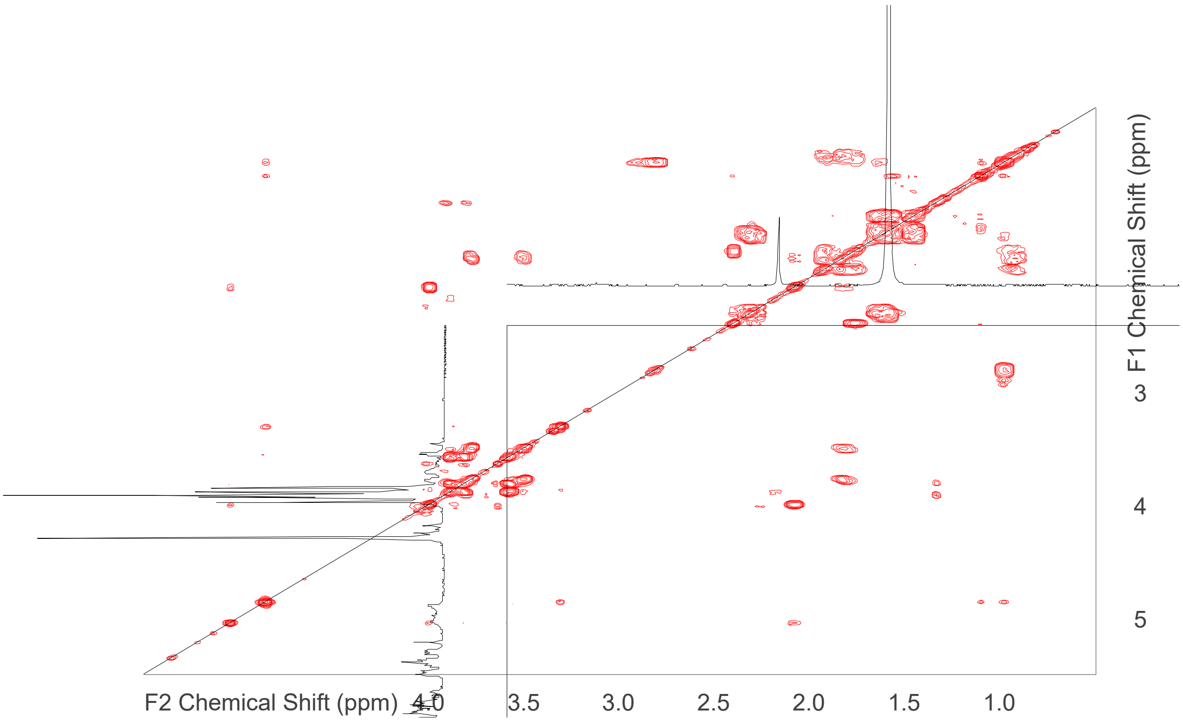

Figure S91: $^{1}H,^{1}H$ COSY spectrum (500 MHz, CD_3OD, 298 K) of the purified laxitextine A (**10**).

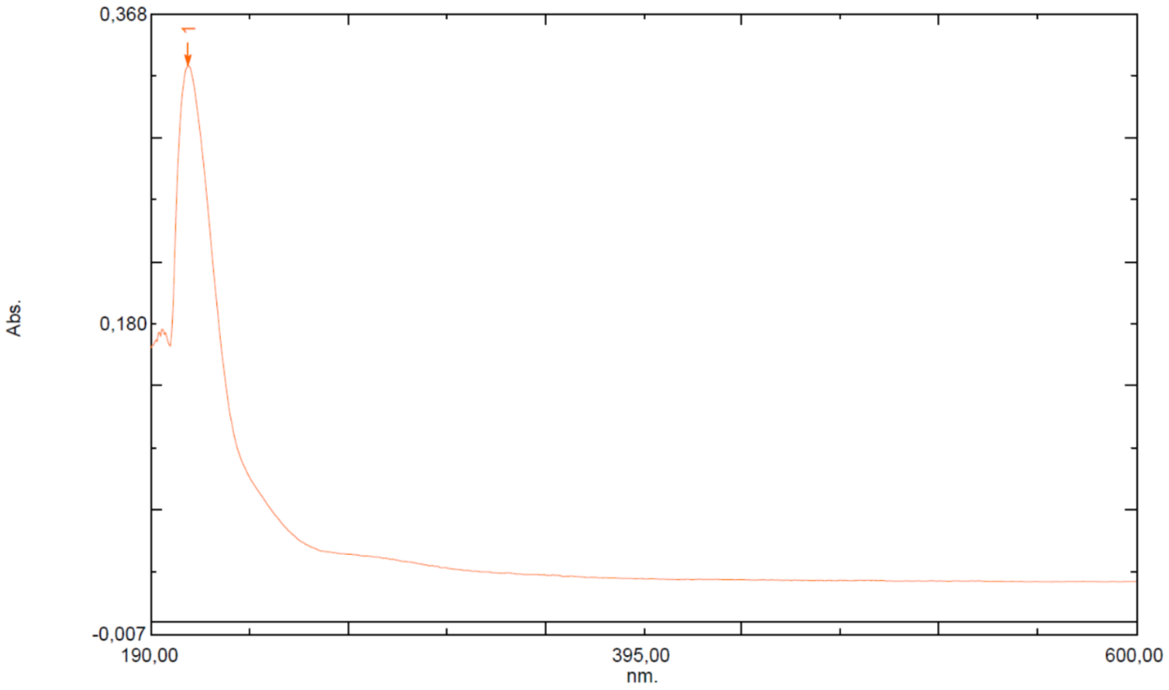

Figure S92: UV/VIS spectrum of compound **10** in MeOH.

References

1. Thompson, J.D.; Higgins, D.G.; Gibson, T.J. CLUSTAL W: Improving the Sensitivity of Progressive Multiple Sequence Alignment through Sequence Weighting, Position-Specific Gap Penalties and Weight Matrix Choice. *Nucleic Acids Res.* **1994**, *22*, 4673–4680, doi:10.1093/nar/22.22.4673.
2. Saitou, N.; Nei, M. The Neighbor-Joining Method: A New Method for Reconstructing Phylogenetic Trees. *Mol. Biol. Evol.* **1987**, *4*, 406–425, doi:10.1093/oxfordjournals.molbev.a040454.
3. Felsenstein, J. Confidence Limits on Phylogenies: An Approach Using the Bootstrap. *Evolution (N. Y).* **1985**, *39*, 783–791, doi:10.1111/j.1558-5646.1985.tb00420.x.
4. Tamura, K.; Nei, M. Estimation of the Number of Nucleotide Substitutions in the Control Region of Mitochondrial DNA in Humans and Chimpanzees. *Mol. Biol. Evol.* **1993**, *10*, 512–526, doi:10.1093/oxfordjournals.molbev.a040023.
5. Tamura, K.; Stecher, G.; Kumar, S. MEGA11: Molecular Evolutionary Genetics Analysis Version 11. *Mol. Biol. Evol.* **2021**, *38*, 3022–3027, doi:10.1093/MOLBEV/MSAB120.
